# Subpocket-Aware Fragment-Based Docking Pipeline for Kinase Ligand Design: Methodology and a PKA Case Study

**DOI:** 10.1021/acs.jcim.6c01373

**Published:** 2026-10-15

**Authors:** Katharina Buchthal, Paula Linh Kramer, Dominik Hubach, Gina Bach, Naemi Wagner, Johannes Krieger, Moritz Klöpper, Michael Daude, Wibke E. Diederich, Andrea Volkamer

**Affiliations:** † Data Driven Drug Design, Center for Bioinformatics, 14903 Saarland University, Campus E2.1, Saarbrücken 66123, Germany; ‡ 9377 Philipps-Universität Marburg, Fachbereich Pharmazie, Institut für Pharmazeutische Chemie und Zentrum für Tumor und Immunbiologie, Hans-Meerwein-Straße 3, Marburg 35043, Germany

## Abstract

Protein kinases regulate signal transduction through phosphorylation, and their dysregulation drives diseases like cancer, autoimmune disorders, and Alzheimer’s, making them key drug targets. Fragment-based drug design offers a powerful route to kinase inhibitors but often neglects kinase-specific structural insights. While KinFragLib, a library consisting of 7486 fragments derived from kinase-ligand complexes, provides a rich resource, exhaustive recombination of its fragments is computationally demanding. Here, we present an efficient exploration strategy that integrates subpocket-guided fragment growing with template docking to navigate this vast chemical space. Our automated pipeline prioritizes kinase-specific structural information, enabling targeted generation of chemically diverse ligands. Applied to the PKA kinase, the approach produced 33758 candidate molecules with promising docking scores, of which 15 were synthesized. Seven molecules reduced kinase activity by more than 50% at an inhibitor concentration of 100 μM, and five of these compounds also showed promising efficacy at a concentration of 10 μM. The IC_50_ determination ultimately revealed that five of the molecules were effective in the two- to three-digit nanomolar range. Crystallographic validation confirmed the predicted binding mode of the most potent compound. This methodology demonstrates how structure-informed guidance can transform fragment-based design into a scalable route for discovering novel kinase inhibitors.

## Graphical abstract

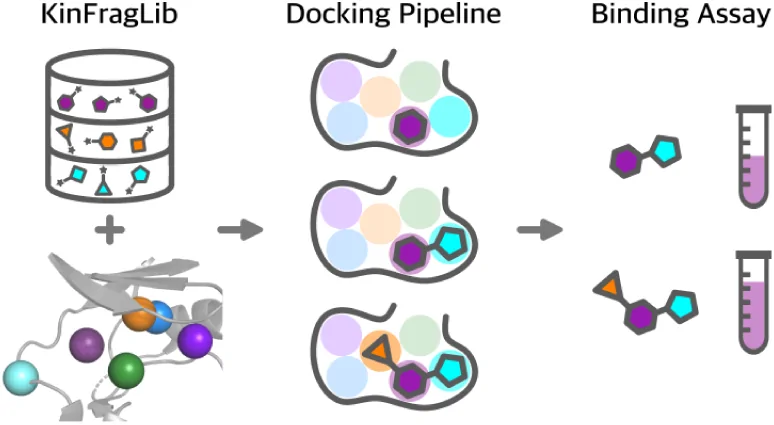

## Introduction

### Kinases as Drug Targets

With 518 different kinases, protein kinases are one of the largest superfamilies in the human genome.^1,2^ They play a central role in signal transduction in eukaryotic cells and regulate processes such as metabolism, transcription, differentiation, and apoptosis through phosphorylation.^1,3^ Dysregulation of protein kinase activity is often associated with diseases such as cancer or autoimmune disorders.^4,5^ Despite approximately 250 protein kinase inhibitors in clinical trials and the approval of to date 85 inhibitors by the US FDA, there is still a need for new kinase inhibitors. The approved drugs only target a fraction, i.e., about 20 kinases, of the large protein kinase family, and many kinases associated with diseases remain unaddressed.^6^ Additionally, drug resistance poses a significant challenge, limiting the long-term efficacy of these treatments.^6–8^ Human kinases can be categorized into eukaryotic and atypical kinases. Eukaryotic kinases, which are the focus of this study, have a high sequence similarity and share a common structure, consisting of a C-terminal and an N-terminal lobe connected by a hinge region. This highly conserved hinge region accommodates the ATP binding site.^1,9^ The structure of protein kinases comprises several structural motifs, including the front and back cleft, and the gate area (see Figure 1b). The front cleft contains the linker, glycine-rich loop, and hinge region. The hinge region is a crucial part of the binding pocket, anchoring the ligand through hydrogen bonds. The back cleft contains the α*C*-helix, which includes two conserved residues that can form a salt bridge, important for the activity of the protein kinase.^3,9,10^ The DFG loop, located in the gate area, can adopt two distinct conformations: DFG-in and DFG-out. The DFG-out conformation involves a conformational flip that extends the phenylalanine (F) of this 3-residue motive into the ATP-binding site, blocking ATP entry and resulting in an inactive kinase state.^3,9,10^

**Figure 1:**
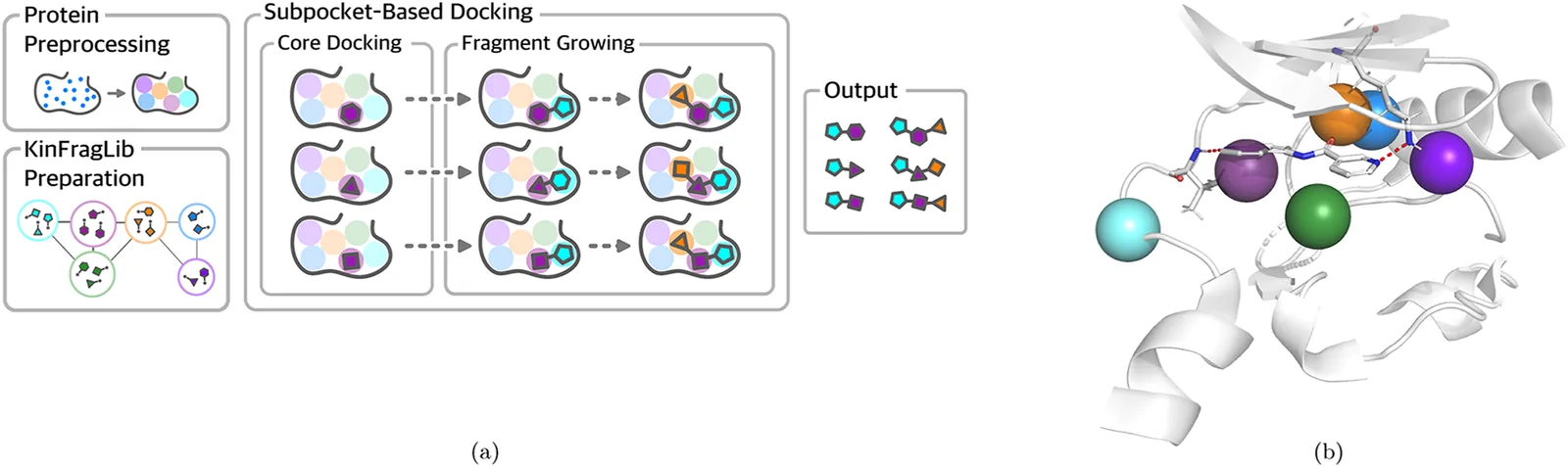
(a) Subpocket-based docking approach overview. First, protein preprocessing and KinFragLib^26^ library preparation are performed. Both the protein and the library are inputs for the subpocket-based docking pipeline. The pipeline consists of core docking of anchoring fragments, followed by iterative fragment growing using subpocket-based pharmacophore constraints. (b) Kinase binding pocket with subpockets. Cocrystrallized PKA with ligand 8ET (PDB code: 5N1F). All hydrogen bonds between the ligand and the protein are indicated in red. The colored spheres describe the centers of the six subpockets: AP—purple, FP—green, SE—cyan, GA—orange, B1—blue, and B2—violet.

Van Linden et al.^10^ developed the kinase database KLIFS comprising 6738 crystal structures (September 2025), assembled from the PDB. They defined the kinase binding pocket as consisting of 85 residues, based on an alignment of 1252 kinase-ligand crystal structures.^10^ The database provides the binding pockets, along with subpocket and binding mode annotations.

### Fragment-Based Drug Design

The vastness of chemical space, which refers to the immense number of potential compounds to explore, presents a significant challenge in drug discovery. This space encompasses an almost infinite number of possible compounds with an estimated size of around 10^60^.^11^ Recent large fragment spaces already span spaces of 10^10^ to 10^20^ compounds.^12,13^ Fragment-based drug design (FBDD) offers a practical solution to efficiently explore this space by using smaller molecular fragments,^14^ which can be combined to form compounds, thereby spanning large combinatorial spaces with a manageable number of fragments. FBDD involves linking, merging, or growing these fragments to design novel compounds. One of the earliest computational approaches in structure-based FBDD was GroupBuild.^15^ This method begins with either a user predocked core fragment or a user-defined and energetically favorable core fragment. It grows the core fragment using a rather small selection of building blocks. The candidates are then ranked using a force field-based scoring function. Huang et al.^16,17^ developed an FBDD strategy employing molecular docking to identify fragment poses, successfully generating inhibitors for targets like cyclin-dependent kinase 2 and β-secretase. However, their approach remains largely manual and tailored to specific targets. ACFIS—and ACFIS 2.0 ^18,19^ — is an online tool that automates core fragment identification and expansion but requires a protein structure with an existing ligand for core fragment selection. Penner et al.^20^ proposed using a Ray Volume Matrix (RVM) shape descriptor for fragment placement to overcome time-consuming docking. This method also requires specifying a core fragment and connection point. These advancements highlight the evolution and challenges in FBDD, paving the way for more efficient and automated approaches in kinase inhibitor design.

To discover ROCK1 kinase inhibitors, Beroza et al.^21^ posed a docking-based approach, using 71894 building blocks from the Enamine REAL Space^22^ as fragments. Fragment hits were identified by placing building blocks into the binding pocket and selecting 500 unique building block fragments after visual inspection. These fragment hits were then recombined with the building blocks following specific reaction rules and then docked using the fragment hits as templates, generating multiple potential inhibitors, each from two building blocks with *K*_*i*_ values < 10 μM and two crystallized structures confirming the docking poses. Müller et al.^23^ employed a similar approach using Enamine REAL Space building blocks and a docking-based approach to discover compounds for the cAMP-dependent protein kinase (PKA). In contrast to Beroza et al.,^21^ they utilized X-ray fragment hits of PKA as templates for fragment hit screening of the Enamine REAL Space building blocks. While a large chemical space was covered by using around 70k building blocks, these last two approaches require numerous manual steps. The CReM-dock^24^ pipeline automates an FBDD approach, prioritizing synthetic accessibility and diversity. It begins with placing core fragments, selected either automatically, e.g., from previously docked fragments using greedy, Pareto, or clustering-based selection techniques, or manually from user-defined X-ray structures. CReM^25^ is then iteratively applied to grow these fragments by replacing hydrogens with other fragments. Thus, docking is not used to position the newly grown fragments during this process to account for the binding pocket characteristics. In a case study, Minibaeva and Polishchuk^24^ applied CReM-dock to the CDK2 kinase, generating diverse compounds with high chemical novelty. Yet, their synthetic accessibility and target affinity remained unverified in experimental testing. Building on the large amount of (structural) data on kinases and the benefits of FBDD, Sydow et al. developed the kinase-focused fragment library KinFragLib^26^ to incorporate prior knowledge of kinase ligand binding modes during inhibitor design. Through data-driven fragmentation and recombination, KinFragLib expands and explores the kinase-specific chemical space. Cocrystallized kinase ligands were virtually fragmented according to their proximity in space to six predefined functionally relevant subpockets: adenine pocket (AP), front pocket (FP), solvent-exposed pocket (SE), gate area (GA), and back pocket 1 (B1) and 2 (B2) (see Figure 1b). Due to the functional distinction of these subpockets, fragments might provide subpocket-specific characteristics. This subpocket-based aspect can be useful for structure-based kinase inhibitor design, e.g., to reduce the search space or to increase the specificity.

### The Subpocket-Based Fragment Growing Methodology

In this study, we combine the advantages of FBDD with a computational structure-guided fragment growing approach. We incorporate prior knowledge about the kinase binding pocket and the characteristics of known kinase inhibitors to generate novel kinase-specific compounds. Thus, we grow ligands directly in the binding site using molecular docking, guided through subpockets annotated based on structural knowledge and using the KinFragLib^26^ library, which comprises fragments from cocrystallized kinase-ligand complexes.

Our pipeline, depicted in Figure 1a, automatically performs core fragment identification and stepwise fragment expansion, thus generating thousands of ligands based on a given kinase structure. Initially, fragments are placed into the binding pocket using the FlexX docking software^27^ to identify fragment hits. These fragment hits are then expanded stepwise by attaching fragments and growing the ligand using template docking.

The growing process employs a beam search technique, a greedy algorithm to heuristically solve optimization problems. In each layer of fragment expansion, we retain a fixed number of high-scoring partial results (fragments of increasing size) to achieve feasible computational times and avoid the full enumeration of combinational spaces. To incorporate the information on conserved regions within the kinase binding pocket, we utilize the subpocket information on KinFragLib’s predefined functionally relevant subpockets converted to pharmacophore constraints. This ensures fragments are placed only in their designated subpockets during docking.

As a case study, we applied our pipeline to the PKA kinase, a kinase commonly involved in various types of human cancer.^28,29^ We successfully generated 33758 molecules, of which 1196 candidates are predicted to have a good affinity of ≤1 μM, using the HYDE scoring function.^30^ 1154 compounds are considered potentially drug-like according to the Rule of Five^31^ and the Veber’s criteria.^32^ 94% of these compounds exhibited novelty (i.e., RDKit fingerprint Tanimoto similarity < 0.8) compared to ChEMBL33.^33^ We selected 11 molecules for experimental affinity determination. Small modifications and similar variants were introduced, resulting in 15 synthesized compounds. Seven of these reduced kinase activity by more than 50% at an inhibitor concentration of 100 μM. Five molecules show promising activity at 10 μM and IC_50_ determination revealed an IC_50_ below 1 μM for these five compounds, suggesting strong potential starting points for kinase inhibitor discovery. The identification of multiple nanomolar inhibitors from only 15 synthesized compounds compares favorably with outcomes reported in successful prospective docking campaigns^21,34,35^ and supports the practical utility of the workflow. Our automated pipeline facilitates the discovery of novel hit molecules for novel protein kinase targets built from the KinFragLib library. The pipeline can be applied to any protein kinase of interest and adjusted to accommodate potential structural differences. The subpocket-based docking pipeline is accessible on GitHub (https://github.com/volkamerlab/KinFragLib_PocketEnum).

## Results and Discussion

Here we present the results of our automated pipeline for iterative fragment growing within the binding site. First, the pipeline is introduced, followed by the specific setup for the PKA case study. Through iterative fragment selection, the workflow substantially reduced the combinatorial search space while prioritizing promising candidate ligands. We then analyzed the chemical space of the generated compounds, and applied a series of filtering steps to identify promising candidates for synthesis. A total of 15 compounds were synthesized and tested for residual kinase activity, as described in the final sections.

### Automated Subpocket-Based Docking Pipeline

We introduce a workflow that performs subpocket-based docking to generate potentially novel kinase ligands for a given target structure. This workflow consists of the following steps, as depicted in Figure 1a:

1 Definition of the binding pocket and pharmacophore constraints of each subpocket in SeeSAR.^36^
2 Preprocessing and filtering of the KinFragLib fragment library.
3 Placement of initial fragments into a user-predefined core subpocket.
4 Appending fragments and performing template docking on the next user-predefined subpocket.
5 Repeat step 4 until all predefined subpockets have been covered.

While the first step must be performed as a manual preprocessing step, steps 2–4 are part of the automated subpocket-based docking pipeline. In the core docking step (3), the initial fragments are placed into the core subpocket through pharmacophore constraints in the FlexX docking software.^37^ To avoid combinatorial explosion during fragment growing, we limit the number of candidates to consider in the subsequent steps in a greedy manner based on the predicted affinity score, and—if desired—molecular diversity. The selected candidates are expanded by adding fragments iteratively based on the predefined order of subpockets. A template docking strategy is applied to keep the core fragment in the core subpocket while placing the appended fragment in the new subpocket as shown in Figure 1a (step 4). In each iteration, we again retain a fixed number of candidates to be considered in the next fragment growing iteration. More details are described in Materials and Methods. To allow the user to customize the subpocket-based docking on a kinase target of interest, a JSON definition file needs to be passed. It enables the configuration of multiple program parameters, such as the core subpocket, the subpocket path for fragment growing, or the number of fragments and poses, which should be chosen during docking pose filtering.

### Case Study on Hamster PKA

As a proof of concept, we applied the subpocket-based docking pipeline to the hamster PKA structure (PDB code: 5N1F), following preprocessing as described in Materials and Methods. Generated ligands were evaluated for drug-likeness, diversity, and novelty. A subset of promising compounds was selected for synthesis and experimental validation. The following sections detail the docking setup, chemical space analysis, compound filtering, and experimental procedures.

#### Program Setup

For this study, we used the default program configuration. We set the number of molecular candidates to select for further growing per subpocket iteration to 300 and the number of chosen docking poses to consider in the next iteration per candidate to 5. For candidate selection, we used the cluster-based approach, which ensures some diversity among the chosen candidates rather than optimizing only for the estimated binding affinity. The details of our selection strategies are explained in Materials and Methods. For the docking setup, we selected the AP subpocket as the core and defined a fragment-growing path through the FP and GA subpockets, guided by the cocrystallized ligand in the PKA structure (PDB code: 5N1F; see Figure 1b). The ligand occupies the AP subpocket and is located near FP and GA, which were included in the design to reflect the spatial context of the native ligand. More details on the subpocket definitions are described in Materials and Methods. The JSON file, defining the program setup, is given in Supplementary Text S10.

The fragment library used as a starting point is the kinase-focused library KinFragLib,^26^ which consists of 9505 fragments. To reduce library size and improve suitability for synthesis, we applied additional filtering using CustomKinFragLib:^38^ PAINS^39^ and Brenk^40^ filters were used to exclude undesirable substructures. Drug-likeness was assessed via the Rule of Three^41^ and QED^42^ score, while synthesizability was evaluated using the SYBA^43^ score. For more information on the filtering criteria, we refer to the CustomKinFragLib publication.^38^ After filtering, the fragment library was reduced to 1363.

The subpocket-based docking pipeline was executed with the specified configuration on a computing cluster using 128 CPUs. The total runtime in this setup was approximately 17 h with close to 60000 enumerated molecules.

#### Chemical Space Analysis of Generated Ligands

KinFragLib’s^26^ chemical space comprises approximately 470 million molecules that consist of fragments covering either the AP, FP, and GA, or only the AP and FP subpockets. After fragment library filtering with CustomKinFragLib,^38^ the fragment recombination space based on the remaining fragments covers approximately 22 million molecules (see Figure 2a). From this space, we enumerated—i.e., recombined—59255 molecules with our docking pipeline. 33758 of these enumerated molecules could be successfully docked and are considered as the output of the subpocket-based docking pipeline. We will refer to these compounds as generated compounds. Note that all these generated compounds consist of fragments of either all traversed subpockets—AP, FP, and GA—or only the AP and FP subpockets.

**Figure 2:**
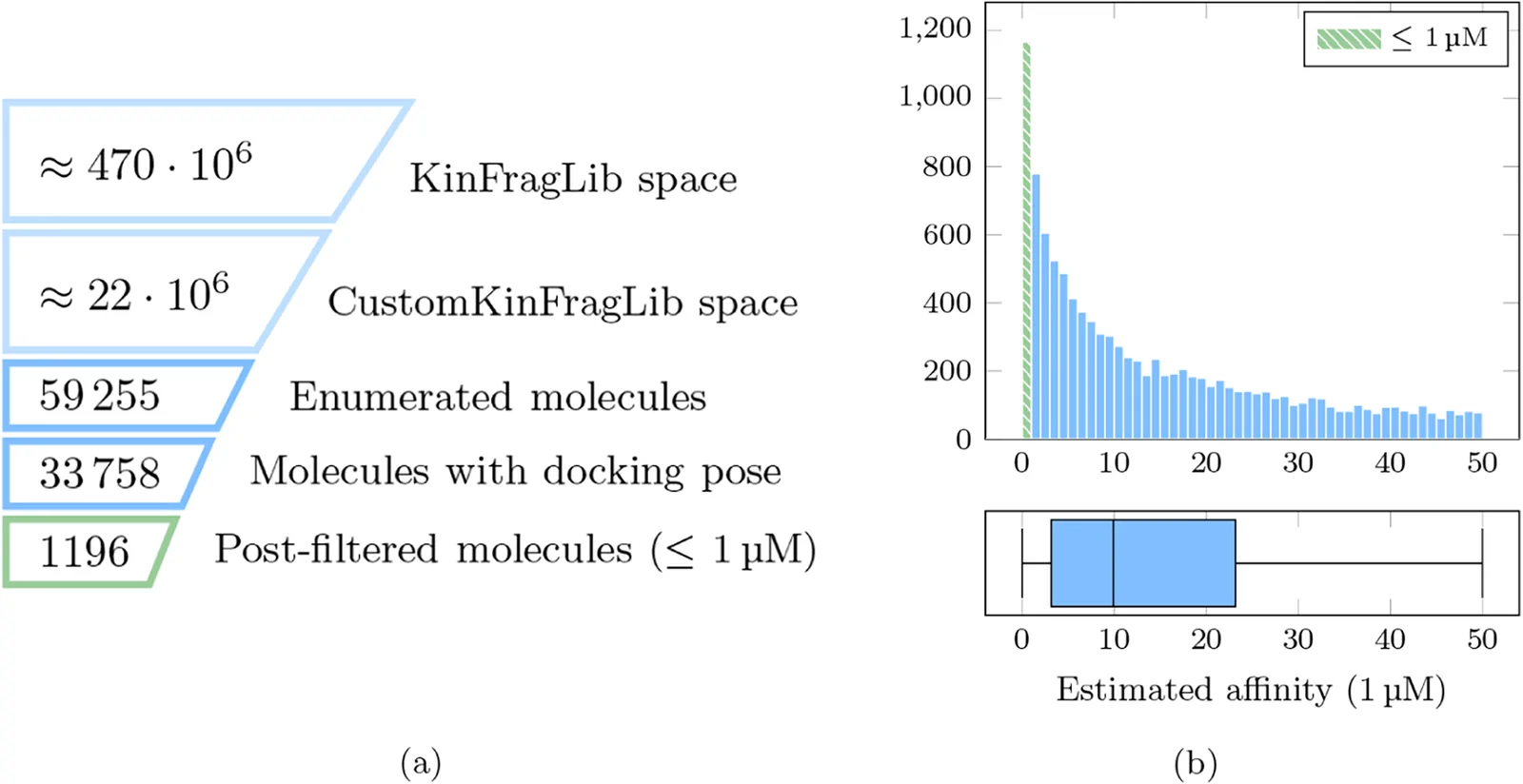
(a) Quantities of explored chemical space during the individual steps: light blue: combinatorial library estimates; blue: generated and docked molecules; green: selected high-scoring molecules. (b) HYDE scores of 11159 high-scoring molecules (generated compounds with HYDE score ≤ 50 μM).

Starting from the 33758 docked compounds, we first applied a HYDE score threshold of ≤50 μM, yielding 11159 candidates with moderate to good predicted affinities, with an average score around 10 μM (see Figure 2b). To further enrich for promising candidates, we retained only compounds with predicted affinities of ≤1 μM, resulting in 1196 prioritized molecules. As compound prioritization relies on predicted HYDE affinities, the results are naturally influenced by the performance of the scoring function. However, HYDE has been reported to provide accurate affinity estimates across diverse protein–ligand complexes, making it a suitable choice for this application. Its computational efficiency further enables the rapid exploration and screening of large chemical spaces while maintaining manageable computational costs.

Next, we analyze the 1196 postfiltered compounds regarding diversity, novelty, and drug-likeness. Diversity is assessed based on scaffold composition and molecular fingerprints, as described in Supplementary Texts S6–S7.

We first determined the Murcko scaffolds^44^ of all postfiltered compounds. This resulted in 800 unique scaffolds, of which 611 (76.4%) are singletons. A singleton refers to a scaffold that matches exactly one compound. Accordingly, a majority of the compounds do not share a common scaffold, indicating diversity within our high-scoring compounds. We further inspect the distribution of scaffold frequencies of scaffolds, disregarding singletons in Figure 3a. The most frequent scaffold—shown in Figure 3a—represents 2.3% (or 27 molecules) of all postfiltered compounds. Furthermore, 162 scaffolds—i.e., 86.2% (disregarding singletons)—are shared with only a small number (2–3) of compounds. Calculating the scaled Shannon Entropy (SSE) for the scaffolds revealed a score of 0.96 indicating high entropy, hence a substantial structural diversity within the data set. For more details on the SSE, we refer to Supplementary Text S6.

**Figure 3:**
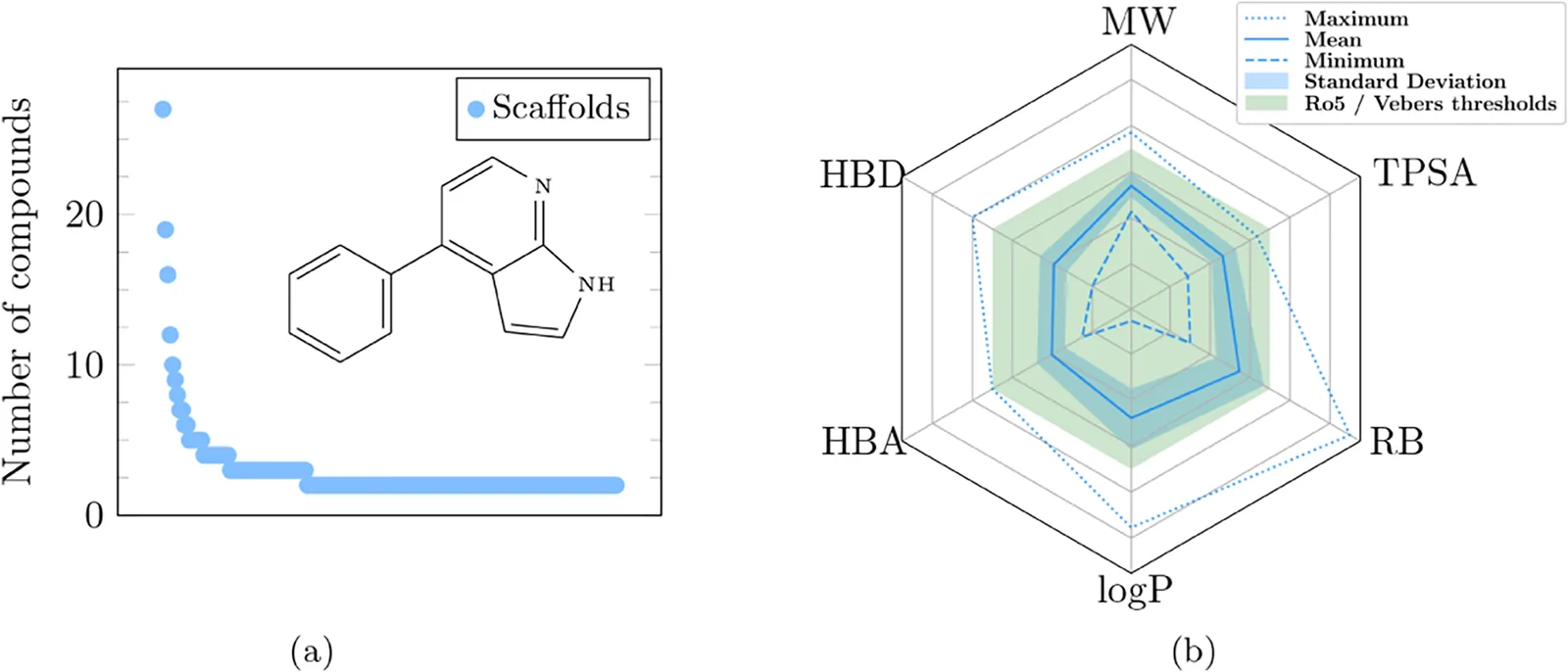
(a) Distribution of the scaffolds disregarding singletons. The most common scaffold is depicted. (b) Molecular properties of postfilter PKA compounds. Some properties were scaled for visual purposes. Green indicates the area of favored property values.

To further characterize compound diversity, we evaluated pairwise molecular similarity using Tanimoto coefficients derived from RDKit topological fingerprints.^45^ For each compound, we determine the Tanimoto similarity to its nearest neighbor, i.e., the most similar compound in the data set (see Supplementary Figure S3A). While 177 compounds exhibit nearest-neighbor similarity of 0.9 or above and 534 compounds ≥ 0.8, the slight majority of compounds (54.2%) show similarity values below 0.8. This distribution suggests a high degree of structural diversity among the ligands, as most compounds are rather dissimilar from their closest counterparts.

#### Novelty and Druglikeness of Generated Ligands

A comparison against the 2372672 compounds available in ChEMBL33 ^33^ was performed to assess the novelty of the generated compounds. For each of the postfiltered PKA compounds, we identified the most similar ChEMBL ligand using the Tanimoto similarity based on the topological fingerprints.^45^ Only one of the 1196 analyzed compounds exhibits a Tanimoto similarity of 1.0, i.e., an exact match with the compound CHEMBL597211.^46^ Among the 39 listed bioactivity entries in ChEMBL, this compound was experimentally tested against a human PKA kinase target (assay ChEMBL ID: CHEMBL1074363 ^47^ and shows 48% inhibition at 1 μM concentration. All other kinase targets this compound was tested against are listed in Supplementary Table S4. Additionally, a crystal structure of this ligand bound to AKT2 (PDB code: 2XH5) shows a substantial overlap in binding mode compared to our predicted pose (see Figure 5a). More details can be found in Supplementary Text S1.

In total, 11 compounds (0.9%) show a Tanimoto similarity (on RDKit topological fingerprints^45^ of ≥0.9 (see Supplementary Table S4) while 5.9% of the compound exhibit a Tanimoto similarity of ≥0.8. Thus, over 94% of the generated compounds represent novel chemical matter compared to ChEMBL compounds considering a similarity threshold of 0.8. Further, most of the generated compounds show a moderate similarity to their closest ChEMBL counterpart, with a mean similarity of 0.63 ± 0.1, as shown in Supplementary Figure S3B. In comparison to known PKA ligands, we also see a low Tanimoto similarity, hence some degree of novelty among the generated ligands. For more details on the PKA ligand analysis, see Supplementary Figure S4.

To assess the drug-likeness of the postfiltered compounds, we consider the molecular properties from the Rule of Five (Ro5) and Veber’s criteria:^31,32^ molecular weight (MW), hydrogen-bond donors (HBD), hydrogen-bond acceptors (HBA), logP, topological polar surface area (TPSA), and rotatable bonds (RB). 93% of all postfiltered compounds fulfill the Lipinski’s Rule of Five as well as the Veber’s criteria (see Figure 3b). More details can be found in the Supplementary Text S9.

#### Compound Postselection and Synthesis

To translate computational predictions into experimentally testable candidates, we manually selected compounds for synthesis from the set of 1196 high-scoring compounds (see Figure 2a). To ensure synthetic tractability, compounds containing chiral centers were excluded, resulting in a subset of 815 compounds. Of these, the 200 best compounds, based on the predicted binding affinity calculated with HYDE,^30^ were subjected to further visual inspection, with particular attention paid to synthetic feasibility. In addition to limiting the number of reaction steps to a maximum of three, without taking protective group chemistry into account, the commercial availability of the respective starting materials was also taken into consideration.

In the end, this postselection process led to the identification of 11 compounds that were considered suitable candidates for synthesis (see Figure 4). However, as some starting materials were regarded as too expensive, slight changes were made, and the modified molecules were rescored with HYDE (see Figure 4). Most of the modifications led to only minor changes in predicted HYDE affinity, while only compound **7** showed a larger deviation compared to the proposed one (9167.72 nM and 22.41 nM, respectively). We inspected the docking pose of the proposed molecule (L03700308) compared to the adapted molecule (**7**) (see Supplementary Figure S1) and found that both molecules overlap strongly and are predicted to have two hydrogen bonds with the hinge region. As the pose was still considered reasonable, the compound was retained for synthesis and subsequent testing. Four compounds (**8**, **9**, **17**, and **20**) could be synthesized as originally proposed. In four other cases, (halogen) substituents were replaced by a hydrogen atom (**24**, **26**, **30**, and **33**), thereby significantly reducing the possibility of side reactions during synthesis (see Figure 4). The synthesis of most of the selected compounds was straightforward; however, in the case of **40** the yield was low (4%) and, moreover, the compound could not be obtained in a purity suitable for further testing, thus, it was not considered further. In the course of the synthesis of compound L00710372, it became apparent that the purine derivative could not be synthesized under the chosen reaction conditions. Therefore, the nitrogen atoms, which according to the docking pose (Supplementary Figure S2), do not interact with the hinge region, were exchanged for carbons, resulting in compound **39b**. In compound **39a**, again the halogen substituent was replaced by a hydrogen atom. In addition to these two derivatives and their synthetic precursors (**38a** and **38b**) of L00710372, also the pyrrolo-pyrimidine derivatives of **38a** and **38b** (**38c** and **38d**) were synthesized. More details on the synthesis of these compounds are given in Materials and Methods as well as the Supplementary Information.

**Figure 4:**
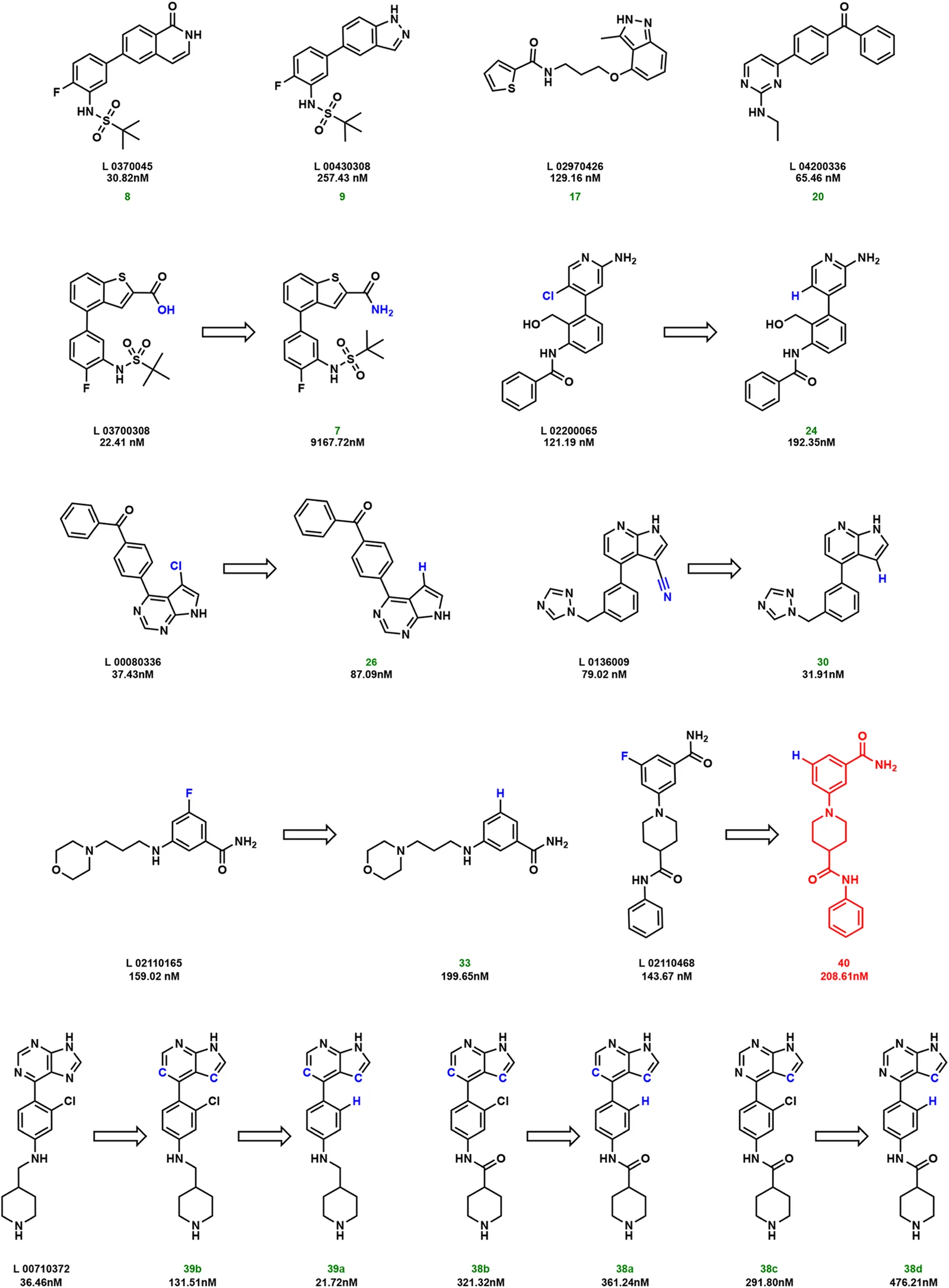
Overview of the selected ligands (black) and the predicted affinities. Changes to the predicted molecules are highlighted in blue, in-house synthesized compounds are labeled with green compound names and the predicted affinity. The molecule marked in red could not be successfully synthesized in sufficient purity for further testing.

#### Experimental Assay Results

The affinity of the synthesized molecules was evaluated in-house using a KinaseGlo Luminescent Kinase Assay Kit, where the affinity is expressed as residual PKA activity (%) at a specific compound concentration. Prescreening at an inhibitor concentration of 100 μM revealed that seven of the 15 compounds tested met our cutoff criterion of reducing kinase activity by significantly more than 50% (see Figure 5c). The kinase residual activity at 100 μM and the corresponding estimated affinity calculated by HYDE are depicted in Supplementary Figure S5A. The analyzed compounds were selected from the top 200 candidates based on HYDE scoring followed by manual prioritization, including visual inspection and medicinal chemistry considerations, and therefore do not represent an unbiased sample of the ranked compound set. Consequently, this exploratory analysis is descriptive and is not intended to assess the performance of HYDE, particularly given the limited sample size (*n* = 15). For the seven promising compounds, residual protein activity was determined at three different compound concentrations: 100 μM, 50 μM, and 10 μM (Figure 5d), with **30** being the most active one with a residual kinase activity of only 14.2% ± 2.3 at 10 μM ligand concentration. Somewhat surprisingly, the synthetic precursors **38b**, **38a**, **38d**, and **38c** also showed promising activity at 10 μM, with **38d** showing activity to a lesser extent. Compounds bearing a pyrrolo-pyridine moiety are generally more active than their pyrrolo-pyrimidine counterparts (**38b** vs **38d** and **38a** vs **38c**). Interestingly, in the case of the pyrrolo-pyridine series, the influence of the chlorine substituent on affinity is only minor, whereas it is more pronounced in the compounds **38d** and **38c**, with the originally proposed compound being less active in this case. In total, five compounds showed promising activity at a concentration of 10 μM, exhibiting residual kinase activity of approximately 25% or less. The IC_50_ values of the most promising compounds from the initial screening were subsequently determined using a radiometric assay at Eurofins (see Figure 6 and Table 1). However, human PKA was used instead of hamster PKA. Compared to the data on percentage inhibition, this resulted in minor changes in the inhibitors’ ranking. These changes can be attributed to two factors: using PKAs from two different species and changing the assay methodology. Nevertheless, the order of the inhibitors within a series remained unchanged. The IC_50_ values and the corresponding estimated affinity calculated by HYDE are depicted in Supplementary Figure S5B. These compounds were selected due to a kinase residual activity below 25% at 10 μM and therefore do not represent an unbiased sample of the ranked compound set. Consequently, this exploratory analysis yielded no statistically significant correlation and should be considered descriptive rather than an independent validation of HYDE, particularly given the limited number of tested compounds (*n* = 5).

**Figure 5:**
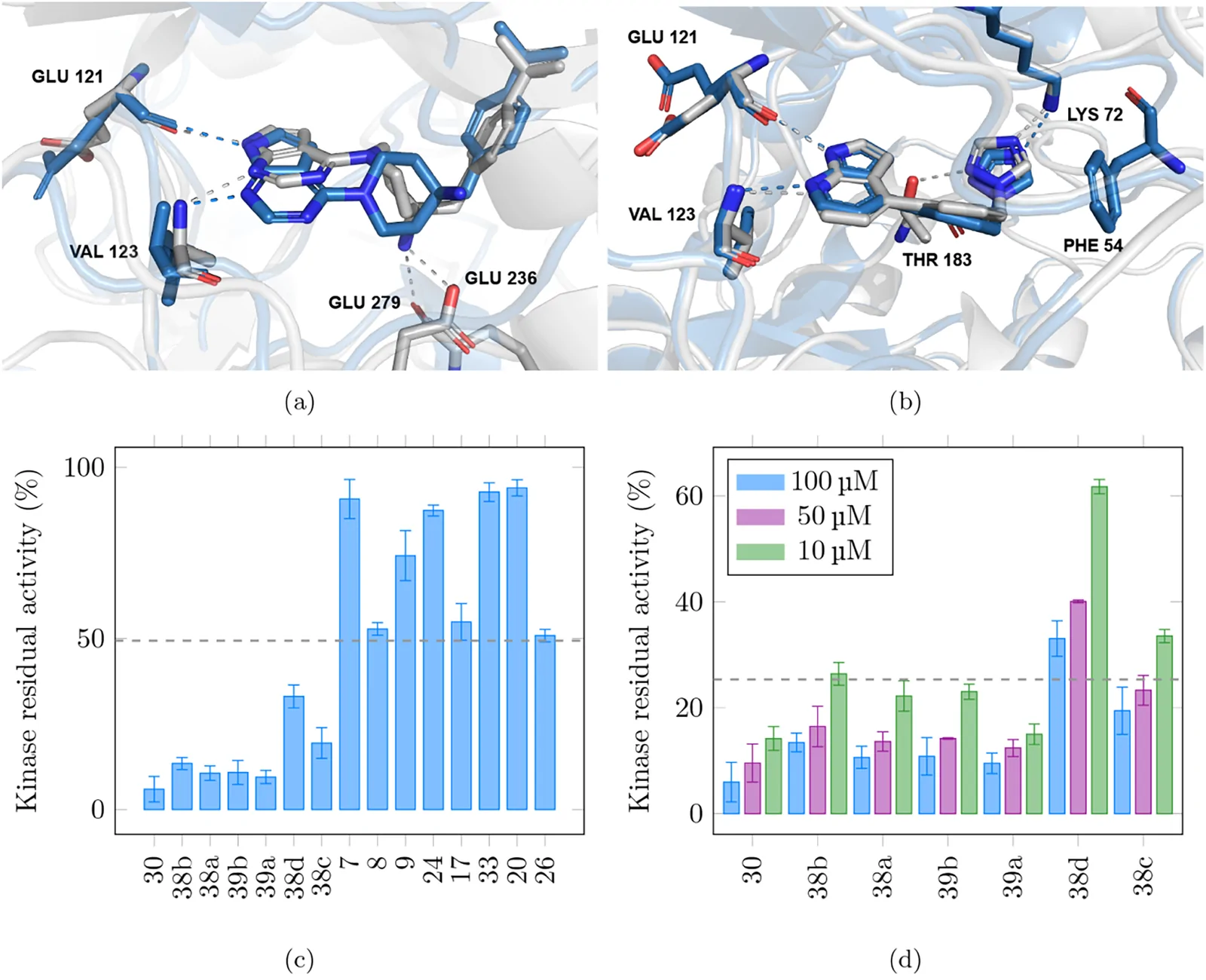
(a) Crystal structure of CHEMBL597211 compound (PDB: 2XH5, gray) bound to AKT2 kinase aligned with the PKA kinase (PDB: 5N1F, blue) with our docking pose. Hydrogen bonds are indicated with dashed lines in the respective colors. (b) Cocrystallized structure of compound **30** with PKA (PDB: 9TEC, blue) and corresponding docking pose (PDB: 5N1F, gray) aligned. Glutamate 121 has been observed in two different conformations in 9TEC. Hydrogen bonds are indicated in the respective colors. (c) Residual kinase activity (%) of PKA for all synthesized ligands measured at 100 μM compound concentration. Dashed line indicates 50%. (d) Residual kinase activity for the most promising synthesized ligands at three compound concentrations. Dashed line indicates 25%.

**Figure 6:**
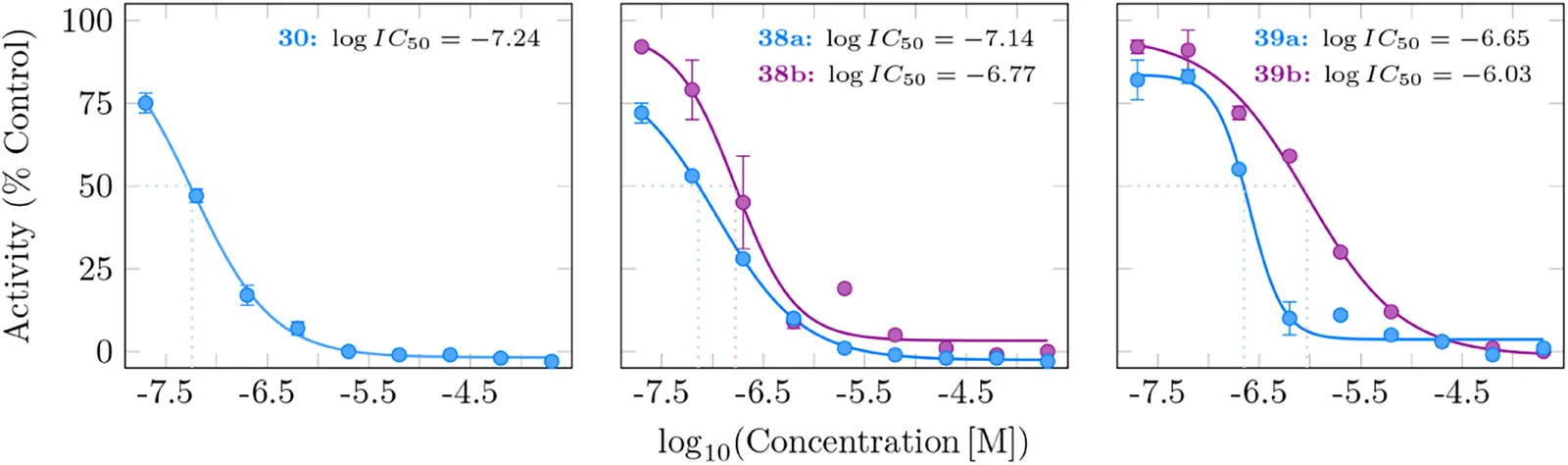
Dose response curves for compounds **30**, **38a**, **38b**, **39a**, and **39b**.

**Table 1:** IC_50_ Values [μM] of Compounds **30**, **38a**, **38b**, **39a**, and **39b**

Compound	IC_50_
**30**	0.058 μM
**38a**	0.072 μM
**38b**	0.168 μM
**39a**	0.222 μM
**39b**	0.940 μM

#### Crystallization

We cocrystallized the most promising compound (**30**) with PKA (PDB code: 9TEC) and present a structure with a resolution of 1.38 Å. When aligning our crystal structure to the PKA structure used for docking (PDB code: 5N1F), the crystallized ligand matches the predicted docking pose well (see Figure 5b). We observed four different hydrogen bonds. Two with the side chain of lysine 72, threonine 183, two with the backbone of glutamate 121 and valine 123. Additionally, a T-shaped π–π interaction involving phenylalanine 54 can be observed in the X-ray structure. The absence of this interaction in the docking pose can be attributed to the rigid protein structure employed during docking with FlexX.

## Conclusion

Here, we introduce an automated fragment-based pipeline that efficiently explores a kinase-specific combinatorial space to generate hit molecules. By integrating iterative fragment growing with subpocket-guided template docking, the approach leverages kinase domain knowledge for targeted ligand design.

Applied to PKA as a model target, the pipeline filtered the combinatorial space covering ≈22 million molecules and identified around 1200 promising candidates with predicted affinities below 1 μM. After visual inspection of the 200 top-ranked candidates, 11 molecules were selected for synthesis. Ultimately, 15 compounds were synthesized via short, straightforward synthetic sequences, five show promising activity with IC_50_ values in the two- to three-digit nanomolar range. X-ray crystallography of the most active compound confirmed substantial overlap between the predicted and experimental binding modes. This pipeline can be adjusted and applied to any typical kinase target of interest, as long as a crystal (or predicted) structure is available. The setup, such as the choice of subpockets, can be tailored by the user to include available kinase knowledge.

A limitation of the present study is that kinase selectivity was not explicitly studied. While the workflow incorporates kinase-specific subpocket information during ligand design, the highly conserved nature of ATP-binding sites across the kinome means that selectivity cannot be assessed from docking against a single target alone. The current proof-of-concept study focuses on the efficient identification of kinase-binding hit molecules and the validation of predicted binding modes. Future work should therefore include profiling of generated compounds against representative kinase panels to assess selectivity and investigate to what extent subpocket-guided design can be leveraged to bias compounds toward specific kinase targets.

The PKA case study demonstrates that our structure-informed pipeline enables scalable fragment-based design and delivers hit molecules with IC_50_s as low as 58 nM as valuable starting points for further optimization.

## Materials and Methods

### Kinase Fragment Data

The fragment library KinFragLib v2.0.0 ^26^ comprises 9505 fragments derived from 3232 cocrystallized kinase-ligand complex structures from the KLIFS database^10^ (queried December 2023). KinFragLib splits these ligands into fragments using the retro-synthetically inspired BRICS decomposition rules.^48^ These fragments are then annotated based on their spatial proximity to six predefined subpockets. Neighboring fragments annotated with the same subpocket are merged. The subpockets—namely AP, FP, GA, SE, B1, and B2—were defined based on functionally relevant regions within the kinase binding pocket, as derived from literature.^10^ Fragments that could not be assigned to any of the six subpockets were assigned to a pool X. During fragmentation, bonds that are split are replaced with dummy atoms at the cleavage site, with each dummy atom retaining information about the subpocket originally connected to the removed fragment. These dummy atoms will be used as attachment points, and the subpocket information is needed to preserve the original subpocket connections. Since the recombination space of these fragments is too large to exhaustively enumerate, we restrict our analysis to promising fragments. Therefore, we reduce the fragment library using CustomKinFragLib.^38^ CustomKinFragLib is an extension of KinFragLib^26^ that implements several filters aiming to reduce the fragment space of KinFragLib. We reduce the fragment library from 9505 to 1363 fragments after applying CustomKinFragLib filters targeting (1) prefiltering (redundancy), (2) unwanted substructures, (3) drug-likeness, and (4) synthesizability. For details on the applied filters and parameters, together with the number of fragments removed per filter, refer to Supplementary Table S3. Note that since the filtering process is integrated into the pipeline, users can select which filters to apply along with their respective parameters.

### Subpocket-Based Fragment Docking and Growing

Aiming to generate potential kinase inhibitors, we recombine the fragments from the custom-filtered fragment library KinFragLib.^26^ However, enumerating all fragment combinations—and then docking the resulting ligands—would not be computationally feasible. Thus, we divide the problem into a step-by-step fragment docking and growing process guided by placing fragments into distinct subpockets, followed by an elaborate pose and fragment selection strategy. We use the docking software FlexX,^27^ which allows for pharmacophore- as well as template-guided docking.

This subpocket-based docking approach is implemented in a Python pipeline. The pipeline begins with preprocessing the kinase structure and the fragment library. Subsequently, subpocket-based fragment docking and growing are carried out in two phases: core docking and fragment growing. In the core docking phase, fragments specific to a predefined core subpocket are placed, and their conformations are scored, allowing the identification of hit fragments that serve as starting points for recombination. In the fragment growing phase, additional fragments are iteratively appended to these candidates, positioned into predefined subpockets, and evaluated. This procedure explores the search space in a tree-like manner using a beam search (see Figure 7c), while restricting tree width to prevent exponential runtime. The sequence of subpocket traversal, and thus the choice of subpockets, is predefined by the user as a subpocket path.

**Figure 7:**
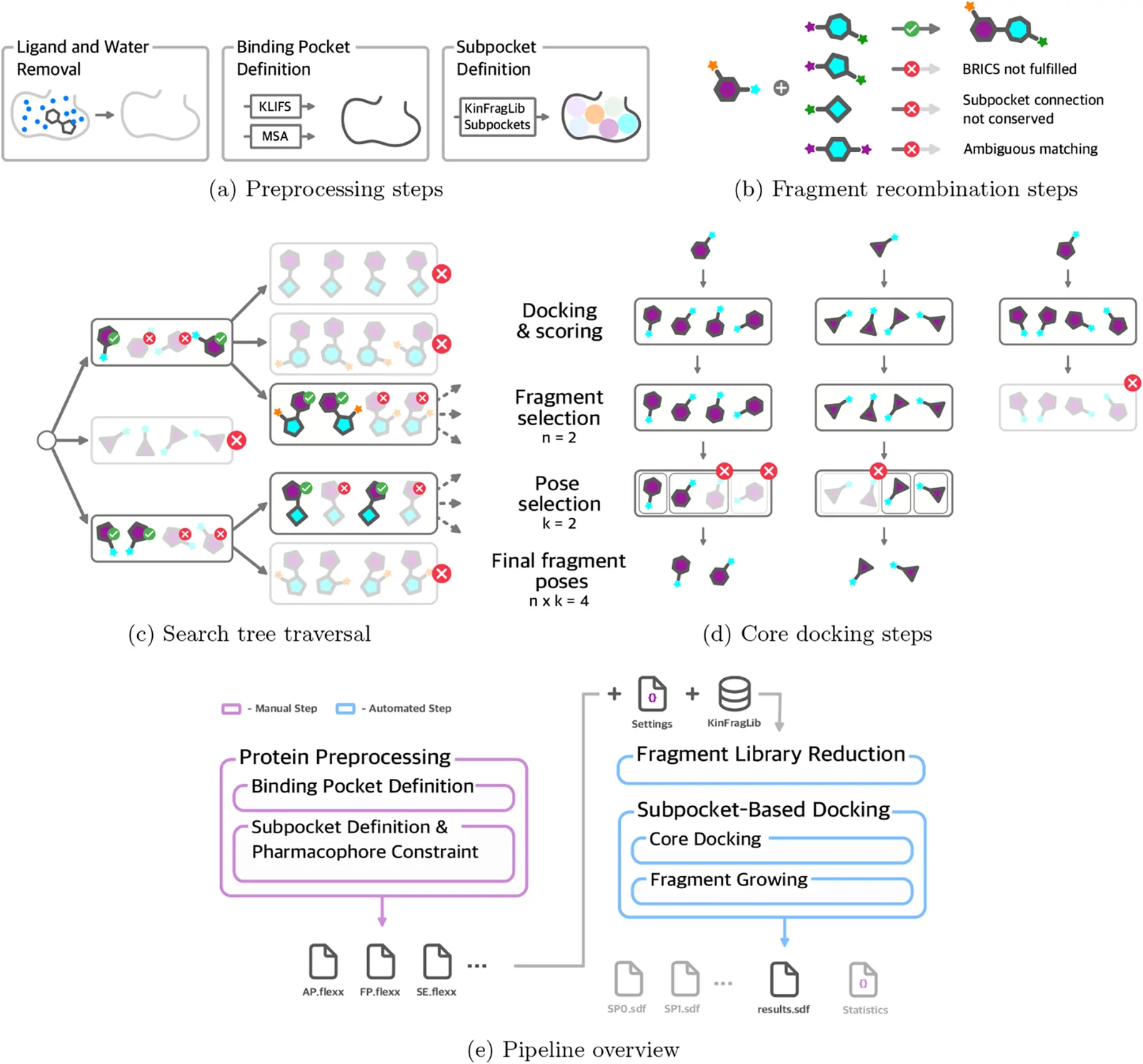
(a) Overview of protein structure preprocessing steps. First, water and small molecules are removed, then the binding pocket is determined—either directly inferred from KLIFS,^10^ or determined through a multiple sequence alignment (MSA) of KLIFS-defined binding pocket sequences. Lastly, the subpockets are defined through KinFragLib’s^26^ subpocket-specific anchor residues. (b) Recombining step. In this example, the core fragment (purple) should be expanded into the next subpocket (cyan)—i.e., merged with fragments assigned to the next subpocket. (c) Illustration of a search tree that is traversed/built during the core docking (first layer) of three fragments and the first fragment growing iteration (second layer). The nodes correspond to candidates with the respective docking poses. In this example, in each layer, *n* = 2 candidates are selected. Per candidate, *k* = 2 docking poses are chosen. (d) The three main steps in the core docking pipeline are docking and scoring, followed by the selection of *n* = 2 fragments based on the docking score and diversity. Finally, the poses are clustered based on RMSD similarity, and *k* = 2 poses are selected based on the docking score. (e) Overview of the subpocket-based docking pipeline.

While the protein preprocessing step requires a few manual steps, all subsequent steps are implemented in a fully automated pipeline. In the following, we describe the subpocket-based fragment docking methodology, i.e., protein structure preprocessing, core docking, and fragment growing, followed by outlining the technical details of the pipeline.

#### Protein Structure Preprocessing

Our subpocket-based docking pipeline can be applied to the orthosteric pocket (i.e., ATP-binding site) of any protein kinase structure of interest with a DFG-in conformation to search for novel inhibitor candidates. As a case study, we apply our pipeline to the PKA kinase from Cricetulus griseus (Chinese hamster). From 119 potential cocrystallized hamster PKA structures, we selected the structure 5N1F based on the quality of the structure (X-ray resolution: 1.12 Å). To prepare the structure for the subpocket-based docking procedure, we (1) remove waters that are not conserved and other small molecules (MDP molecules in 5N1F), (2) define the binding pocket based on the 85 KLIFS-defined binding pocket residues,^10^ and (3) specify the subpockets (i.e., subpocket path and pharmacophore constraints) (see Figure 7a). For a different kinase, we recommend to choose the subpockets to be traversed based on the respective binding site, either from prior knowledge or based on the cocrystallized ligand in the chosen crystal structure. For more details on the structure preprocessing, we refer to Supplementary Texts S2–S5.

#### Core Docking

As the first step of our structure-informed docking pipeline, we perform docking of fragments in the user-defined core subpocket. All fragments from the KinFragLib fragment pool assigned to this subpocket are placed into this core subpocket. Well-positioned, and thus high-scoring, fragments serve in the following steps as a starting fragment for template docking to grow into other subpockets. A core subpocket pharmacophore constraint ensures correct fragment placement during core docking.

#### Core Docking and Scoring

The user-defined FlexX definition file holds all information to instruct the docking process. It contains information on the crystal structure of interest, the binding pocket, as well as the pharmacophore constraint that surrogates the subpockets. The standard FlexX docking procedure, including the pharmacophore constraints and the chosen docking parameters, is evoked, and each fragment is placed and scored. Next, HYDE is called to rescore the docked poses. Note that HYDE optimization allows adapting the location of the docked pose to optimize the score. To keep the compounds in the defined subpockets for our application, we exclude poses that have been moved beyond a user-defined displacement threshold. By default, we permit a displacement of up to 2.0 Å, in line with the GUI version of SeeSAR.^36^ After conducting HYDE scoring and optimization, we utilize the estimated affinity (HYDE score) instead of the docking score as a quality measure for the docked poses, which we will refer to as the fragment score.

#### Fragment and Pose Selection

To prevent combinatorial explosion, we select a diverse subset of *n* promising core fragments as candidates for further growth (see Figure 7d). We aim to balance selecting promising, high-scoring fragments with maintaining diversity, avoiding a focus on very similar fragments that could trap the search in a local minimum. Thus, we follow a cluster-based strategy and adopt a stochastic hill climbing approach.^49^ We first cluster the fragments based on Tanimoto similarity between the RDKit’s topological fingerprints^45^ of the molecules using Butina Clustering.^50^ Next, to rank clusters based on the scores of their fragments, we determine a cumulative score for each cluster. Note, to reduce computational runtime, we ignore that each fragment can have several docking poses in this step and use the fragment score of the best-scoring pose per fragment. The cluster score *c*_*i*_ of cluster *i* (see eq 1) is the average score of its fragments after excluding the lowest 25% of fragments, which form the third quantile *Q*_*i*;3_.

1 $$c_{i}=\frac{1}{\left\lceil 0.75\times |C_{i}|\right\rceil }\underset{f\in C_{i}\backslash Q_{i;3}}{\sum }\mathrm{score}\left(f\right)$$

Ignoring these low-scoring fragments makes the score more robust to outliers. High-scoring fragments are not excluded, since the best-scoring compounds from each cluster will be selected later. Next, we remove the 10% lowest-scoring clusters to eliminate unfavorable clusters and normalize the remaining cluster scores using min-max scaling. We then iteratively *randomly* sample clusters based on their normalized scores, ensuring that more promising clusters are selected more frequently while maintaining diversity. To achieve this, we transform the score of a cluster *i* using a *softmin* function (see eq 2) where *S* is the set of all scaled cluster scores and *p* (by default *p* = 1) is a hyperparameter that weights the influence of the scores on the sampling probability.

2 $$\mathrm{softmin}\left(s_{i}\right)=\frac{\frac{e^{-s_{i}}}{p}}{\underset{s_{j}\in S}{\sum }\frac{e^{-s_{j}}}{p}}$$

For each drawn cluster, we select the best-scoring fragment and remove it from the cluster. We repeat this *n* times to select *n* fragments, updating the distribution after each iteration to reflect the new cluster scores. For a less diverse, more focused selection, fragments can alternatively be chosen solely based on their individual scores (top *n* fragments), which may be useful for concentrated searches in smaller fragment libraries.

##### Pose Selection

For the chosen subset of fragments, we next consider and filter their docking poses. Since it is challenging to identify the single perfect conformation of a fragment, most docking programs generate several docking poses ranked by docking score. We obtain up to 10 poses per docking run. Some poses may be very similar or in unfavorable positions. Therefore, we cluster all poses per fragment to group similar poses and only select the highest-scoring pose per cluster. We then choose the top *k* (with *k* ≤ 10) poses, so unfavorable poses can be excluded. These selected poses serve as templates during fragment growing. We utilize Butina^50^ for clustering, using RMSD as the similarity metric. After visual inspection of different poses for several fragments, we set the clustering threshold to an RMSD of 1.5 Å. From each cluster, we choose the pose with the best fragment score. We keep all selected poses if the number of clusters is ≤ *k*; otherwise, we retain only the top *k* selected poses per fragment in the final step to avoid polynomial runtime (see Figure 7d).

#### Fragment Growing in the Binding Site

Next, core fragments are expanded in the binding pocket following a user-defined subpocket path, a sequential list that dictates the order of subpockets for fragment growth. Since fragments are only placed into their assigned subpocket according to the KinFragLib methodology, this path determines which fragment pool is used. Iteration through the list performs one growth step per subpocket.

For a given subpocket *sp* and a set of already docked fragments (candidates), each iteration begins by recombining every candidate with all fragments from the library assigned to *sp*. The resulting recombinations are then docked using the docking poses of the candidate fragments as templates, optionally followed by scoring and optimization. Finally, a subset of the docked fragments and poses is selected to serve as candidates and templates for the next subpocket iteration. Each step of a fragment growing iteration for a given subpocket *sp* is described in detail below.

#### Recombination

Within the recombination step, we combine the candidates from previous iterations with the fragments assigned to the given subpocket *sp*. Hence, a candidate can already consist of several KinFragLib fragments. For each candidate, we attach the new fragment if they are compatible, as illustrated in Figure 7b. If multiple fragments within the candidate are compatible with the new fragment, we generate one recombination per possibility to avoid introducing rings. Two fragments are compatible if they fulfill two constraints: (1) the original subpocket connection is preserved, and (2) they follow the BRICS rules.^48^

1 First, fragments are only recombined if each has a connection to the subpocket of the other fragment. That is, at least one dummy atom of the first fragment is annotated with the subpocket of the second fragment and *vice versa*. These subpocket annotations are an integral feature of KinFragLib,^26^ where each dummy atom represents a connection to another fragment in the original ligand.
2 Each KinFragLib fragment is annotated with an environment corresponding to the BRICS fragmentation rules.^26,48^ Fragments are only recombined if their environments are compatible, ensuring synthetic accessibility and increasing the likelihood of generating synthetic feasibility ligands.

To combine two compatible fragments, we introduce a single bond between the adjacent atoms of the corresponding dummy atoms. In a few cases, fragments have more than one dummy atom labeled with the same subpocket connection (see the last example in Figure 7b). Since we cannot find an unambiguous matching of dummy atoms to form a bond connecting two fragments, we exclude these cases.

#### Docking and Scoring

After each recombination, an energetically favorable 3D structure of the constructed molecules is generated using RDKit functionality.^51^ The recombined molecule is then placed into the binding pocket through template docking, using the previously selected docking poses of the candidate as templates. This ensures that the original fragments remain in their assigned subpockets while only the new fragment is positioned. For each candidate pose and each recombination, FlexX^27^ determines the maximum common substructure (MCS) between the template and the recombination, generating slightly varied superpositions to allow flexibility. To guide the placement of the new fragment into its subpocket *sp*, a subpocket-specific pharmacophore constraint is applied, ensuring the recombination docks while maintaining the template pose. Optionally, HYDE scoring and optimization are performed on the docked molecules, similar to core docking. Poses that exceed a displacement threshold during optimization are discarded, as the original fragments may no longer occupy their respective subpockets.

#### Fragment and Poses Selection

At the end of each subpocket iteration, we select *n* fragments and up to *k* poses per fragment to serve as candidates for recombination and docking templates for the next iteration. The selection procedure follows the same methodology as in the core docking step.

### Pipeline

The automated subpocket-based docking pipeline is depicted in Figure 7e. First, the FlexX docking^27^ and HYDE scoring^30^ tasks are configured using the SeeSAR GUI.^36^ This configuration includes the kinase structure, selected binding pocket residues, and subpocket definitions encoded as pharmacophore constraints (see Supplementary Texts S4–S5). FlexX- and HYDE-specific configuration files are generated for each subpocket of interest. This step is performed manually once prior to running the automated subpocket-based docking program itself.

With the configuration files prepared, the subpocket-based docking is executed in a fully automated pipeline comprising three main components: (1) fragment library preparation, (2) core docking, and (3) iterative fragment growing (see Figure 7e). For library preparation, the KinFragLib fragment library^26^ is loaded and preprocessed according to user-defined filters using CustomKinFragLib.^38^ Core docking and iterative fragment growing are then performed.

The pipeline is fully customizable to accommodate different projects and computational resources. Users can specify the core subpocket, define the subpocket path for fragment growing, select fragment and docking pose strategies, and adjust fragment library filters and their parameters. Detailed instructions and an example configuration file are provided in the repository (https://github.com/volkamerlab/KinFragLib_PocketEnum/blob/main/config/5n1f/settings.json). The pipeline outputs the generated compounds in SDF format and program statistics in JSON format.

### Experimental Methods

#### Protein Expression and Purification

A sample of *E. coli* BL21­(DE3) gold cells with a transformed plasmid containing the DNA-sequence of CHO-PKA in a modified pET16b-vector was kindly provided by S. Dörr, Philipps University Marburg.^52^ Expression and purification were performed according to a slightly modified version of the procedure previously described by B. Wienen-Schmidt, Philipps University of Marburg.^52^ Expression was carried out in SLB media overnight at 291.15 K. Cells were harvested by centrifugation and disrupted using an ultrasonic homogenizer. The lysate was separated by centrifugation (1.5 h 20000 g). The supernatant was then purified using an Äkta Pure FPLC system at 277.15 K. For affinity chromatography, the protein was loaded onto a Ni-NTA column (HisTrap FF 5 mL, Cytiva), to which the 7xHis-tag of CHO PKA binds. Elution was controlled using a rising imidazole gradient (10 mM to 500 mM). The eluted protein was treated with TEV protease for 48 h to cleave the 7xHis-tag, followed by affinity chromatography again. CHO-PKA was no longer able to bind to the column and was thus collected in the flow through. After renewed dialysis onto a HEPES buffer with a low concentration of NaCl (2.5 mM), the phosphorylation levels of CHO-PKA were separated by cation exchange chromatography (Mono S, Cytiva) with increasing NaCl gradient (2.5 mM to 500 mM).^52^

#### Affinity Determination

The residual kinase activity was determined in accordance with the vendor’s protocol^53^ in white 96-well plates (Greiner, Kremsmünster, Austria) using a Tecan Safire2 (Tecan, Männedorf, Switzerland) and a KinaseGlo Luminescent Kinase Assay Kit (Promega, Walldorf, Germany) at 298.15 K. Each well contained 3.25 μL of PKA in buffer (40 mM Tris pH 7.5, 20 mM MgCl_2_, 0.1 mg/mL BSA; stock 9.74 nM, 1.22 nM in assay), 3.25 μL of substrate Kemptide (Promega, Walldorf, Germany) dissolved in buffer (stock 518.13 μM, 64.77 μM in assay) and 7.5 μL of inhibitor dissolved in buffer containing additional 4% (v/v) DMSO (stock 20 mM, 10/50/100 μM in assay). The addition of 15 μL of ATP dissolved in buffer (20 μM stock, 10 μM in assay) started the incubation time of 1 h. Each luminescence measurement was started 10 min after the addition of 30 μL KinaseGlo-reagent dissolved in buffer. The residual kinase activity (%) values were obtained in individual triplicates and calculated in relation to the maximum kinase activity (no addition of inhibitor) and no kinase activity (no addition of Kemptide).

#### IC_50_ Determination

Compounds were tested by a third-party vendor (Eurofins Inc.) using a radiometric assay.

### Synthesis

We describe the synthesis steps for all compounds with promising activity here (<50% residual kinase activity at 100 μM compound concentration). The synthesis for all less active compounds are included in the Supplementary Information.

#### Compound **30**

As described by Jackson et al.,^54^ the reaction of 1-bromo-3-(bromomethyl)­benzene **27** with 1*H*-1,2,4-triazole **28** in a Finkelstein reaction leads to the intermediate **29**. The subsequent Suzuki coupling with the corresponding boronic acid pinacol ester according to Kwiatkowski et al.^55^ yields the target compound **30** in only two steps in good overall yield (see Figure 8a).

**Figure 8:**
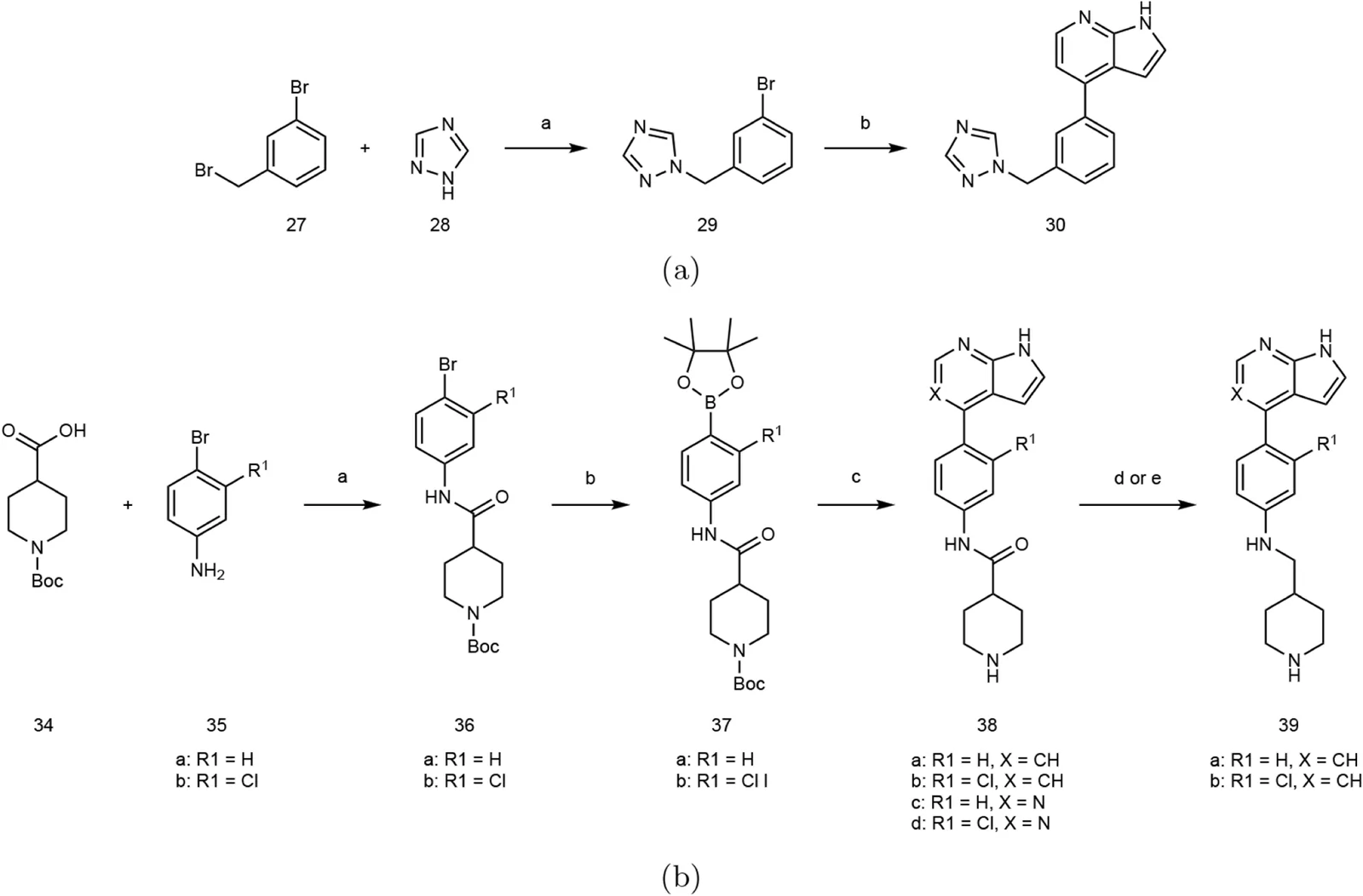
(a) Synthesis of **30**. Reagents and conditions: a) KI, K_2_CO_3_, acetone, 55 °C, 16 h, 68%; b) 4-(4,4,5,5-tetramethyl-1,3,2-dioxaborolan-2-yl)-1*H*-pyrrolo­[2,3-*b*]­pyridine, Pd­(dppf)­Cl_2_ DCM adduct, K_3_PO_4_, 1,4-dioxane/H_2_O (4:1), 110^◦^C, 18 h, 68%. (b) Synthesis of **39a**, **39b**, **38a**, **38b**, and the precursors **38c** and **38d**. Reagents and conditions: a) 1-ethyl-3-(3-(dimethylamino)­propyl)­carbodiimide hydrochloride, DMAP, DCM, 0^◦^C, rt, 16 h, for **36a**: 82%, for **36b**: 91%; b) *bis*(pinacolato)­diborane, KOAc, Pd­(dppf)­Cl_2_ DCM adduct, 1,4-dioxane, 100^◦^C, 3 h, for **37a**: 68%, for **37b**: 96%; c) 4-bromo-7-azaindole, Na_2_CO_3_, Pd­(PPh_3_)_4_, DME/H_2_O (2:1), MW (300 W), 130^◦^C, 1 h, 4 M HCl in 1,4-dioxane, rt, 24 h, for 38a*2 HCl: 74%, for **38b**: 79%, using 4-bromo-7*H*-pyrrolo­[2,3-*d*]­pyrimidine instead of 4-bromo-7-azaindole, for 38c*HCl: 54%, for 38d*HCl: 61%; d) for 39a*formate: LAH 2.4 M in THF, THF, 0^◦^C, rfx, 16 h, 37%; e) for 39b*formate: bis­(2-methoxyethoxy)aluminum dihydride (Red-Al) 3.5 M in toluene, THF, 0^◦^C, rfx, 16 h, 43%.

#### Compounds **38**, **39**, and Derivatives

The coupling of *N*-Boc-protected piperidine-4-carboxylic acid **34** with either 4-bromoaniline **35a** or 4-bromo-3-chloroaniline **35b** furnished the respective carboxamides **36a** and **36b**, which were subsequently converted into the corresponding boronic acid pinacol esters **37a** and **37b**. Suzuki coupling of the latter using 4-bromo-7*H*-pyrrolo­[2,3-*d*]­pyrimidine yielded **38a** and **38b**, while usage of 4-bromo-7-azaindole gave **38c** and **38d**. Finally, in the case of **39a**, the carboxamide was reduced using LAH. Since we observed partial dehalogenation during the reduction of **39b** with LAH, we used sodium bis­(2-methoxyethoxy)aluminum dihydride (Red-Al) instead to avoid dehalogenation in the final step (see Figure 8b).

Unless otherwise stated, commercially available solvents and reagents were used without further purification. Reactions that are sensitive to moisture or oxygen were carried out in heated glass apparatus under an inert gas atmosphere (Argon N50, Air Liquide). In the case of reactions sensitive to moisture, commercially available anhydrous solvents were used. The reactions using microwave radiation were carried out in sealed pressure vessels in a Discover system with Explorer autosampler from CEM. Reaction control was performed by thin-layer chromatography using Merck 60 F_254_ silica plates and analyzed under UV light or by staining. In some cases, an HPLC-MS system consisting of an Agilent 1260 Infinity system with a Poroshell column (120, EC-C18, 2.7 μM, 4.6 mm × 50 mm, Agilent) and an Expression S CMS from Advion was used for reaction control. Purification was generally performed by MPLC using three different devices, a Reveleris X2 (Büchi) system, a Pure C-850 FlashPrep (Büchi) system or a Puriflash 5.020 (Interchim) system. Prepacked cartridges of different particle sizes and loading capacities from different manufacturers were used (FlashPure from Büchi, PuriFlash from Interchim). Purification by HPLC was performed on a Pure C-850 FlashPrep system from Büchi with a PrepPure column (C18, 100 Å, 10 μM, 250 mm × 20 mm, Büchi). The reaction products were dried under reduced pressure (10^–3^ mbar) and in some cases at an elevated temperature (50^◦^C) for several hours to days. The NMR spectra were recorded in the NMR facilities of the chemical or pharmaceutical department on an AV III 300 MHz (Bruker), an ECZ400S or ECA500 (JEOL) instrument, and processed and analyzed with the Delta software (JEOL). NMR spectra were recorded in DMSO-*d*_6_ or MeOH-*d*_4_ and the indicated chemical shifts (in ppm) were calibrated to the signal of the residual proton content of the solvents (DMSO-*d*_6_: 2.50/39.52; MeOH-*d*_4_: 3.31/49.00).^56^ Spin multiplicities are stated as follows: s = singlet, brs = broad singlet, d = doublet, t = triplet, q = quartet, dd = doublet of doublet, ddd = doublet of doublet of doublet, tt = triplet of triplet, m = multiplet, sm = symmetrical multiplet. HRMS spectra were recorded at the analytical department for mass spectrometry and elemental analysis of the chemistry department on an LTQ-FT Ultra system (Thermo Fisher Scientific). Melting points were determined using a Krüss M 5000 device and are uncorrected. The purity of all synthesized compounds was determined by elemental analysis, qNMR, or HPLC. Full experimental details for all compounds, as well as intermediates, can be found in the Supplementary Information.

#### Protein Crystallography

##### Crystallization

Microseed matrix screening was performed on a sitting drop 24-well crystallization plate at 277 K. First 10 mg/mL PKA (240 μM) was incubated with crystallization buffer containing 30 mM MBT (MES/Bis-Tris buffer, pH 6.9), 1 mM DTT, 0.1 mM EDTA, 75 mM LiCl and 0.03 mM Mega 8, 0.07 mM PKI (Sigma, P7739) and 1.2 mM ligand dissolved in DMSO from a 100 mM stock.^52^ 1.5 μL of this protein solution was pipetted into the plate. The reservoir contained a mixture of 23% methanol in water. 1 μL of reservoir solution was added to the protein solution. To obtain crystals with a higher resolution, 0.5 μL of a seeding solution was added to the solution mix in the plateau.^57^ Seeding solution was obtained by crushing existing PKA crystals and preparing them according to the protocol from Douglas Instruments.^58^ To obtain crystals, vapor diffusion was then carried out at 277 K. After 2 weeks, the crystals had grown large enough to be collected. For crystal mounting, crystals were incubated in different buffers containing 30 mM MBT (MES/Bis-Tris buffer, pH 6.9), 1 mM DTT, 0.1 mM EDTA, 75 mM LiCl, 0.03 mM Mega 8, 0.07 mM PKI (Sigma, P7739), 1.2 mM ligand dissolved in DMSO from a 100 mM stock and 10–30% MPD. To ensure the best possible resolution, the crystal was incubated for 1 min each in 10%, 20%, 25%, and 30% MPD solutions and finally flashfrozen in liquid nitrogen.^52^

##### Data Collection, Processing, Phasing and Refinement

The data set was collected at beamline P13 from the electron storage ring DESY Hamburg, Germany on an EIGER 16M pixel detector with a resolution of 1.38 Å. The data set was processed using XDSAPP 3 and converted into the MTZ format using XDSCONV.^59^ Molecular replacement was then performed with Phaser MR^60^ and a modified PDB structure of PKA from cricetulus griseus (PDB code: 5N1F)^61^ in which all ligands, hydrogens, and water atoms have been removed. This was followed by multiple refinement cycles of positional coordinate, occupancy, and B-factor refinement using Phenix.^62^ Protein was anisotropically and water atoms isotropically refined. Coot^63^ was used for model building with residue fitting, as well as placing water atoms and ligands with the use of σ-weighted 2F_o_ – F_c_ and F_o_ – F_c_ electron density maps. If the electron density showed an alternative conformation, incorporation was done if the less populated form had an occupancy of at least 30%. Hydrogen atoms have been added again to the final model with Phenix.reduce.^64^ For structure validation, ramachandran plots were generated using PROCHECK^65^ and temperature factors were analyzed with Moleman.^66^ For Rfree determination, 5% of all reflections were randomly chosen and not used for the refinement. The required restraints and corresponding coordinates for the ligand were created using the grade Web server.^67^ Structural figures were prepared with PyMol.^68^ The refinement statistics can be found in Supplementary Table S2.

## Supplementary Material



## Data Availability

**Data and Software Availability:** The subpocket-based docking pipeline is implemented in Python and is available on GitHub (https://github.com/volkamerlab/KinFragLib_PocketEnum). The repository includes a Conda environment file specifying all required packages. KinFragLib (https://github.com/volkamerlab/KinFragLib)^26^ must be installed and the fragment library downloaded. Alternatively, a Docker^69^ image is provided to run the pipeline in a containerized environment. FlexX^27^ and HYDE^30^ must be installed with valid licenses to execute the pipeline. RDKit^51^ is used frequently throughout the pipeline. Pipeline progress can optionally be monitored using Weights & Biases.^70^ ChEMBL33 ^33^ was used and is available at 10.6019/CHEMBL.database.33. The generated data resulting from the case study on the hamster PKA are accessible on Zenodo (https://zenodo.org/records/17628467, DOI: 10.5281/zenodo.17628467). Experimental protocols and assay measurements are available within the paper and its Supplementary Files. The crystallization data have been deposited in the PDB^71^ with the accession code “9TEC” and will be accessible upon publication.

## Notes

Additional experimental details and methods, including synthesis schemes and ^1^H NMR spectra for all compounds; diversity and novelty analysis of generated compounds; protein preprocessing and program parameters for the case study (PDF)
