# Supplementary material for: Subpocket-Aware Fragment-Based Docking Pipeline for Kinase Ligand Design: Methodology and a PKA Case Study
Source: J Chem Inf Model. 2026 Oct 15;66(20):13397–412. doi: 10.1021/acs.jcim.6c01373 (PMC13715515; doi:10.1021/acs.jcim.6c01373)
Supplement: Supplementary file 1 [file ci6c01373_si_001.pdf]

Subpocket-Aware Fragment-Based Docking Pipeline for Kinase Ligand Design: Methodology and PKA Case Study

Katharina Buchthal[†*], Paula Linh Kramer[†*], Dominik Hubach[‡], Gina Bach[‡], Naemi Wagner[‡], Johannes Krieger[‡], Moritz Klöpper[‡], Michael Daude[‡], Wibke E. Diederich[‡§], and Andrea Volkamer[†§]

†Data Driven Drug Design, Center for Bioinformatics, Saarland University, Campus E2.1, Saarbrücken 66123, Germany

‡Philipps-Universität Marburg, Fachbereich Pharmazie, Institut für Pharmazeutische Chemie und Zentrum für Tumor und Immunbiologie, Hans-Meerwein-Straße 3, Marburg 35043, Germany

*These authors contributed equally to this work.

§ Corresponding authors

Supplementary Information

Content

Table S1: Display of the corresponding difference electron density for DHS003 4

Table S2: Crystallographic table for the datasets of compound **30** 5

Table S3: CustomKinFragLib filter parameters 6

Table S4: Similar ChEMBL compounds 7

Table S5: Pharmacophore constraints for each subpocket 8

Table S6: Residual kinase activity measurements 8

Figure S1: Docking pose of L03700308 and **7** 9

Figure S2: Docking pose of L00710372 9

Figure S3: Diversity and novelty analysis 10

Figure S4: Most similar PKA compound with the corresponding generated compound 11

Figure S5: Correlation of assay measurements and HYDE estimated affinity 12

Text S1: Overlapping binding mode of crystal ligand in AKT2 and docked ligand in PKA 13

Text S2: SeeSAR docking engine 13

Text S3: Preserved waters 13

Text S4: Binding pocket definition & identification 14

Text S5: Subpocket and pharmacophore definition 14

Text S6: Shannon entropy as a molecular diversity measure 14

Text S7: Ligand diversity evaluation 15

Text S8: ChEMBL database comparison 15

Text S9: Drug-likeness analysis of generated compounds.. 16
Text S10: Program settings for case study on PKA 16
Scheme S1: Synthesis of **7**, **8**, **9**[a] 17
Scheme S2: Synthesis of **17**[a] 18
Scheme S3: Synthesis of **20**[a] 18
Scheme S4: Synthesis of **24** [a] 19
Scheme S5: Synthesis of **26** [a] 19
Scheme S6: Synthesis of **33**[a] 20
N-(5-Bromo-2-fluorophenyl)-2-methylpropane-2-sulfinamide (**2**) 20
N-(5-Bromo-2-fluorophenyl)-2-methylpropane-2-sulfonamide (**3**) 20
4-Bromobenzo[b]thiophene-2-carboxamide (**5**) 21
4-(4,4,5,5-Tetramethyl-1,3,2-dioxaborolan-2-yl)benzo[b]thiophene-2-carboxamide (**6**) 21
4-{3-[(1,1-Dimethylethyl)sulfonamido]-4-fluorophenyl}benzo[b]thiophene-2-carboxamide (**7**)..... 22
N-[2-Fluoro-5-(1-oxo-1,2-dihydroisoquinolin-6-yl)phenyl]-2-methylpropane-2-sulfonamide (**8**).... 22
N-[2-Fluoro-5-(1*H*-indazol-5-yl)phenyl]-2-methylpropane-2-sulfonamide (**9**) 23
3-Methyl-1*H*-indazol-4-ol (**11**) 23
tert-Butyl 4-hydroxy-3-methyl-1*H*-indazole-1-carboxylate (**12**) 24
N-(3-Hydroxypropyl)thiophene-2-carboxamide (**15**) 25
tert-Butyl 3-methyl-4-[3-(thiophene-2-carboxamido)propoxy]-1*H*-indazole-1-carboxylate (**16**)..... 25
N-{3-[(3-methyl-1*H*-indazol-4-yl)oxy]propyl}thiophene-2-carboxamide (**17**)................................. 26
[4-(2-Chloropyrimidin-4-yl)phenyl]methanone (**19**)... 26
{4-[2-(Ethylamino)pyrimidin-4-yl]phenyl}(phenyl)methanone (**20**) 27
(2-Amino-6-bromophenyl)methanol (**22**) 27
N-(3-Bromo-2-{[(*tert*-butyldimethylsilyl)oxy]methyl}phenyl)benzamide **(23**)................................ 28
N-[3-(2-Aminopyridin-4-yl)-2-(hydroxymethyl)phenyl]benzamide (**24**) 28
{4-[7*H*-Pyrrolo(2,3-*d*)pyrimidin-4-yl]phenyl}(phenyl)methanone (**26**) 29
1-(3-Bromobenzyl)-1*H*-1,2,4-triazole (**29**) 29
4-{3-[(1*H*-1,2,4-triazol-1-yl)methyl]phenyl}-1*H*-pyrrolo[2,3-*b*]pyridine (**30**) 30
3-[(3-Morpholinopropyl)amino]benzamide (**33**) 30
tert-Butyl 4-[(4-bromophenyl)carbamoyl]piperidine-1-carboxylate (**36a**) 31
tert-Butyl-4-[(4-bromo-3-chlorophenyl)carbamoyl]piperidine-1-carboxylate (**36b**) 31
tert-Butyl 4-{[4-(4,4,5,5-tetramethyl-1,3,2-dioxaborolan-2-yl)phenyl]carbamoyl}piperidine-1-carboxylate (**37a**) 32
tert-Butyl-4-{[3-chloro-4-(4,4,5,5-tetramethyl-1,3,2-dioxaborolan-2-yl)phenyl]-carbamoyl}-piperidine-1-carboxylate (**37b**) 32
N-(4-(1*H*-Pyrrolo[2,3-*b*]pyridin-4-yl)phenyl)piperidine-4-carboxamide dihydrochloride (**38a**)....... 33
N-[3-Chloro-4-(1*H*-pyrrolo[2,3-*b*]pyridin-4-yl)phenyl]piperidine-4-carboxamide (**38b**).................. 34

N-[4-(7*H*-Pyrrolo[2,3-*d*]pyrimidin-4-yl)phenyl]piperidine-4-carboxamide Hydrochloride (**38c**) 34

N-[3-Chloro-4-(7*H*-pyrrolo[2,3-*d*]pyrimidin-4-yl)phenyl]piperidine-4-carboxamide Hydrochloride (**38d**) 35

N-(Piperidin-4-ylmethyl)-4-(1*H*-pyrrolo[2,3-b]pyridin-4-yl)aniline formate (**39a**) 36

3-Chloro-*N*-(piperidin-4-ylmethyl)-4-(1*H*-pyrrolo[2,3-b]pyridin-4-yl)aniline formate (**39b**) 37

References 38

Table S1: Display of the corresponding difference electron density for DHS003.

DHS003, at 3.0 σ [a]
9TEC
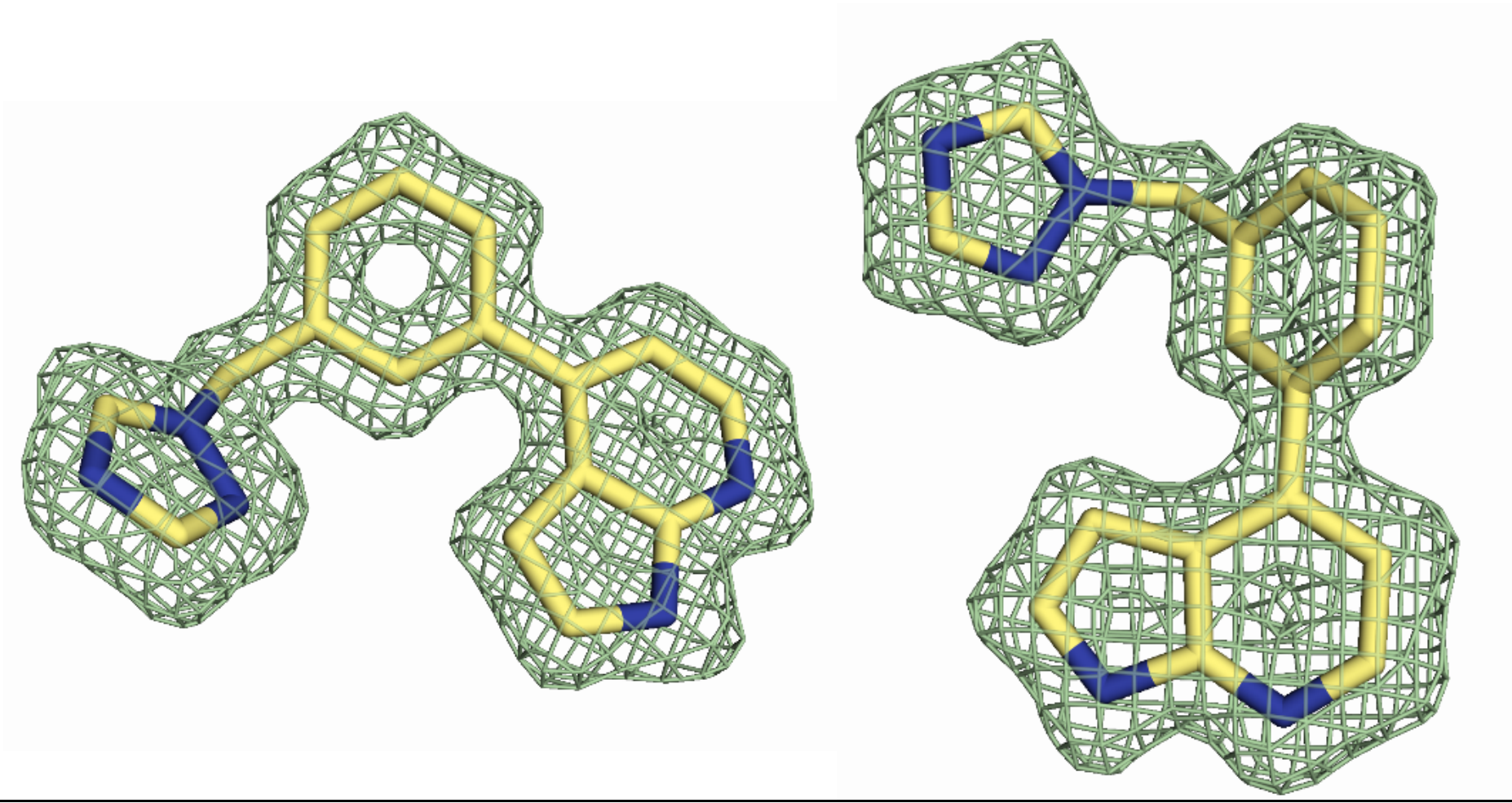

[a] all Atoms were 100% occupied, the ligand is shown from two different perspectives

Table S2: Crystallographic table for the datasets of compound **30**

**Compound**	**30**
PDB ID	9TEC
**(A) Data collection and processing**	
Beamline	PETRA III P13
No. Crystals used	1
Wavelenght λ [Å]	0.9763
Space group	$P2_12_12_1$
**Unit cell parameters**	
a/b/c [Å]	51.8 / 71.4 / 97.6
α/β/γ [°]	90.0 / 90.0 / 90.0
**(B) Diffraction Data**	
Resolution range [Å] [a]	48.79 - 1.38 (1.39 – 1.38)
Unique Reflections [a]	75034 (11943)
R(I)sym [%] [a,b]	8.2 (81.8)
Completeness [%] [a]	99.9 (99.6)
Redundancy [a]	13.1 (11.5)
I/σ [I] [a]	16.0 (2.0)
Wilson B-factor [$Å^2$]	16.3
Matthews Coefficient [$Å^3 \cdot Da^{-1}$] [c]	2.3
Solvent content [%] [c]	46.6
Molecules in asymmetric unit	1
**(C) Refinement with Phenix**	
PHENIX version	1.21.2-5419
Resolution range [Å]	29.40-1.38
Reflections used for R_{free}	3750
Reflections used for R_{work}	71257
**Final R values**	
R_{free} [%]	17.1
R_{work} [%]	14.4
**No. of atoms (*non-hydrogen*)** [d]	
Protein atoms	3114
Water molecules	296
Ligand atoms	21
RMSD form ideality	
Bond angles [°]	0.9
Bond length [Å]	0.007
**Ramachandran plot** [e]	
Most favoured regions [%]	92.4
Additionally allowed regions [%]	7.3
Generously allowed regions [%]	0.3
Outliers [%]	0
**Mittlere B-Faktoren [$Å^2$]** [d]	
Protein atoms	19.4
Water molecules	31.2
Ligand atoms	13.5

[a] Values in brackets are statistics for the highest resolution shell
[b] Rsym = $\Sigma|I-\bar{I}|/\Sigma I$; I is the observed intensity and $\bar{I}$ is the average intensity for multiple measurements
[c] calculated with CCP4 [1]
[d] calculated with MOLEMAN [2]
[e] calculated with PROCHECK [3]

Table S3: CustomKinFragLib filter parameters

List of all applied CustomKinFragLib [4] filters, and respective filter parameters. The column “Category” represents the target of the filters. The size of the fragment library after applying a filter is shown in the last column. The original KinFragLib size is shown in the first row.

**Category**	**Filter**	**Cutoff**	**Library size**
			9505
Redundancy	Prefilter	-	3505
Unwanted substructure	PAINS	-	3454
	Brenk	-	2767
Compound properties	Rule of three	-	2003
	QED	0.492	1482
Synthetic accessibility	SYBA	0.0	1363

Table S4: Similar ChEMBL compounds

Most similar ChEMBL compounds and their corresponding generated compound. The MCS is marked in red. All compounds with a similarity of ≥ 0.90 to a ChEMBL compound are listed. We include all targets against which the ChEMBL compound has been tested. However, negative measurements were excluded, as well as 0 % inhibition or IC50 measurements stated to be > 10 000 nM.

ChEMBL compound – generated compound	Tanimoto similarity	ChEMBL ID	Targets
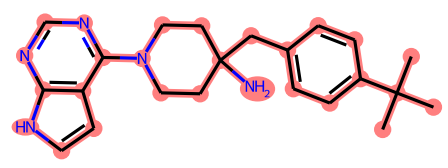 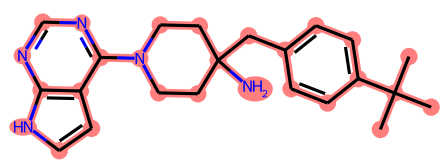	1.0	CHEMBL597211	Kinases: **PKA**, CHK1, CHK2, SRC, ROCK2, KDR, AKT2, RSK2, PKCalpha, SGK1, FLT3, ERK2, P70S6K, MAPKAPK2, CK2alpha, GSK3-beta<br>Other: CYP2C9
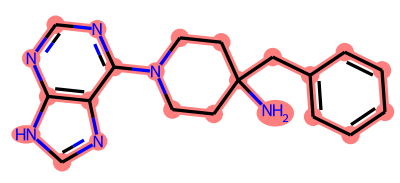 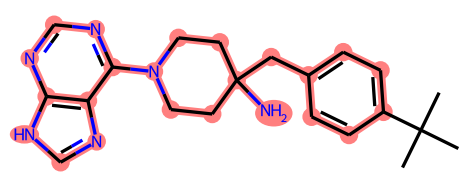	0.97	CHEMBL2420908	Kinases: **PKA**, LYN, ERK2, AURA, AKT1/2, CHK1/2, PKD2, FYN, GSK3delta, ABL, LCK, SRC, MSK1, RSK1, MAPKAPK5, ERK1, MET, CK1delta, MAPK14, MK2
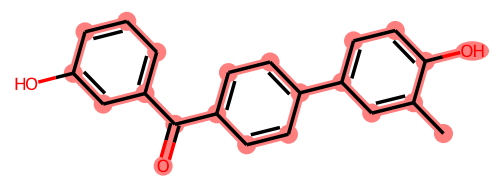 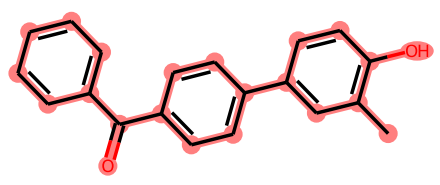	0.93	CHEMBL1928176	Other: cytosolic 17beta-HSD1, cytosolic 17beta-HSD2
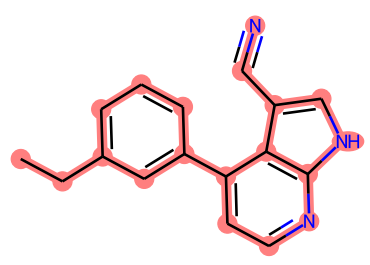 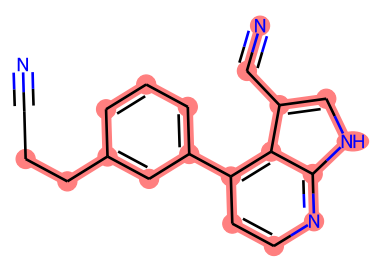	0.93	CHEMBL3959637	Kinases: LRRK2
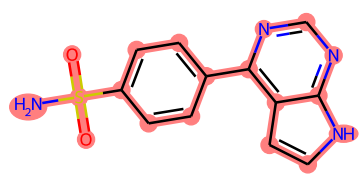 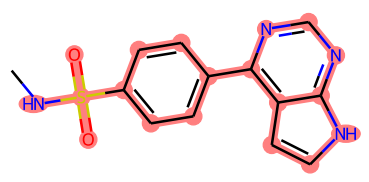	0.93	CHEMBL4068933	Kinases: IKK1, IKK2
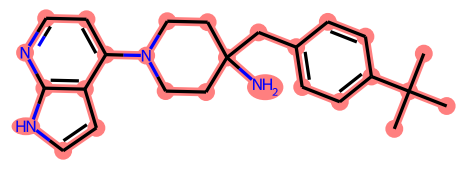 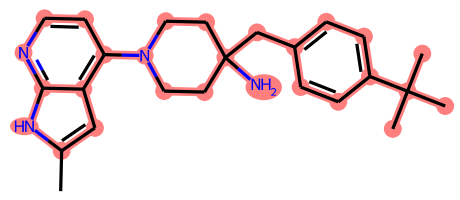	0.92	CHEMBL603262	Kinases: **PKA**, AKT2
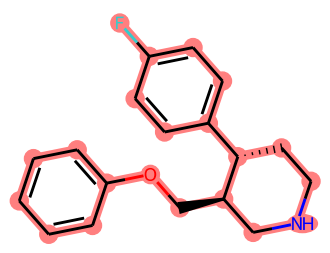 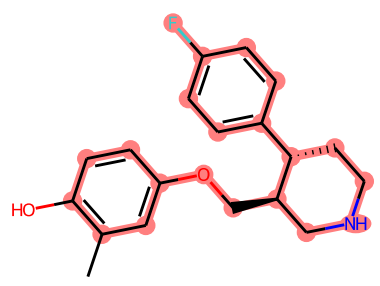	0.92	CHEMBL254489	Other: NorA-mediated efflux (tested in Staphylococcus aureus)
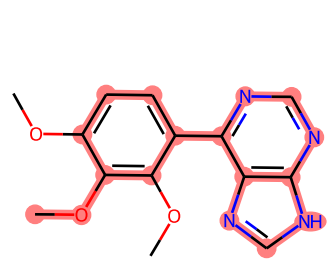 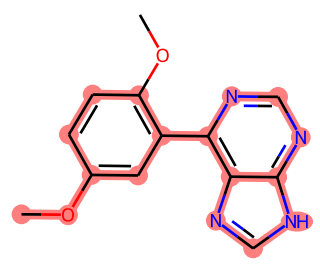	0.91	CHEMBL4974501	Other: Plasmodium falciparum organism (NF54 strain)
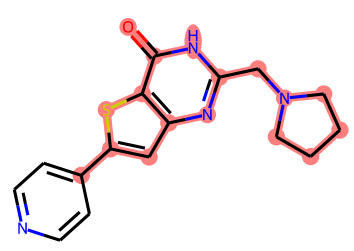 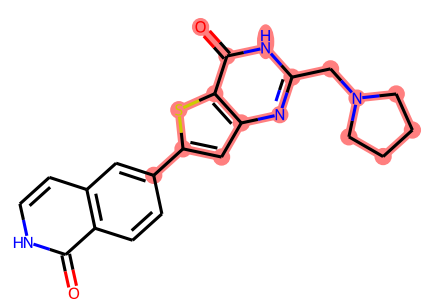	0.90	CHEMBL4080367	Kinases: CDC7 (in complex with ASK), ROCK1
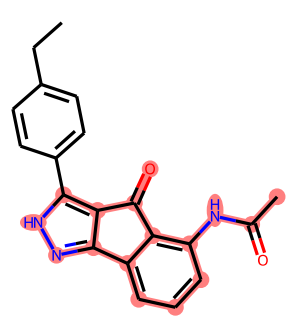 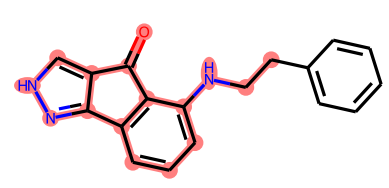	0.90	CHEMBL359180	Kinases: CDK4 (in complex with cyclin D1), CDK2 (in complex with cyclin E)

Table S5: Pharmacophore constraints for each subpocket

Subpocket	Anchor residues[a]	Radius	Constraint
AP	15, 46, 51, 75	3.5	Hydrogen bond donor or acceptor
FP	10, 51, 72, 81	4.5	Minimum of 3 heavy atoms
GA	17, 45, 81	4.5	[C,O,F,N,S,a]~[C,O,F,N,S,a]~[C,O,F,N,S,a]
SE	51	4.5	[C,O,F,N,S,a]~[C,O,F,N,S,a]~[C,O,F,N,S,a]
B1	28, 38, 43, 81	4.5	[C,O,F,N,S,a]~[C,O,F,N,S,a]~[C,O,F,N,S,a]
B2	18, 24, 70, 83	4.5	[C,O,F,N,S,a]~[C,O,F,N,S,a]~[C,O,F,N,S,a]

[a] Residue numbering follows the KLIFS [5] numbering scheme

Table S6: Residual kinase activity measurements

Residual kinase activity (%) of PKA for all synthesized ligands measured at 100 µM compound concentration. For all compounds with residual activity < 50% at 100 µM, measurements at 50 µM and 10 µM were performed. The residual kinase activity (%) values were obtained in individual triplicates and calculated in relation to the maximum kinase activity (no addition of inhibitor) and no kinase activity (no addition of substrate Kemptide).

ID	Mean $(100\ \mu M)$	Standard deviation $(100\ \mu M)$	Mean $(50\ \mu M)$	Standard deviation $(50\ \mu M)$	Mean $(10\ \mu M)$	Standard deviation $(10\ \mu M)$
30	5.95	3.74	9.56	3.61	14.18	2.25
38b	13.45	1.77	16.46	3.82	26.40	2.15
38a	10.63	2.09	13.63	1.82	22.21	2.88
39b	10.834	3.53	14.21	0.14	23.04	1.44
39a	9.50	1.94	12.40	1.60	15.01	1.94
38d	33.06	3.36	40.07	0.27	61.76	1.36
38c	19.43	4.47	23.30	2.79	33.54	1.25
7	90.74	5.71	-	-	-	-
8	52.77	1.85	-	-	-	-
9	74.18	7.31	-	-	-	-
24	87.38	1.60	-	-	-	-
17	54.80	5.36	-	-	-	-
33	92.75	2.72	-	-	-	-
20	94.00	2.38	-	-	-	-
26	50.82	1.82	-	-	-	-

Figure S1: Docking pose of L03700308 and **7**

Docking pose of the proposed molecule L03700308 (purple) and the adapted and synthesized molecule 7 (green) with the respective hydrogen bonds.

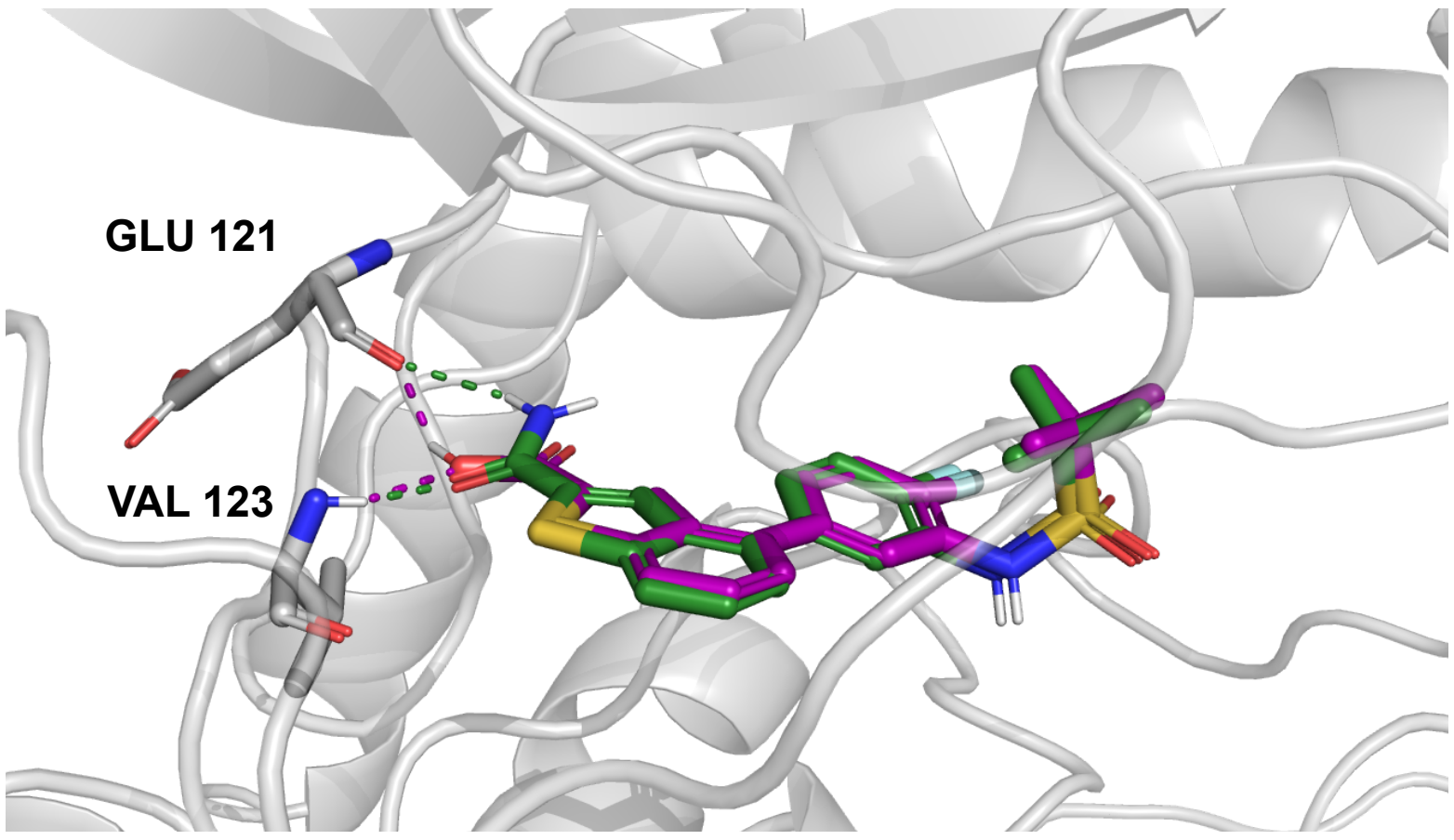

Figure S2: Docking pose of L00710372

Docking pose of molecule L00710372 highlighting the hydrogen bonds to glutamate 121 and valine 123. The ligand is colored in the respective subpocket colors (AP: purple, FP: green). Hydrogen bonds are indicated in green.

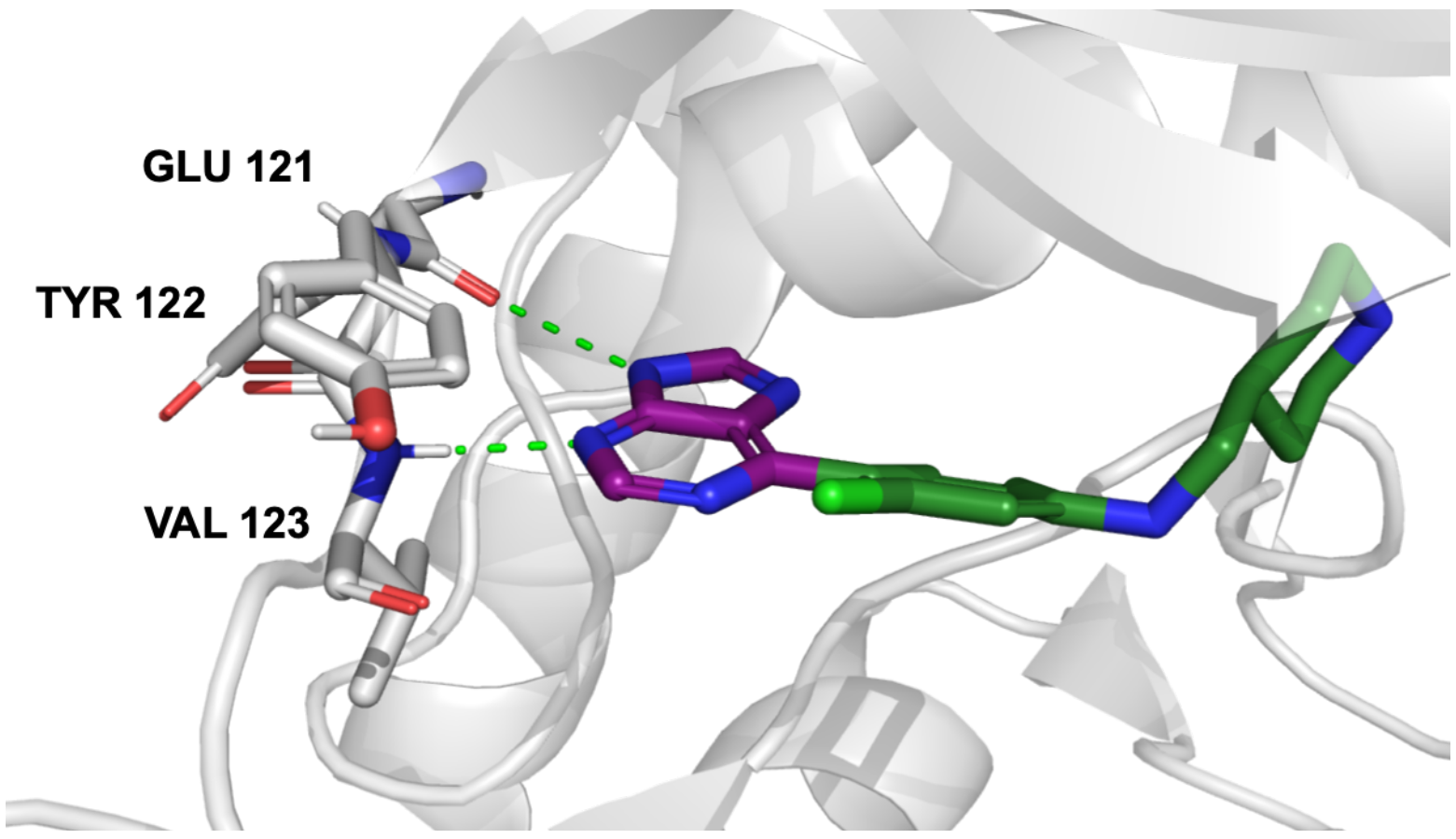

Figure S3: Diversity and novelty analysis

A: Top: Distribution of RDKit fingerprint similarity (Tanimoto coefficient) of post-filtered compounds to their nearest neighbor. Each bar represents the number of compounds falling within a similarity interval of width 0.05. Bottom: The corresponding boxplot with the Tanimoto similarity to the nearest neighbor. B: Distribution (top) and box plot (bottom) of Tanimoto similarities between post-filtered generated ligands and their respective most similar ChEMBL counterpart.

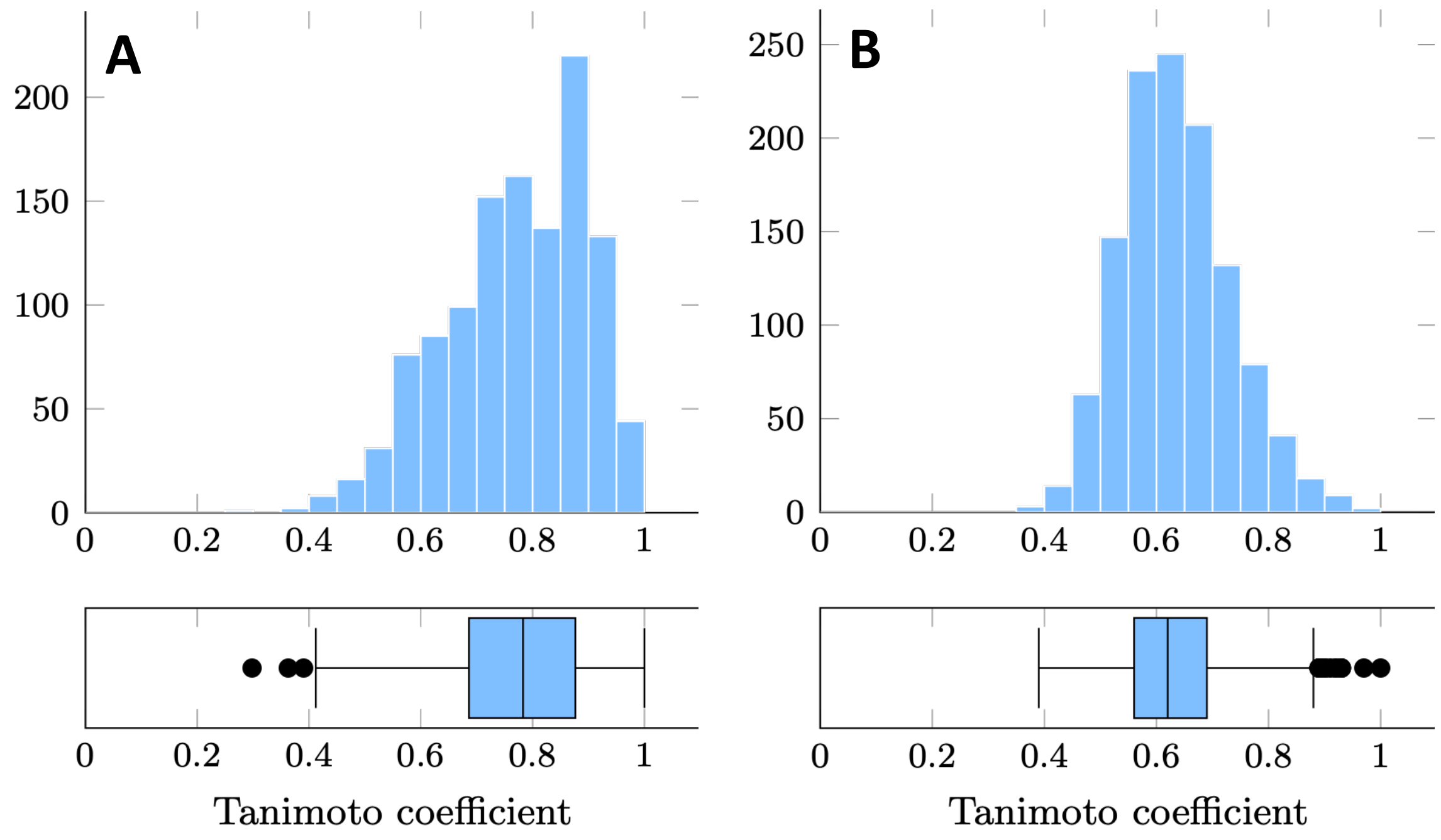

Figure S4: Most similar PKA compound with the corresponding generated compound

We investigated the novelty of our post-filtered ligands by comparing them to the cocrystallized ligands from the 119 available hamster PKA structures and 334 PKA structures (from all organisms) in the PDB (accessed November 2025). This revealed a mean similarity (Tanimoto coefficient using topological fingerprints) between the most similar PKA compounds and our compounds of 0.38±0.1 (among all PKA ligands), and 0.28±0.06 (hamster PKA). The most similar hamster PKA ligand (ligand code: 7WQ) compared to one of our compounds shows a Tanimoto coefficient of 0.64. While this ligand comprises the same AP fragment as our most similar compound, the remaining part exhibits significant structural differences. This indicates some degree of novelty among our generated compounds compared to existing PKA ligands.

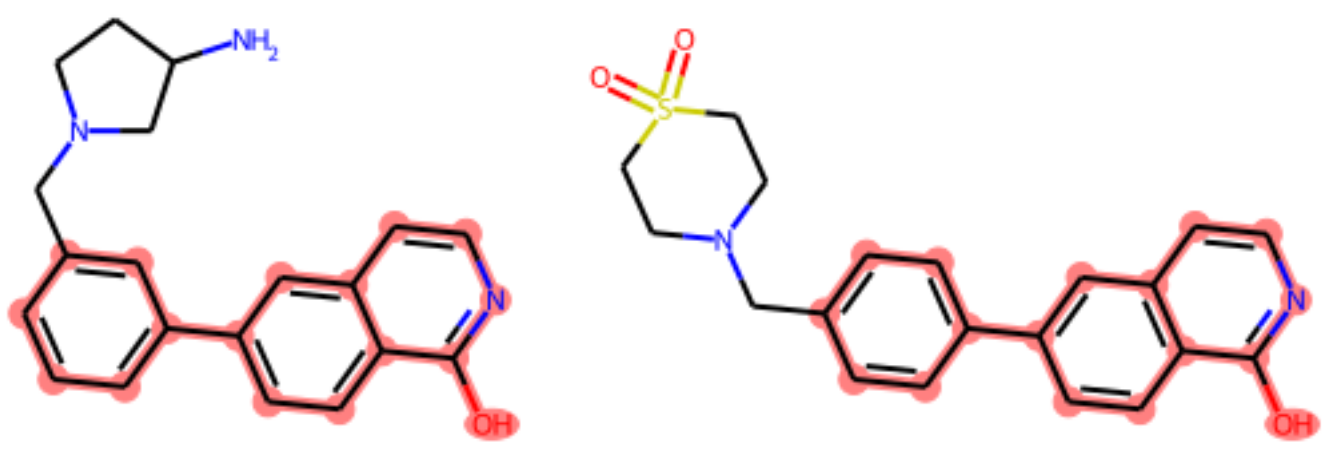

The most similar hamster PKA compound (7QW) and its corresponding generated compound, exhibiting a Tanimoto similarity of 0.64 using RDKit's topological fingerprints. The maximum common substructure (MCS) is marked in red.

Figure S5: Correlation of assay measurements and HYDE estimated affinity

A: Comparison of estimated affinity calculated by HYDE and kinase residual activity at 100 µM compound concentration for 15 synthesized compounds. The compounds were selected based on promising estimated affinity values and manual prioritization. The error bars indicate the standard deviation across three experimental replicates. The Spearman's rank correlation coefficient is $\rho = 0.146$, with a two-sided p-value of $p = 0.603$.

B: Comparison of estimated affinity calculated by HYDE and experimental IC_{50}. For this study, the five most promising compounds based on the measured kinase residual activity were selected. The Spearman's rank correlation coefficient is $\rho = -0.20$, with a two-sided p-value of $p = 0.747$.

Both analyses are exploratory and descriptive and are based on curated subsets of the tested compounds with limited sample sizes (n = 15 and n = 5, respectively).

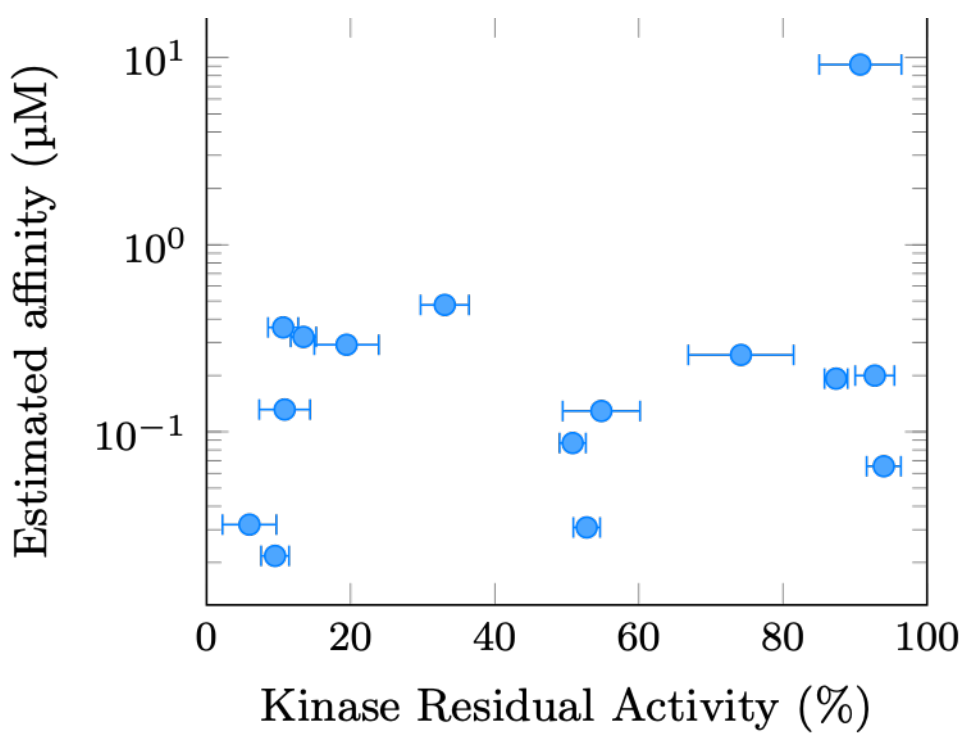

(a)

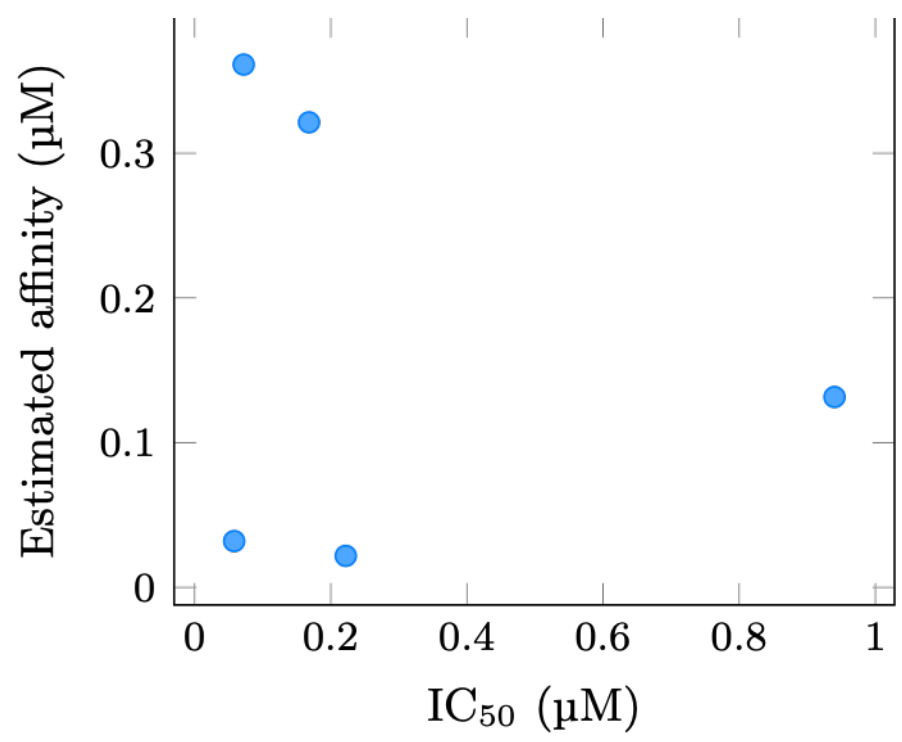

(b)

Text S1: Overlapping binding mode of crystal ligand in AKT2 and docked ligand in PKA

While comparing our post-filtered ligands to ChEMBL, an exact match with a ligand in ChEMBL (CHEMBL597211) was found. A crystal structure of one ligand bound to AKT2 is available (PDB: 2XH5), allowing for structural comparison with the docking pose of the generated ligand in PKA by superimposing the two protein structures (see Figure 4a in main manuscript).

The observed binding mode reveals substantial overlap within the hinge region (AP fragment) and the terminal benzene with its isopropyl substituent attached (partial FP fragment), suggesting an accurate placement of the ligand within the kinase structure and supporting the identification of promising compounds.

Text S2: SeeSAR docking engine

We choose FlexX [6] as the docking engine for our pipeline. FlexX uses a divide-and-conquer algorithm, i.e., it deconstructs and rebuilds the ligand within the binding pocket, generating multiple poses. It then ranks these poses according to FlexX's free energy estimation function [6] and returns - by default - the 10 best-ranked poses. The pharmacophore-based docking module from FlexX [6] is invoked to allow subpocket-based docking. The pharmacophore constraints serve as a surrogate for the subpocket location and guide the placement of the poses during docking. In SeeSAR [7], these pharmacophore constraints are defined as a sphere - with a specific radius - together with a constraint defined by a SMARTS pattern or a function of a substructure (e.g., to form hydrogen bonds). During docking, substructures from the generated poses have to fulfill these spatial and functional constraints. SeeSAR also comes with the scoring function HYDE that estimates the affinity of the given poses from the docked pose using a desolvation-aware scoring function. It thereby optimizes the poses regarding hydrogen bond network, ligand strain energy, and clashes, which can lead to slight changes in docking pose [8]. Note that the command line version of the docking algorithm FlexX is invoked for all core and subsequent template docking steps.

Text S3: Preserved waters

To prepare the cocrystallized PKA structure for docking, we retained water molecules 686, 691, and 696, which correspond to the conserved water clusters I4 and I10 defined by KLIFS [5]. These water clusters are partially preserved in 24 of 38 PKA structures in KLIFS [5] and frequently participate in ligand interactions, making them important for maintaining biologically relevant binding environments. All other water molecules are removed to provide sufficient space for ligand docking.

Text S4: Binding pocket definition & identification

We base the binding pocket definition on the 85 KLIFS-defined binding pocket residues. To define the binding pocket for the docking software FlexX [6], we manually selected the 85 binding pocket residues in the SeeSAR binding site definition step [7]. If a kinase structure is unavailable in the KLIFS database, an additional annotation is necessary to identify the 85 binding pocket residues. We first perform an MSA of the binding pocket sequence of all KLIFS entries for the query kinase using Clustal Omega [9]. From this MSA, the consensus sequence of the binding pocket sequences of this kinase is obtained using the Cons (EMBOSS) [10] functionality. Next, a local alignment of the consensus sequence and the sequence of the given structure is calculated with Blastp, the protein alignment tool of the BLAST [11]. Based on this alignment, we identify the 85 binding pocket residues following the KLIFS numbering scheme for any given kinase structure. The code to perform this alignment is available on GitHub at https://github.com/volkamerlab/KinFragLib_PocketEnum/tree/main/pocket_definition.

Text S5: Subpocket and pharmacophore definition

We represent the subpockets as spheres defined by a subpocket center and a radius. The subpocket center is calculated as the geometric center of the C_α atoms of the subpocket-specific anchor residues, as defined by Sydow *et al.* [12]. The radius of a sphere was determined by visual inspection of multiple structures and is listed in Suppl. Table S5. The constraint of the AP subpocket enforces that the fragment forms at least one hydrogen bond within the hinge region, reflecting that most known Type I and Type II kinase inhibitors are anchored through hinge hydrogen bonds [13]. For the other subpockets, we enforce that each sphere contains at least 3 heavy atoms, based on the maximum common substructure (MCS) of all fragments in KinFragLib. To further ensure correct placement of fragments into the intended subpocket, we exclude the volumes of other subpockets that should not be covered by the growing ligand. For example, if a growing molecule already occupies subpocket A and a fragment is to be placed into subpocket B, then other subpockets, such as C, are excluded during docking. This is enforced with exclusion spheres to be defined in SeeSAR [7].

Text S6: Shannon entropy as a molecular diversity measure

To quantify scaffold diversity, we calculate the Shannon-Entropy [14]

$$\mathrm{SE} = \sum_{\mathrm{i}=1}^{\mathrm{n}} \mathrm{p_i} \log \mathrm{p_i}$$

for the *n* most frequent scaffolds. Here,

$$p_i = \frac{c_i}{P}$$

is the frequency of scaffold *i* in a set of *P* compounds. c_i is thereby the number of compounds that match scaffold *i*.

$$SSE = \frac{SE}{\log n}$$

scales *SE* to a value between 0 and 1. A value close to 1 indicates a high diversity of scaffolds, whereas a value of 0 implies that all compounds share the same scaffold. This entropy-based approach is adapted from Cedillo-González and Medina-Franco [15].

To quantitatively assess scaffold diversity of the Murcko scaffolds, we computed the Shannon Entropy by determining a scaled Shannon Entropy (SSE) of the scaffolds for the post-filtered compound set. The SSE for the Murcko scaffolds is 0.96, indicating a high degree of scaffold entropy, hence a substantial structural diversity within the dataset.

Text S7: Ligand diversity evaluation

To evaluate compound diversity, we analyze scaffold distributions and molecular fingerprint similarities. Scaffolds represent simplified molecular cores. We compute them using the method proposed by Bemis and Murcko [16], which defines scaffolds as the union of ring systems and linkers, explicitly excluding side chains.

In addition to scaffold analysis, we assess the diversity at the molecular feature level using fingerprint similarity. Specifically, we employ RDKit topological fingerprints to encode molecular features. Pairwise similarity between molecules is then computed using the Tanimoto coefficient [17], which quantifies the ratio of shared to total features between two molecules A and B. Lower average similarity values indicate greater diversity within the compound set.

Text S8: ChEMBL database comparison

To assess the novelty and relevance of the generated compounds, we performed a comparative analysis against the ChEMBL database [18]. Specifically, we screened for exact structural matches and structurally similar analogs within the database. We retrieved ChEMBL version 33 [19], comprising 2 372 672 compounds. All entries were standardized using RDKit [20] and converted to InChI strings, enabling consistent representation and facilitating exact match detection. To find the most similar ChEMBL ligand for each generated compound, we compare the RDKit (topological) fingerprints [21] using the Tanimoto coefficient. As before, we computed pairwise molecular similarities between each generated compound and all ChEMBL entries. Molecular features were encoded using RDKit topological fingerprints [21], and similarity was quantified using the Tanimoto coefficient [17]. For each

generated compound, the most similar ChEMBL ligand was identified based on the highest Tanimoto score.

Text S9: Drug-likeness analysis of generated compounds

To assess the drug-likeness of the post-filtered compounds, we consider the molecular properties from the Rule of Five (Ro5) and Veber's criteria: molecular weight (MW), hydrogen-bond donors (HBD), hydrogen-bond acceptors (HBA), logP, topological polar surface area (TPSA), and rotatable bonds (RB). While the proposed thresholds of these properties were primarily proposed to assess the oral bioavailability, they remain useful indicators for the pharmaceutical relevance of a molecule.

For two properties (TPSA and HBA), there are no compound violations among the generated ligands. Two properties (HBD and MW) have a few violations with values slightly above the threshold, and two properties - namely RB and logP - filter out larger outliers (see Figure 2f in main manuscript). We see that only 37 (3%) of the compounds have a logP value above the Rule of Five threshold (> 5) and 64 (5%) of the compounds have more than 5 RB, i.e., might tend to be too flexible. Hence, 1154 out of 1196 compounds (93%) fulfill all drug-like filters.

Text S10: Program settings for case study on PKA


```
{
"pdbCode": "5n1f",
"CoreSubpocket": "AP",
"Subpockets": ["FP", "GA"],
"KinFragLib": "KinFragLib/data",
"Config": "config_smarts_exclude",
"FlexX": "flexx-6.0.0-Linux-x64/flexx",
"NumberFragmentsPerIterations": 300,
"NumberPosesPerFragment": 5,
"Filters": {
"pains": {},
"brenk": {"path_data": "KinFragLib/data/filters/Brenk"},
"ro3": {},
"qed": {"cutoff_val": 0.492},
"syba": {"cutoff_val": 0}
},
"UseClusterBasedPoseFiltering": true,
"DistanceThresholdClustering": 1.5,
"NumberThreads": 256,
"UseHyde": true,
"Hyde": "hydescorer-2.0.0-Linux-x64/hydescorer",
"HydeDisplacementCutoff": 2.5,
"Seed": 42,
"UseClusterBasedFragmentFiltering": true,
"PSoftMin": 1
}

```

Scheme S1: Synthesis of 7, 8, 9[a]

The compounds **7**, **8**, and **9**, are accessible via the common intermediate **3**, followed by a subsequent Suzuki reaction with the respective boronic acids. The latter are commercially available for **8** and **9**; whereas in case of **7**, it is accessible via a two-step synthesis, as follows (Scheme 1). Reaction of aniline **1** with 2-methylpropane-2-sulfinyl chloride leads to the sulfinamide **2** which is subsequently oxidized to sulfonamide **3** [21, 22]. The boronic acid pinacol ester **6** was synthesized starting from bromobenzthiophene methyl ester **4** via its corresponding amide **5** and subsequent borylation under standard Suzuki coupling conditions.

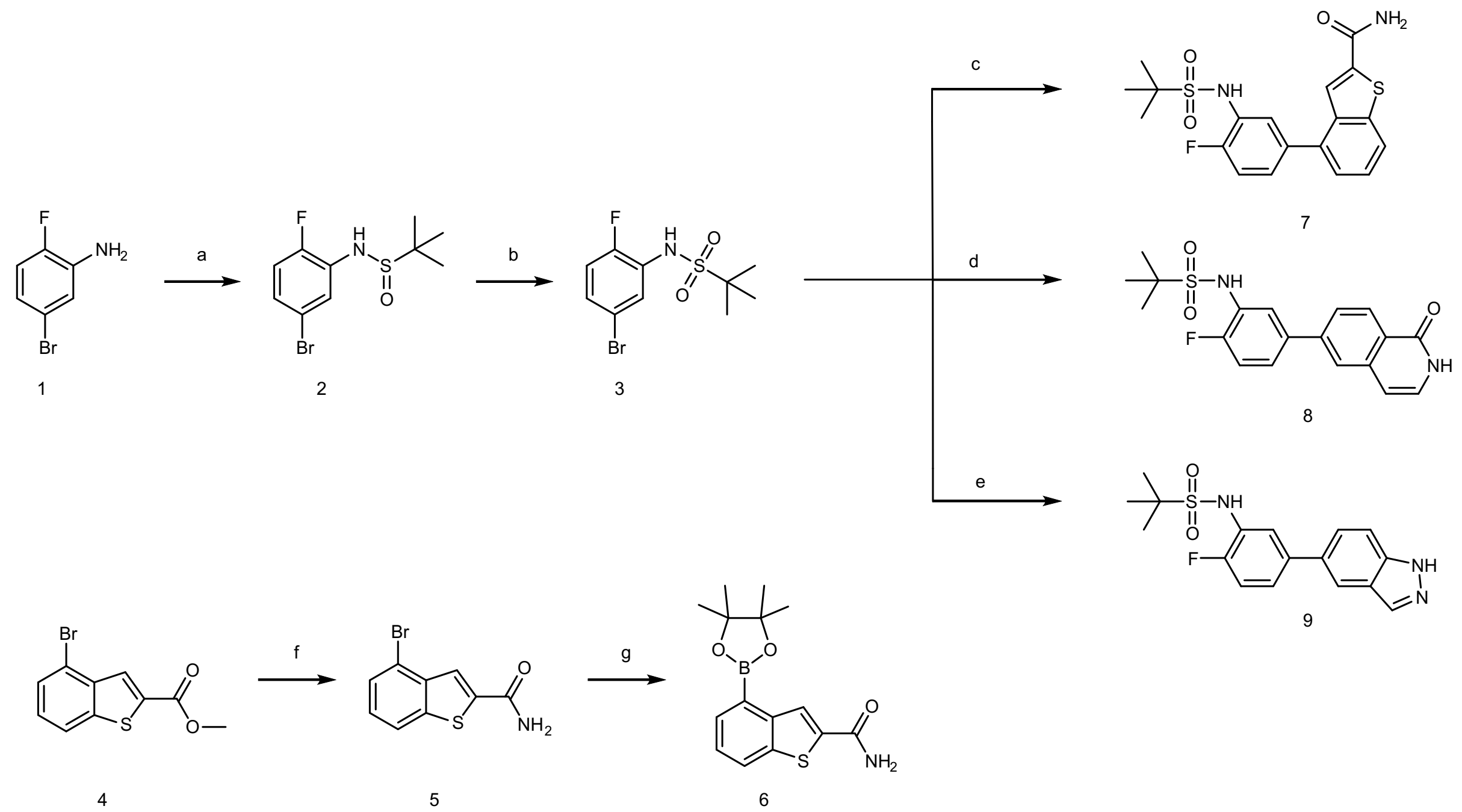

[a] reagents and conditions: a) 2-methylpropane-2-sulfinyl chloride, DCM, -78 °C → rt, 3.5 h, 71%; b) *m*CPBA, DCM, rt, 3.5 h, 80%; c) **6**, Na_2CO_3, $Pd(PPh_3)_4$, DME/H_2O (2:1), MW (300 W), 130 °C, 1 h, 44%; d) 6-(4,4,5,5-tetramethyl-1,3,2-dioxaborolan-2-yl)isoquinolin-1(2*H*)-one, Na_2CO_3, $Pd(PPh_3)_4$, DME/H_2O (2:1), MW (300 W), 130 °C, 1 h, 41%; e) 5-(4,4,5,5-tetramethyl-1,3,2-dioxaborolan-2-yl)-1*H*-indazole, Na_2CO_3, $Pd(PPh_3)_4$, DME/H_2O (2:1), MW (300 W), 130 °C, 1 h, 43%; f) 7 M NH_3 in MeOH, 65 °C, overnight, 82%; g) b*is*-(pinacolato)-diborane, KOAc, Pd(dppf)Cl_2 DCM adduct, 1,4-dioxane, 100 °C, overnight, 86%.

Scheme S2: Synthesis of 17[a]

Compound **17** was synthesized according to Scheme 2, following a procedure described by Boehm *et al* [24]. In brief, reaction of 1-(2,6-dihydroxyphenyl)ethan-1-one **10** with hydrazine monohydrate afforded compound **11**. TBS protection of the phenol followed by Boc-protection of the indazole NH and subsequent phenol deprotection furnished intermediate **12**. In parallel, coupling of thiophene carboxylic acid chloride **13** and 3-aminopropan-1-ol **14** rendered **15**, which, applying a Mitsunobu coupling reaction with the above intermediate **12** using a method by Rueedi *et al.* [25], yielded **16**, which was converted to **17** in the final step by N-Boc-deprotection.

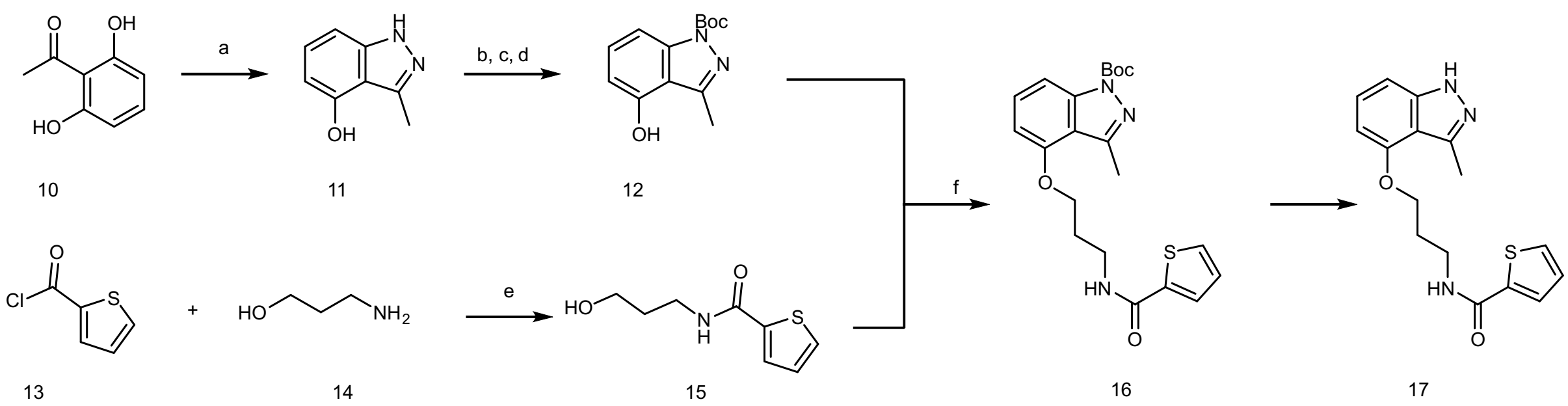

[a] reagents and conditions: a) hydrazine monohydrate, ethylene glycol, 20 min, rt, then 160 °C, 90 min, 74%; b) TBSCl, imidazole, DMF, rt, 16 h, 64%; c) DMAP, DMF, Boc_2O, MeCN, rt, 2 h; d) TBAF, THF, rt, 2 h, 41% over two steps; e) Et_3N, DCM, rt, 22 h, 82%; f) diisopropyl azodicarboxylate, triphenylphosphine, THF, 0 °C → rt, 24 h; g) TFA, DCM, rt, 16 h, 15% over two steps.

Scheme S3: Synthesis of 20[a]

A Suzuki coupling of boronic acid pinacol ester **18** with 2,4-dichloropyrimidine **19**, followed by chlorine substitution with ethylamine, afforded **20** (Scheme 3).

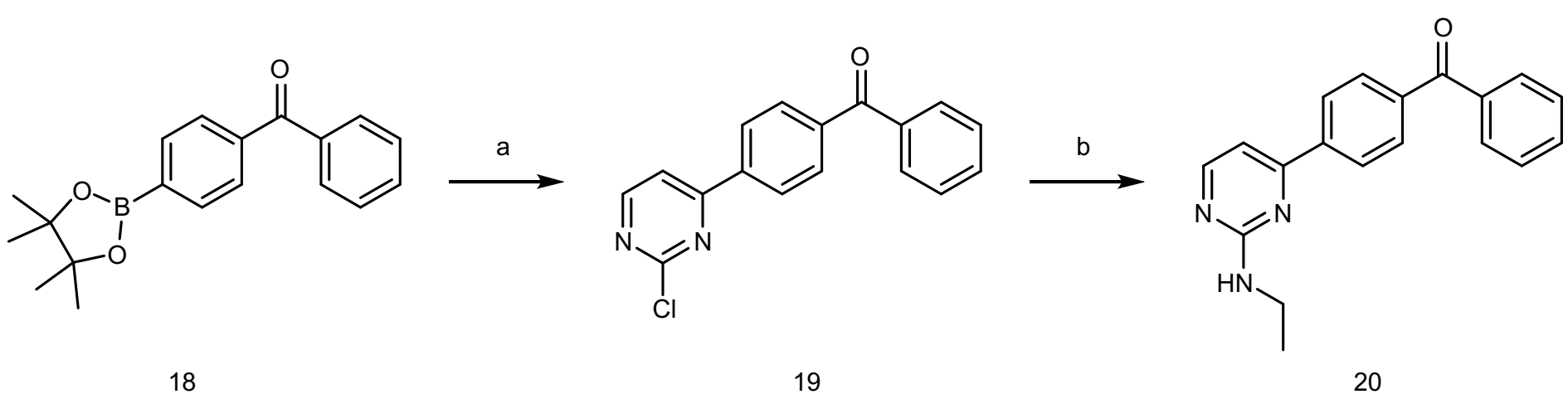

[a] reagents and conditions: a) 2,4-dichloropyrimidine, $Pd(PPh_3)_4$, K_2CO_3, 1,4-dioxane/H_2O (4:1), 100 °C, 16 h, 35%; b) ethylamine hydrochloride, *N*,*N*-diisopropylethylamine, MeCN, 150 °C, 1 h MW (100 W), 150 °C, 67%.

Scheme S4: Synthesis of 24 [a]

Starting point for the synthesis of target compound **24** was 2-amino-6-bromobenzoic acid (**21**), which was first reduced to the corresponding commercially available but quite expensive alcohol **22**. After protecting the alcohol function as TBS ether, the aromatic amino function was acylated with benzoyl chloride rendering benzamide **23**, which was then reacted with the corresponding boronic acid pinacol ester using a Suzuki coupling in the next step. Under the chosen reaction conditions, a mixture of the TBS-protected precursor and the already deprotected target compound **24** was formed, which could be isolated in moderate yield by column chromatography.

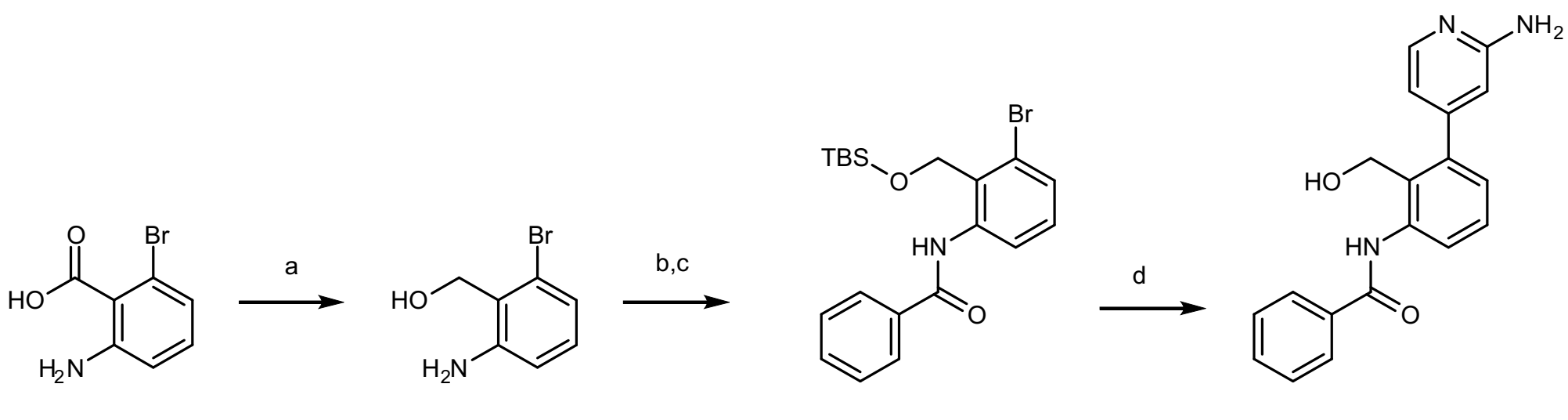

21 22 23 24

[a] reagents and conditions: a) LAH 2.4 M in THF, Na_2SO_4*10 H_2O, THF, 0 °C, rt, 16 h, 63%; b) TBSCl, imidazole, DMF, 0 °C, rt, 4 h, 96%; c) BzCl, Et_3N, DCM, 0 °C, rt, 5 h, 90%; d) 4-(4,4,5,5-tetramethyl-1,3,2-dioxaborolan-2-yl)pyridin-2-amine, $Pd(PPh_3)_4$, DME/H_2O (2:1), MW (300 W), 130 °C, 1 h, 24%.

Scheme S5: Synthesis of 26 [a]

As shown, pyrrolo-pyrimidine **26** was obtained via Suzuki coupling of 4-bromopyrrolopyrimidine **25** and boronic acid pinacol ester **18** in moderate yield.

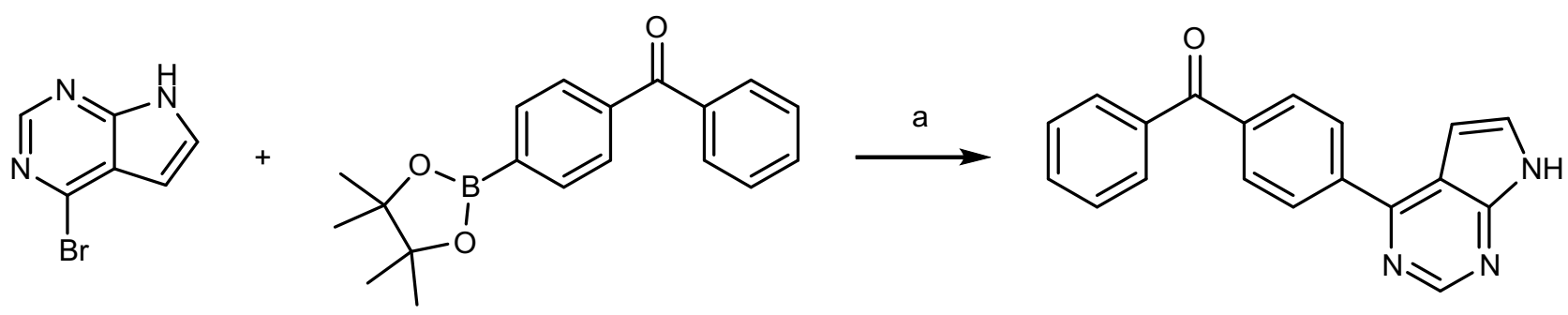

25 18 26

[a] reagents and conditions: a) $Pd(PPh_3)_4$, K_2CO_3, 1,4-dioxane/H_2O (4:1), 100 °C, 18 h, 16%.

Scheme S6: Synthesis of 33[a]

A Buchwald-Hartwig amination of aryl bromide **31** and 3-morpholinopropan-1-amine **32** afforded **33** in just a single step (Scheme 6).

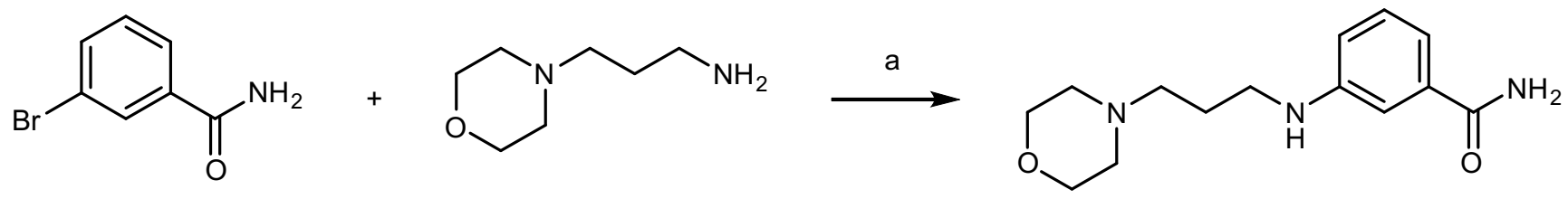

31 32 33

[a] reagents and conditions: a) $Pd(dba)_2$, *t*BuBrettPhos, K_3PO_4, 1,4-dioxane, 100 °C, 22 h, 18%.

N-(5-Bromo-2-fluorophenyl)-2-methylpropane-2-sulfinamide (2)

A solution of 5-bromo-2-fluoroaniline (**1**) (0.80 mL, 7.11 mmol, 2.00 equiv) in DCM (7.10 mL) was cooled to -78 °C. Then, a solution of 2-methylpropane-2-sulfinyl chloride (0.44 mL, 3.56 mmol, 1.00 equiv) in DCM (2.10 mL) was added dropwise, the reaction mixture was slowly warmed to room temperature and stirred for an additional 3.5 h. After washing with water (3 x 10 mL), the solvent was removed under reduced pressure. The residue was adsorbed on silica and purified by column chromatography (cyclohexane/EtOAc 0 - 30% over 20 min) yielding **2** (745 mg, 2.53 mmol, 71%) as a white solid.

^{1}H NMR (400 MHz, DMSO-d_6, 300 K): δ (ppm) = 7.86 (s, 1H), 7.42 (d, $^4J_{H,F}$ = 7.8 Hz, 1H), 7.23 - 7.18 (m, 2H), 1.25 (s, 9H); ^{13}C NMR (125 MHz, DMSO-d_6, 300 K): δ (ppm) = 152.9 (d, $^1J_{C,F}$ = 244 Hz), 132.5 (d, $^2J_{C,F}$ = 13 Hz), 126.3 (d, $^3J_{C,F}$ = 8 Hz), 124.6 (d, $^4J_{C,F}$ = 3 Hz), 118.2 (d, $^2J_{C,F}$ = 21 Hz), 116.0 (d, $^3J_{C,F}$ = 4 Hz), 56.9, 22.2; HR-MS *m/z* $[M+H]^+$ calcd. for $C_{10}H_{13}{}^{79}BrFNOSH$: 293.9958, found: 293.9958; Purity was determined by qNMR using 1,2,4,5-tetrachloro-3-nitro-benzene as internal standard: 99.9%; mp: 95.1 °C.

N-(5-Bromo-2-fluorophenyl)-2-methylpropane-2-sulfonamide (3)

N-(5-Bromo-2-fluorophenyl)-2-methylpropane-2-sulfinamide (**2**) (500 mg, 1.70 mmol, 1.00 equiv) was dissolved in DCM (6 mL), *m*CPBA (587 mg, 3.40 mmol, 2.00 equiv) was added and the reaction mixture was stirred for 3.5 h at room temperature [21, 22]. After quenching by adding sodium hydrogen sulfite (300 mg), the resulting suspension was filtered, water (20 mL) was added to the filtrate, and it was extracted with ethyl acetate (3 x 20 mL). The combined organic layers were washed with saturated $NaHCO_3$ solution (20 mL), dried over $MgSO_4$, filtered, adsorbed onto silica and purified by column

chromatography (cyclohexane/EtOAc 0 - 30% over 20 min). After recrystallization from MeOH, **3** (421 mg, 1.36 mmol, 80%) was obtained as a white solid.

^{1}H NMR (400 MHz, DMSO-d_6, 300 K): δ (ppm) = 9.78 (s, 1H), 7.65 (d, $^{4}J_{H,F}$ = 7.1 Hz), 7.40 - 7.36 (m, 1H), 7.26 (dd, $^{3}J_{H,F}$ = 11.9 Hz, ^{3}J = 8.9 Hz, 1H), 1.28 (s, 9H); ^{13}C NMR (125 MHz, DMSO-d_6, 300 K): δ (ppm) = 153.8 (d, $^{1}J_{H,F}$ = 247 Hz), 128.9 (d, $^{3}J_{H,F}$ = 8 Hz), 128.2 (d, $^{2}J_{H,F}$ = 14 Hz), 128.0, 117.9 (d, $^{2}J_{H,F}$ = 22 Hz), 115.7 (d, $^{3}J_{H,F}$ = 4 Hz), 60.9, 23.9; HR-MS *m/z* $[M-H]^-$ calcd. for $C_{10}H_{12}{}^{79}BrFNO_2S$: 307.9762, found: 307.9766; Purity was determined by qNMR using 1,2,4,5-tetrachloro-3-nitro-benzene as internal standard: 99.8%; mp: 179.0 °C.

4-Bromobenzo[b]thiophene-2-carboxamide (**5**)

Methyl 4-bromobenzo[*b*]thiophene-2-carboxylate (**4**) (300 mg, 1.10 mmol, 1.00 equiv) was mixed with 7 M NH_3 in MeOH (3.50 mL) and stirred overnight at 65 °C. After concentrating the reaction mixture, the crude product was purified by column chromatography (cyclohexane/EtOAc 0 - 50% over 25 min), yielding **5** (230 mg, 0.90 mmol, 82%) as a white solid. ^{1}H NMR (400 MHz, DMSO-d_6, 300 K): δ (ppm) = 8.42 (br s, 1H), 8.19 (s, 1H), 8.04 (d, ^{3}J = 8.0 Hz, 1H), 7.69 (br s, 1H), 7.67 (dd, ^{3}J = 7.7 Hz, ^{4}J = 0.7 Hz, 1H), 7.38 (dd, ^{3}J = 8.0 Hz, ^{3}J = 7.8 Hz, 1H); ^{13}C NMR (125 MHz, DMSO-d_6, 300 K): δ (ppm) = 162.8, 141.9, 141.3, 138.9, 128.0, 127.3, 124.6, 122.4, 117.5; HR-MS *m/z* $[M+Na]^+$ calcd. for $C_9H_6{}^{79}BrFNOSNa$: 277.9246, found: 277.9233; Purity was determined by qNMR using ethylene carbonate as internal standard: 96.3%; mp: 179.6 °C.

4-(4,4,5,5-Tetramethyl-1,3,2-dioxaborolan-2-yl)benzo[b]thiophene-2-carboxamide (**6**)

In a N_2-flask, KOAc (1025 mg, 4.68 mmol, 3.00 equiv) was first dried at 120 °C. Subsequently, 4-bromobenzo[b]thiophene-2-carboxamide (**5**) (400 mg, 1.56 mmol, 1.00 equiv), *bis*-(pinacolato)-diborane (595 mg, 2.34 mmol, 1.50 equiv) and 1,4-dioxane (7.80 mL) were added under an argon atmosphere. After degassing the reaction mixture with argon for 15 minutes, Pd(dppf)Cl_2 * DCM (127 mg, 0.156 mmol, 0.10 equiv) was added and the reaction was heated to reflux overnight. After cooling to room temperature, the reaction mixture was concentrated, adsorbed onto silica, and purified by column chromatography (cyclohexane/EtOAc 0 - 80% over 25 min). The product thus obtained was suspended in cyclohexane and filtered. **6** (408 mg, 1.35 mmol, 86%) was obtained as a light pink solid, which was still slightly contaminated with *bis*-(pinacolato)-diborane, and was used in the next step without further purification.

^{1}H NMR (400 MHz, DMSO-d_6, 300 K): δ (ppm) = 8.35 - 8.31 (m, 2H), 8.13 (d, ^{3}J = 8.0 Hz, 1H), 7.78 (d, ^{3}J = 7.1 Hz, 1H), 7.61 (br s, 1H), 7.44 (dd, ^{3}J = 8.0 Hz, ^{3}J = 7.1 Hz, 1H), 1.37 (s, 14H); ^{13}C NMR (125 MHz, DMSO-d_6, 300 K): δ (ppm) = 163.3, 143.1, 140.6, 140.4, 132.6, 126.4, 125.8, 125.2, 83.8, 24.6; HR-MS

m/z $[M+H]^+$ calcd. for $C_{15}H_{18}BNO_3SH$: 304.1173, found: 304.1159; Purity was determined by qNMR using ethylene carbonate as internal standard: 97.2%; mp: 171.1 °C.

4-{3-[(1,1-Dimethylethyl)sulfonamido]-4-fluorophenyl}benzo[b]thiophene-2-carboxamide (7)

A microwave vessel was charged with *N*-(5-bromo-2-fluorophenyl)-2-methylpropane-2-sulfonamide (**3**) (300 mg, 0.97 mmol, 1.00 equiv), 4-(4,4,5,5-tetramethyl-1,3,2-dioxaborolan-2-yl)benzo[b]thiophene-2-carboxamide (**6**) (352 mg, 1.16 mmol, 1.20 equiv), Na_2CO_3 (307 mg, 2.90 mmol, 3.00 equiv) and $Pd(PPh_3)_4$ (112 mg, 0.10 mmol, 10 mol%) under an argon atmosphere. Following the addition of DME/water (2:1, 5 mL), the reaction was stirred for 1 h at 130 °C under microwave conditions. The reaction mixture was allowed to cool to room temperature, then concentrated under reduced pressure and adsorbed onto silica. Column chromatography (DCM/MeOH 0 - 5% over 25 min) yielding **7** (173 mg, 0.43 mmol, 44%) as a yellowish solid.

^{1}H NMR (400 MHz, DMSO-d_6, 300 K): δ (ppm) = 9.70 (s, 1H), 8.16 (br s, 1H), 8.08 (s, 1H), 8.03 (d, ^3J = 8.0 Hz, 1H), 7.67 (d, ^3J = 7.8 Hz, 1H), 7.57 (br s, 1H), 7.54 (dd, ^3J = 8.0 Hz, ^3J = 7.8 Hz, 1H), 7.44 - 7.41 (m, 2H), 7.37 (d, ^3J = 7.3 Hz, 1H), 1.33 (s, 9H); ^{13}C NMR (125 MHz, DMSO-d_6, 300 K): δ (ppm) = 163.2, 154.4 (d, $^1J_{C,F}$ = 244 Hz), 141.3, 140.8, 137.13, 137.06, 136.0 (d, $^3J_{C,F}$ = 4 Hz), 126.8 (d, $^3J_{C,F}$ = 8 Hz), 126.6 (d, $^2J_{C,F}$ = 13 Hz), 126.5, 126.3, 125.0, 123.9, 122.3, 116.1 (d, $^2J_{C,F}$ = 20 Hz), 60.7, 24.0; HR-MS *m/z* $[M-H]^-$ calcd. for $C_{19}H_{18}FN_2O_3S_2$: 405.0748, found: 405.0738; Purity was determined by qNMR using 1,2,4,5-tetrachloro-3-nitro-benzene as internal standard: 94.6%; mp: 219.8 °C.

N-[2-Fluoro-5-(1-oxo-1,2-dihydroisoquinolin-6-yl)phenyl]-2-methylpropane-2-sulfonamide (8)

A microwave vessel was charged with *N*-(5-bromo-2-fluorophenyl)-2-methylpropane-2-sulfonamide (**3**) (300 mg, 0.97 mmol, 1.00 equiv), 6-(4,4,5,5-tetramethyl-1,3,2-dioxaborolan-2-yl)isoquinolin-1(2*H*)-one (315 mg, 1.16 mmol, 1.20 equiv), Na_2CO_3 (307 mg, 2.90 mmol, 3.00 equiv) and $Pd(PPh_3)_4$ (112 mg, 0.10 mmol, 10 mol%) under an argon atmosphere. Following the addition of DME/water (2:1, 5 mL), the reaction was stirred for 1 h at 130 °C under microwave conditions. After cooling to room temperature, the reaction mixture was concentrated, adsorbed onto silica, and purified by column chromategraphy (DCM/MeOH 0 - 15% over 25 min and cyclohexane/EtOAc 40 - 100% over 60 min), yielding **8** (148 mg, 0.43 mmol, 41%) as a white solid.

^{1}H NMR (400 MHz, DMSO-d_6, 300 K): δ (ppm) = 11.24 (s, 1H), 9.68 (s, 1H), 8.25 (d, ^3J = 8.5 Hz, 1H), 7.88 - 7.85 (m, 2H), 7.69 (dd, ^3J = 8.2 Hz, ^4J = 1.8 Hz, 1H), 7.62 (ddd, ^3J = 8.6 Hz, $^4J_{H,F}$ = 4.5 Hz, ^4J = 2.5 Hz, 1H), 7.40 (dd, $^3J_{H,F}$ = 10.3 Hz, ^3J = 8.7 Hz, 1H), 7.20 (dd, ^3J = 6.9 Hz, ^3J = 6.0 Hz, 1H), 6.64 (d, ^3J = 7.3 Hz, 1H), 1.31 (s, 9H); ^{13}C NMR (125 MHz, DMSO-d_6, 300 K): δ (ppm) = 161.6, 155.0 (d, $^1J_{C,F}$ = 247 Hz), 142.4, 138.4, 135.8 (d, $^3J_{C,F}$ = 4 Hz), 129.5, 127.6, 126.9 (d, $^2J_{C,F}$ = 13 Hz), 125.4 (d, $^3J_{C,F}$ = 7 Hz), 125.2, 125.1,

124.8, 123.8, 116.5 (d, ${}^{2}J_{C,F}$ = 20 Hz), 104.7, 60.8, 24.0; HR-MS *m/z* [M-H]$^-$ calcd. for $C_{19}H_{18}FN_2O_3S$: 373.1028, found: 373.1019; Purity was determined by qNMR using dimethylsulfone as internal standard: 91.1%; mp: 273.3 °C.

N-[2-Fluoro-5-(1*H*-indazol-5-yl)phenyl]-2-methylpropane-2-sulfonamide (9)

A microwave vessel was charged with *N*-(5-bromo-2-fluorophenyl)-2-methylpropane-2-sulfonamide (**3**) (300 mg, 0.97 mmol, 1.00 equiv), 5-(4,4,5,5-tetramethyl-1,3,2-dioxaborolan-2-yl)-1*H*-indazole (260 mg, 1.06 mmol, 1.10 equiv), Na_2CO_3 (307 mg, 2.90 mmol, 3.00 equiv) and $Pd(PPh_3)_4$ (112 mg, 0.10 mmol, 10 mol%) under an argon atmosphere. Following the addition of DME/water (2:1, 5 mL), the reaction was stirred for 1 h at 130 °C under microwave conditions. After cooling to room temperature, the reaction mixture was concentrated, adsorbed onto silica, and purified by column chromatography (cyclohexane/EtOAc 10 - 100% over 30 min), yielding **9** (144 mg, 0.41 mmol, 43%) as a white solid.

^{1}H NMR (400 MHz, DMSO-d_6, 300 K): δ (ppm) = 13.12 (s, 1H), 9.61 (s, 1H), 8.14 (s, 1H), 7.96 (s, 1H), 7.79 (dd, ${}^{4}J_{H,F}$ = 7.8 Hz, ${}^{4}J$ = 2.5 Hz, 1H), 7.63 (d, ${}^{3}J$ = 8.7 Hz, 1H), 7.58 (dd, ${}^{3}J$ = 8.7 Hz, ${}^{4}J$ = 1.6 Hz, 1H), 7.51 (ddd, ${}^{3}J$ = 8.5 Hz, ${}^{4}J_{H,F}$ = 4.6 Hz, ${}^{4}J$ = 2.5 Hz, 1H), 7.33 (dd, ${}^{3}J_{H,F}$ = 10.3 Hz, ${}^{3}J$ = 8.7 Hz, 1H), 1.32 (s, 9H); ^{13}C NMR (125 MHz, DMSO-d_6, 300 K): δ (ppm) = 154.1 (d, ${}^{1}J_{C,F}$ = 247 Hz), 139.3, 137.4 (d, ${}^{3}J_{C,F}$ = 4 Hz), 134.0, 131.4, 126.6 (d, ${}^{2}J_{C,F}$ = 13 Hz), 125.4, 124.8 (d, ${}^{3}J_{C,F}$ = 8 Hz), 124.7, 123.5, 118.2, 116.2 (d, ${}^{2}J_{C,F}$ = 20 Hz), 110.7, 60.7, 24.0; HR-MS *m/z* [M-H]$^-$ calcd. for $C_{17}H_{17}FN_3O_2S$: 346.1031, found: 346.1023; Purity was determined by qNMR using 1,2,4,5-tetrachloro-3-nitro-benzene as internal standard: 99.8%; mp: 250.7 °C.

3-Methyl-1*H*-indazol-4-ol (11)

Hydrazine monohydrate (656 mg, 13.10 mmol, 2.00 equiv) was dissolved in ethylene glycol (5 mL), a solution of 1-(2,6-dihydroxyphenyl)ethan-1-one (**10**) (1000 mg, 6.57 mmol, 1.00 equiv) in ethylene glycol (15 mL) was added dropwise and the mixture was stirred at room temperature for 20 min and afterwards at 160 °C for 90 min. The mixture was allowed to cool to room temperature, water (200 mL) was added and pH was adjusted to 6 by addition of aqueous 2 M HCl solution and the reaction mixture was extracted with EtOAc (3 × 200 mL). The combined organic layers were washed with saturated NaCl solution, dried over $MgSO_4$, filtered and the solvent was removed under reduced pressure. The crude product was adsorbed onto silica and purified by column chromatography (cyclohexane/EtOAc 0 - 70% over 35 min) to give 3-methyl-1*H*-indazol-4-ol (**11**) (722 mg, 4.87 mmol, 74%) as a light brown solid.

^{1}H NMR (500 MHz, DMSO-d_6, 300 K): δ (ppm) = 12.31 (br s, 1H), 9.85 (s, 1H), 7.02 (dd, ${}^{3}J$ = 8.2 Hz, ${}^{3}J$ = 7.6 Hz, 1H), 6.79 (d, ${}^{3}J$ = 8.2 Hz, 1H), 6.31 (d, ${}^{3}J$ = 7.3 Hz, 1H), 2.55 (s, 3H); ^{13}C NMR (125 MHz, DMSO-

d$_6$, 300 K): δ (ppm) = 152.4, 143.5, 140.2, 127.3, 113.0, 102.6, 100.6, 13.9; HR-MS *m/z* $[M-H]^-$ calcd. for $C_8H_7N_2O$: 147.0564, found: 147.0546; Purity was determined by qNMR using 1,2,4,5-tetrachloro-3-nitro-benzene as internal standard: 99.7%; mp: 198.1 °C.

tert-Butyl 4-hydroxy-3-methyl-1*H*-indazole-1-carboxylate (**12**)

To an ice-cold solution of **11** (1.27 g, 8.57 mmol, 1.00 equiv) in DMF (30 mL), TBSCl (1.48 g, 9.86 mmol, 1.15 equiv) and imidazole (2.92 g, 42.85 mmol, 5.00 equiv) were added, the reaction mixture was slowly warmed to room temperature and stirred for additional 16 h, after which complete conversion was detected via TLC. The reaction mixture was quenched with water and extracted with EtOAc (3 × 50 mL), the combined organic layers were washed with aqueous 5% LiCl solution, saturated NaCl solution, dried over $MgSO_4$, filtered and the solvent was removed under reduced pressure. The crude product was adsorbed onto silica and purified by column chromatography (cyclohexane/EtOAc 0 - 50% over 20 min) to give 4-[(*tert*-butyldimethylsilyl)oxy]-3-methyl-1*H*-indazole (1.43 g, 5.46 mmol, 64%) as a light beige solid [24].

^{1}H NMR (500 MHz, DMSO-*d*$_6$, 300 K): δ (ppm) = 12.49 (s, 1H), 7.12 (dd, ^3J = 8.0 Hz, ^3J = 7.8 Hz, 1H), 6.98 (d, ^3J = 8.2 Hz, 1H), 6.39 (d, ^3J = 7.3 Hz, 1H), 2.57 (s, 3H), 1.01 (s, 9H), 0.30 (s, 6H); ^{13}C NMR (125 MHz, DMSO-*d*$_6$, 300 K): δ (ppm) = 149.8, 143.4, 140.0, 127.1, 115.2, 106.5, 103.0, 25.6, 18.0, 14.3, -4.3; HR-MS *m/z* $[M+H]^+$ calcd. for $C_{14}H_{22}N_2OSiH$: 263.1574, found: 263.1571; mp: 112.4 °C.

4-[(*tert*-butyldimethylsilyl)oxy]-3-methyl-1*H*-indazole (1.20 g, 4.56 mmol, 1.00 equiv) and triethylamine (0.70 mL, 5.02 mmol, 1.10 equiv) were dissolved in MeCN (9 mL) and the solution was cooled to 0 °C. A solution of di-*tert*-butyl dicarbonate (1.17 mL, 5.47 mmol, 1.20 equiv) in MeCN (6 mL) was added dropwise. The reaction mixture was stirred at room temperature for 2 h. The solvent was removed under reduced pressure and the crude product was adsorbed onto silica and purified by column chromatography (cyclohexane/EtOAc 0 - 20% over 20 min) to give *tert*-butyl 4-[(*tert*-butyldimethylsilyl)oxy]-3-methyl-1*H*-indazole-1-carboxylate (0.95 g, 2.63 mmol) as a light orange oil, which according to the NMR spectra turned out to be slightly impure.

^{1}H NMR (250 MHz, DMSO-*d*$_6$, 300 K): δ (ppm) = 7.62 (d, ^3J = 8.2 Hz, 1H), 7.41 (dd, ^3J = 8.3 Hz, ^3J = 8.1 Hz, 1H), 6.73 (d, ^3J = 7.5 Hz, 1H), 2.58 (s, 3H), 1.61 (s, 9H), 1.00 (s, 9H), 0.33 (s, 6H); HR-MS *m/z* $[M+Na]^+$ calcd. for $C_{19}H_{30}N_2O_3SiNa$: 385.1918, found: 385.1912.

To a solution of *tert*-butyl 4-[(*tert*-butyldimethylsilyl)oxy]-3-methyl-1*H*-indazole-1-carboxylate (869 mg, 2.40 mmol, 1.00 equiv) in THF (10 mL), 1 M TBAF in THF (3.60 mL, 3.60 mmol, 1.50 equiv) was added and the reaction mixture was stirred at room temperature for 2 h. The mixture was quenched with MeOH, the solvents were removed under reduced pressure and the crude product was adsorbed onto silica and purified by column chromatography (cyclohexane/EtOAc 0 - 100% over 12 min) to give

tert-butyl 4-hydroxy-3-methyl-1*H*-indazole-1-carboxylate (**12**) (459 mg, 1.85 mmol, 41% over two steps) as a light orange solid.

^{1}H NMR (500 MHz, DMSO-d_6, 300 K): δ (ppm) = 10.43 (br s, 1H), 7.44 (d, ^{3}J = 8.2 Hz, 1H), 7.31 (dd, ^{3}J = 8.0 Hz, 1H), 6.64 (d, ^{3}J = 7.8 Hz, 1H), 2.57 (s, 3H), 1.61 (s, 9H); ^{13}C NMR (125 MHz, DMSO-d_6, 300 K): δ (ppm) = 152.8, 148.7, 147.3, 142.0, 130.5, 115.2, 107.8, 104.8, 83.6, 27.7, 14.2; HR-MS *m/z* $[M-H]^-$ calcd. for $C_{13}H_{15}N_2O_3$: 247.1088, found: 247.1086; Decomposition at 176 °C.

N-(3-Hydroxypropyl)thiophene-2-carboxamide (**15**)

3-Aminopropan-1-ol (**14**) (1.04 mL, 13.60 mmol, 2.00 equiv) and triethylamine (1.90 mL, 13.60 mmol, 2.00 equiv) were dissolved in DCM (40 mL). Thiophene-2-carbonyl chloride **(13)** (0.73 mL, 6.82 mmol, 1.00 equiv) was dissolved in DCM (10 mL) and added dropwise. The mixture was stirred at room temperature for 22 h. The reaction mixture was quenched with MeOH and the solvents were removed under reduced pressure. The residue was redissolved in EtOAc (20 mL), washed with aqueous 2 M K_2CO_3 solution, saturated NaCl solution, dried over $MgSO_4$, filtered and the solvent was removed under reduced pressure. Drying *in vacuo* yielded *N*-(3-hydroxypropyl)thiophene-2-carboxamide (**15**) (1.04 g, 5.60 mmol, 82%) as an off-white oil.

^{1}H NMR (500 MHz, DMSO-d_6, 300 K): δ (ppm) = 8.42 (t, ^{3}J = 5.0 Hz, 1H), 7.73–7.71 (m, 2H), 7.13 (dd, ^{3}J = 4.8 Hz, ^{3}J = 5.0 Hz, 1H), 4.44 (t, ^{3}J = 5.3 Hz, 1H), 3.46 (q, ^{3}J = 6.4 Hz, 2H), 3.46 (sm, 2H), 3.28 (sm, 2H), 1.66 (sm, 2H); ^{13}C NMR (125 MHz, DMSO-d_6, 300 K): δ (ppm) = 161.0, 140.2, 130.4, 127.7, 127.7, 58.5, 36.4, 32.4; HR-MS *m/z* $[M+Na]^+$ calcd. for $C_8H_{11}NO_2SNa$: 208.0403, found: 208.0398; Purity was determined by qNMR using dimethylsulfone as internal standard: 98.6%.

tert-Butyl 3-methyl-4-[3-(thiophene-2-carboxamido)propoxy]-1*H*-indazole-1-carboxylate (**16**)

12 (400 mg, 1.61 mmol, 1.00 equiv), **15** (298 mg, 1.61 mmol, 1.00 equiv) and triphenylphosphine (635 mg, 2.42 mmol, 1.50 equiv) were suspended in THF (10 mL). The reaction mixture was cooled to 0 °C upon which diisopropyl azodicarboxylate (0.48 mL, 2.42 mmol, 1.50 equiv) was added dropwise over a period of 10 min. After reaching room temperature, the reaction mixture was stirred for an additional 24 h. The solvent was removed under reduced pressure and the crude product was adsorbed onto silica and purified by column chromatography (cyclohexane/EtOAc 0 - 70% over 37 min) to give *tert*-butyl 3-methyl-4-[3-(thiophene-2-carboxamido)propoxy]-1*H*-indazole-1-carboxylate (**16**) (384 mg, 0.93 mmol), which was slightly contaminated and directly used in the next step without further purification [25].

N-{3-[(3-methyl-1*H*-indazol-4-yl)oxy]propyl}thiophene-2-carboxamide (**17**)

To a solution of **16** (384 mg, 0.93 mmol, 1.00 equiv) in DCM (10 mL), TFA (0.71 mL, 9.24 mmol, 10.0 equiv) was added and the reaction mixture was stirred at room temperature for 16 h. The mixture was washed with aqueous 2 M K_2CO_3 solution and extracted with EtOAc (3 × 50 mL). The combined organic layers were washed with saturated NaCl solution, dried over $MgSO_4$, filtered and evaporated to dryness. The crude product was adsorbed onto silica and purified by column chromatography (cyclohexane/EtOAc 0 - 70% over 27 min) to give *N*-{3-[(3-methyl-1*H*-indazol-4-yl)oxy]propyl}thiophene-2-carboxamide (**17**) (77 mg, 0.25 mmol, 15% over two steps) as a light purple solid.

^{1}H NMR (500 MHz, DMSO-d_6, 300 K): δ (ppm) = 12.47 (s, 1H), 8.58 (t, ^3J = 5.5 Hz, 1H), 7.75–7.72 (m, 2H), 7.18–7.12 (m, 2H), 6.95 (d, ^3J = 8.5 Hz, 1H), 6.43 (d, ^3J = 7.8 Hz, 1H), 4.13 (t, ^3J = 6.0 Hz, 2H), 3.49 (dt, ^3J = 6.6 Hz, ^3J = 6.2 Hz, 2H), 2.58 (s, 3H), 2.06 (quint, ^3J = 6.4 Hz, 2H);^{13}C NMR (125 MHz, DMSO-d_6, 300 K): δ (ppm) = 161.2, 153.6, 143.0, 140.4, 140.1, 130.5, 127.8, 127.7, 127.3, 113.2, 102.5, 99.3, 65.0, 36.2, 29.0, 14.1; HR-MS *m/z* $[M+Na]^+$ calcd. for $C_{16}H_{17}N_3O_2SNa$: 338.0934, found: 338.0927; Purity was determined by qNMR using dimethylsulfone as internal standard: 86.8%; Decomposition at 161 °C.

[4-(2-Chloropyrimidin-4-yl)phenyl]methanone (**19**)

A N_2-flask was charged with 2,4-dichloropyrimidine (500 mg, 3.36 mmol, 1.00 equiv), phenyl[4-(4,4,5,5-tetramethyl-1,3,2-dioxaborolan-2-yl)phenyl]methanone (**18**) (759 mg, 3.36 mmol, 1.00 equiv), K_2CO_3 (929 mg, 6.72 mmol, 2.00 equiv) and 1,4-dioxane/water (4:1, 20 mL). The reaction mixture was degassed with argon for 15 min, $Pd(PPh_3)_4$ (194 mg, 0.17 mmol, 5 mol%) was added and the reaction mixture was stirred at 100 °C for 16 h. After cooling to room temperature, the reaction mixture was filtered over Celite®, water (50 mL) was added and the mixture was extracted with EtOAc (3 × 50 mL). The combined organic layers were washed with saturated NaCl solution, dried over $MgSO_4$, filtered and the solvent was removed under reduced pressure. The crude product was adsorbed onto silica and purified by column chromatography (cyclohexane/EtOAc 0 - 50% over 30 min) to give [4-(2-chloropyrimidin-4-yl)phenyl]methanone (**19**) (352 mg, 1.19 mmol, 35%) as a white solid.

^{1}H NMR (500 MHz, DMSO-d_6, 300 K): δ (ppm) = 8.90 (d, ^3J = 5.3 Hz, 1H), 8.37–8.34 (ps d, ^3J = 8.5 Hz, 2H), 8.24 (d, ^3J = 5.3 Hz, 1H), 7.91–7.88 (ps d, ^3J = 8.7 Hz, 2H), 7.80–7.77 (m, 2H), 7.71 (sm, 1H), 7.61–7.57 (m, 2H); ^{13}C NMR (125 MHz, DMSO-d_6, 300 K): δ (ppm) = 195.2, 165.1, 161.6, 160.6, 139.6, 137.9, 136.6, 133.0, 130.2, 129.7, 128.6, 127.5, 116.7; HR-MS *m/z* $[M+H]^+$ calcd. for $C_{17}H_{11}ClN_2OH$: 295.0633, found: 295.0623; Purity was determined by qNMR using dimethylsulfone as internal standard: 89.7%; mp: 152.6 °C.

{4-[2-(Ethylamino)pyrimidin-4-yl]phenyl}(phenyl)methanone (**20**)

A microwave vessel was charged with **19** (250 mg, 0.85 mmol, 1.00 equiv), ethylamine hydrochloride (139 mg, 1.70 mmol, 2.00 equiv), *N*,*N*-diisopropylethylamine (0.75 mL, 4.24 mmol, 5.00 equiv) and MeCN (5 mL). The reaction mixture was stirred for 1 h at 150 °C under microwave conditions, allowed to cool to room temperature. Then EtOAc (50 mL) was added and the reaction mixture was acidified with aqueous 2 M HCl solution (30 mL). The organic layer was discarded and the aqueous layer was then adjusted to a basic pH value with saturated $NaHCO_3$ solution and extracted with EtOAc (3 × 50 mL). The combined organic layers were washed with saturated NaCl solution, dried over $MgSO_4$, filtered and the crude product was adsorbed onto silica and purified by column chromatography (cyclo-hexa-ne/EtOAc 0 - 50% over 30 min) to give {4-[2-(ethylamino)pyrimidin-4-yl]phenyl}(phenyl)-methanone **(20)** (172 mg, 0.57 mmol, 67%) as a pale yellow solid.

^{1}H NMR (500 MHz, DMSO-d_6, 300 K): δ (ppm) = 8.40 (d, ^3J = 5.3 Hz, 1H), 8.25 (d, ^3J = 8.2 Hz, 2H), 7.85 (ps d, ^3J = 8.5 Hz, 2H), 7.79–7.76 (m, 2H), 7.72–7.67 (sm, 1H), 7.60–7.56 (m, 2H), 7.26 (t, ^3J = 5.7 Hz, 1H), 7.19 (d, ^3J = 5.3 Hz, 1H), 3.40 (sm, 2H), 1.17 (t, ^3J = 7.1 Hz, 3H); ^{13}C NMR (125 MHz, DMSO-d_6, 300 K): δ (ppm) = 195.3, 162.4, 159.2, 140.8, 138.4, 136.9, 132.8, 129.9, 129.6, 128.6, 126.8, 105.8, 35.4, 14.6; HR-MS *m/z* $[M+H]^+$ calcd. for $C_{19}H_{17}N_3OH$: 304.1444, found: 304.1431; Purity was determined by qNMR using dimethylsulfone as internal standard: 99.4%; mp: 137.2 °C.

(2-Amino-6-bromophenyl)methanol (**22**)

To an ice-cooled solution of 2-amino-6-bromobenzoic acid (**21**) (1000 mg, 4.63 mmol, 1.00 equiv) in THF (6 mL) was added dropwise 2.4 M LAH in THF (3.86 mL, 9.26 mmol, 2.00 equiv) under an argon atmosphere at 0 °C, and the reaction was stirred overnight at room temperature. To quench the reaction, Na_2SO_4 * 10 H_2O (200 mg) and EtOAc (2 mL) were added to the reaction mixture and stirred for 0.5 h. The suspension was then filtered and the residue was thoroughly washed with EtOAc. The combined organic layers were washed with saturated aqueous Na-K-tartrate solution (3 x 20 mL), dried over $MgSO_4$, filtered, concentrated, and adsorbed onto silica. The crude product was purified by column chromatography (cyclohexane/EtOAc 10 - 50% over 25 min), yielding **22** (585 mg, 2.90 mmol, 63%) as a yellowish solid.

^{1}H NMR (400 MHz, DMSO-d_6, 300 K): δ (ppm) = 6.88 (dd, ^3J = 8.0 Hz, ^3J = 7.8 Hz, 1H), 6.73 (d, ^3J = 7.8 Hz, 1H), 6.64 (d, ^3J = 8.0 Hz, 1H), 5.32 (s, 2H), 5.03 (t, ^3J = 5.3 Hz, 1H), 4.59 (d, ^3J = 5.3 Hz, 2H); ^{13}C NMR (125 MHz, DMSO-d_6, 300 K): δ (ppm) = 149.5, 129.4, 124.2, 123.1, 119.6, 114.4, 60.4; HR-MS *m/z* $[M+H]^+$ calcd. for $C_7H_8{}^{79}BrNOH$: 201.9862, found: 201.9862; Purity was determined by qNMR using 1,2,4,5-tetrachloro-3-nitro-benzene as internal standard: 100.0%; mp: 93.5 °C.

N-(3-Bromo-2-{[(*tert*-butyldimethylsilyl)oxy]methyl}phenyl)benzamide (**23**)

(2-Amino-6-bromophenyl)methanol (**22**) (1000 mg, 4.95 mmol, 1.00 equiv) was dissolved in DMF (14 mL) and then imidazole (607 mg, 8.91 mmol, 1.80 equiv) was added. TBSCl (1115 mg, 7.43 mmol, 1.50 equiv) was added after cooling the solution to 0 °C. The reaction was stirred for 4 h at room temperature, and after adding water (20 mL), it was extracted with EtOAc (25 mL). The organic layer was washed with aqueous 5% LiCl solution (3 x 20 mL), dried over $MgSO_4$, filtered, adsorbed onto silica, and purified by column chromatography (cyclohexane/EtOAc 0 - 30% over 15 min), yielding 3-bromo-2-{[(tert-butyldimethylsilyl)oxy]methyl}aniline (1500 mg, 4.75 mmol, 96%) as a yellow liquid.
^{1}H NMR (400 MHz, DMSO-d_6, 300 K): δ (ppm) = 6.90 (dd, ^3J = 8.0 Hz, ^3J = 7.8 Hz, 1H), 6.75 (d, ^3J = 7.8 Hz, 1H), 6.67 (d, ^3J = 8.0 Hz, 1H), 5.26 (s, 2H), 4.81 (s, 2H), 0.86 (s, 9H), 0.07 (s, 6H); ^{13}C NMR (125 MHz, DMSO-d_6, 300 K): δ (ppm) = 149.5, 129.8, 123.7, 121.9, 119.7, 114.5, 62.5, 25.7, 17.8, -5.3; HR-MS *m/z* $[M+Na]^+$ calcd. for $C_{13}H_{22}{}^{79}BrNOSiNa$: 338.0546, found: 338.0544; Purity was determined by qNMR using 1,2,4,5-tetrachloro-3-nitro-benzene as internal standard: 99.9%.

To a solution of 3-bromo-2-{[(tert-butyldimethylsilyl)oxy]methyl}aniline (700 mg, 2.21 mmol, 1.00 equiv) in DCM (11 mL), benzoyl chloride (0.28 mL, 2.43 mmol, 1.10 equiv) was added. The solution was then cooled to 0 °C, whereupon triethylamine (0.49 mL, 3.54 mmol, 1.60 equiv) was added and the reaction mixture was stirred for 5 h at room temperature, then washed with water (3 x 15 ml), dried over $MgSO_4$, filtered, concentrated, adsorbed onto silica, and purified by column chromatography (cyclohexane/EtOAc 0 - 10% over 20 min), yielding **23** (840 mg, 2.00 mmol, 90%) as a white solid.
^{1}H NMR (400 MHz, DMSO-d_6, 300 K): δ (ppm) = 10.01 (s, 1H), 7.95 - 7.92 (m, 2H), 7.84 (dd, ^3J = 8.0 Hz, ^4J = 1.1 Hz, 1H), 7.64 - 7.60 (m, 1H), 7.56 - 7.52 (m, 2H), 7.49 (dd, ^3J = 8.2 Hz, ^4J = 1.1 Hz, 1H), 7.31 (dd, ^3J = 8.0 Hz, ^3J = 8.2 Hz, 1H), 4.98 (s, 2H), 0.79 (s, 9H), 0.03 (s, 6H); ^{13}C NMR (125 MHz, DMSO-d_6, 300 K): δ (ppm) = 165.1, 138.9, 134.2, 131.9, 131.7, 129.7, 129.3, 128.6, 127.2, 124.0, 123.5, 62.2, 25.6, 17.8, -5.4; HR-MS *m/z* $[M+H]^+$ calcd. for $C_{20}H_{26}{}^{79}BrNO_2SiH$: 420.0989, found: 420.0978; Purity was determined by qNMR using dimethylsulfone as internal standard: 98.5%; mp: 106.2 °C.

N-[3-(2-Aminopyridin-4-yl)-2-(hydroxymethyl)phenyl]benzamide (**24**)

N-(3-Bromo-2-{[(tert-butyldimethylsilyl)oxy]methyl}phenyl)benzamide (**23**) (400 mg, 0.95 mmol, 1.00 equiv), 4-(4,4,5,5-tetramethyl-1,3,2-dioxaborolan-2-yl)pyridine-2-amine (251 mg, 1.14 mmol, 1.20 equiv), Na_2CO_3 (302 mg, 2.85 mmol, 3.00 equiv) and $Pd(PPh_3)_4$ (110 mg, 0.10 mmol, 10 mol%) were placed in a microwave vessel under an argon atmosphere. After adding DME/water (2:1, 5 mL), the reaction was stirred for 1 h at 130 °C under microwave conditions. The reaction mixture was concentrated, adsorbed onto silica, and purified by column chromatography (DCM/MeOH 0 - 10% over 30 min), which resulted in two fractions, one with and one without a protecting group. The latter

fraction was concentrated, dissolved in aqueous 0.5 M HCl solution (20 mL) and washed with EtOAc (3 x 15 mL). The aqueous phase was then neutralized with aqueous 2 M NaOH solution and the resulting suspension was filtered, yielding **24** (73 mg, 0.23 mmol, 24%) as a light beige solid.

^{1}H NMR (400 MHz, DMSO-d_6, 300 K): δ (ppm) = 10.35 (s, 1H), 8.11 (d, ^{3}J = 8.2 Hz, 1H), 7.96 - 7.94 (m, 3H), 7.65 - 7.56 (m, 3H), 7.40 (dd, ^{3}J = 7.9 Hz, ^{3}J = 8.1 Hz, 1H), 7.03 (d, ^{3}J = 7.8 Hz, 1H), 6.52 (d, ^{3}J = 5.0 Hz, 1H), 6.41 (s, 1H), 5.99 (s, 2H), 5.84 (t, ^{3}J = 3.89 Hz, 1H), 4.58 (d, ^{3}J = 3.89 Hz, 1H); ^{13}C NMR (125 MHz, DMSO-d_6, 300 K): δ (ppm) = 164.7, 159.8, 149.0, 147.5, 140.4, 138.5, 134.5, 131.9, 128.8, 128.6, 127.7, 127.0, 125.0, 122.2, 112.9, 108.0, 58.4; HR-MS *m/z* [M+H]$^{+}$ calcd. for $C_{19}H_{17}N_3O_2H$: 320.1394, found: 320.1393; Anal. calcd. for $C_{19}H_{17}N_3O_2$ x 2 H_2O : C: 64.21, H 5.96, N 11.82, found: C 64.18, H 5.74 N 11.78; mp: 154.0 °C.

{4-[7*H*-Pyrrolo(2,3-*d*)pyrimidin-4-yl]phenyl}(phenyl)methanone (26)

A N_2-flask was charged with 4-bromo-7*H*-pyrrolo[2,3-*d*]pyrimidine (**25**) (500 mg, 2.52 mmol, 1.00 equiv), phenyl[4-(4,4,5,5-tetramethyl-1,3,2-dioxaborolan-2-yl)phenyl]methanone (**18**) (570 mg, 2.52 mmol, 1.00 equiv), K_2CO_3 (697 mg, 5.04 mmol, 2.00 equiv) and 1,4-dioxane/water (4:1, 13 mL). The reaction mixture was degassed with argon for 15 min, $Pd(PPh_3)_4$ (146 mg, 0.13 mmol, 5 mol%) was added and the reaction mixture was stirred at 100 °C for 18 h. After cooling to room temperature, the reaction mixture was filtered over Celite®, water (50 mL) was added and the mixture was extracted with EtOAc (3 × 50 mL). The combined organic layers were washed with saturated NaCl solution, dried over $MgSO_4$, filtered and the solvent was removed under reduced pressure. The crude product was adsorbed onto silica and purified by column chromatography (cyclohexane/EtOAc 0 - 70% over 30 min) to give {4-[7*H*-pyrrolo(2,3-*d*)pyrimidin-4-yl]phenyl}(phenyl)methanone (**26**) (120 mg, 0.40 mmol, 16%) as a white solid.

^{1}H NMR (500 MHz, DMSO-d_6, 300 K): δ (ppm) = 12.34 (br s, 1H), 8.90 (s, 1H), 8.34 (ddd, ^{3+5}J = 8.5 Hz, ^{4}J = 1.8 Hz, ^{4}J = 1.8 Hz, 2H), 7.94 (ddd, ^{3+5}J = 8.5 Hz, ^{4}J = 1.8 Hz, ^{4}J = 1.8 Hz, 2H), 7.82 (m, 1H), 7.81 (m, 1H), 7.72–7.68 (m, 2H), 7.61–7.57 (m, 2H), 6.96 (d, ^{4}J = 3.7 Hz, 1H); ^{13}C NMR (125 MHz, DMSO-d_6, 300 K): δ (ppm) = 195.4, 154.1, 152.9, 150.9, 141.6, 137.8, 136.9, 132.8, 130.1, 129.6, 128.6, 128.3, 114.8, 99.8; HR-MS *m/z* [M+H]$^{+}$ calcd. for $C_{19}H_{13}N_3OH$: 300.1131, found: 300.1122; Purity was determined by qNMR using dimethylsulfone as internal standard: 95.8%; mp: 204.3 °C.

1-(3-Bromobenzyl)-1*H*-1,2,4-triazole (29)

1-Bromo-3-(bromomethyl)benzene (**27**) (2.00 g, 8.00 mmol, 1.00 equiv), 1*H*-1,2,4-triazole (**28**) (0.83 g, 12.00 mmol, 1.50 equiv), K_2CO_3 (1.11 g, 8.00 mmol, 1.00 equiv) and potassium iodide (0.08 g, 0.48 mmol, 6 mol%) were suspended in acetone (30 mL). The reaction mixture was stirred at 55 °C for 16 h, allowed to cool to room temperature, and then EtOAc (100 mL) was added. The organic phase was

washed with H_2O, aqueous 1 M NaOH, and saturated NaCl solution, dried over $MgSO_4$, filtered, and the crude product was adsorbed onto silica and purified by column chromatography (cyclohexane/EtOAc 0 - 100% over 40 min) to give 1-(3-bromobenzyl)-1*H*-1,2,4-triazole (**29**) (1.30 g, 5.46 mmol, 68%) as a pale yellow oil [26].

^{1}H NMR (500 MHz, DMSO-d_6, 300 K): δ (ppm) = 8.67 (s, 1H), 7.99 (s, 1H), 7.53–7.49 (m, 2H), 7.33 (dd, ^3J = 7.6 Hz, ^3J = 7.8 Hz, 1H), 7.29–7.26 (m, 1H), 5.43 (s, 2H); ^{13}C NMR (125 MHz, DMSO-d_6, 300 K): δ (ppm) = 151.9, 144.4, 138.9, 130.8, 130.8, 130.6, 126.9, 121.7, 51.2; HR-MS *m/z* $[M+H]^+$ calcd. for $C_9H_8{}^{79}BrN_3H$: 237.9974, found: 237.9967.

4-{3-[(1*H*-1,2,4-triazol-1-yl)methyl]phenyl}-1*H*-pyrrolo[2,3-*b*]pyridine (**30**)

A N_2-flask was charged with 1-(3-bromobenzyl)-1*H*-1,2,4-triazole (**29**) (500 mg, 2.10 mmol, 1.00 equiv), 4-(4,4,5,5-tetramethyl-1,3,2-dioxaborolan-2-yl)-1*H*-pyrrolo[2,3-*b*]pyridine (513 mg, 2.10 mmol, 1.00 equiv), K_3PO_4 (1.34 g, 6.30 mmol, 3.00 equiv) and 1,4-dioxane/water (4:1, 30 mL). The reaction mixture was degassed with argon for 15 min, Pd(dppf)Cl_2 * DCM (86 mg, 0.11 mmol, 5 mol%) was added and the reaction mixture was stirred at 110 °C for 18 h. After cooling to room temperature, the reaction mixture was filtered over Celite®, water (100 mL) was added and the mixture was extracted with EtOAc (3 × 100 mL). The combined organic layers were washed with saturated NaCl solution, dried over $MgSO_4$, filtered and the solvent was removed under reduced pressure. The crude product was adsorbed onto silica and purified by column chromatography (DCM/MeOH 0 - 10% over 20 min) to give 4-{3-[(1*H*-1,2,4-triazol-1-yl)methyl]phenyl}-1*H*-pyrrolo[2,3-*b*]pyridine (**30**) (390 mg, 1.42 mmol, 68%) as a grey solid [27].

^{1}H NMR (500 MHz, DMSO-d_6, 300 K): δ (ppm) = 11.79 (s, 1H), 8.73 (s, 1H), 8.29 (d, ^3J = 4.8 Hz, 1H), 8.01 (s, 1H), 7.71–7.68 (m, 2H), 7.56–7.52 (m, 2H), 7.38 (d, ^3J = 7.6 Hz, 1H), 7.15 (d, ^3J = 4.8 Hz, 1H), 6.55 (m, 1H), 5.55 (s, 2H); ^{13}C NMR (125 MHz, DMSO-d_6, 300 K): δ (ppm) = 151.8, 149.1, 144.3, 142.9, 139.7, 138.8, 137.1, 129.3, 127.7, 127.7, 127.6, 126.7, 117.1, 114.0, 98.8, 51.9; HR-MS *m/z* $[M+H]^+$ calcd. for $C_{16}H_{13}N_5H$: 276.1244, found: 276.1232; Purity was determined by qNMR using dimethylsulfone as internal standard: 97.9%; Decomposition at 146 °C.

3-[(3-Morpholinopropyl)amino]benzamide (**33**)

A N_2-flask was charged with K_3PO_4 (796 mg, 3.75 mmol, 1.50 equiv) and dried *in vacuo* at 140 °C. 3-Bromobenzamide (**31**) (500 mg, 2.50 mmol, 1.00 equiv), Pd(dba)$_2$ (58 mg, 0.10 mmol, 4 mol%), tBuBrettPhos (73 mg, 0.15 mmol, 6 mol%), 3-morpholinopropan-1-amine (**32**) (0.37 mL, 2.50 mmol, 1.00 equiv) and 1,4-dioxane (12.5 mL) were added and the reaction mixture was stirred at 100 °C for 22 h. After cooling to room temperature, the reaction mixture was filtered over Celite®, water (50 mL) was added and the mixture was extracted with EtOAc (3 × 50 mL). The combined organic layers were

washed with saturated NaCl solution, dried over $MgSO_4$, filtered and the solvent was removed under reduced pressure. The crude product was adsorbed onto silica and purified by column chromatography (DCM/MeOH 0 - 10% over 43 min) to give 3-[(3-morpholinopropyl)amino]benzamide (**33**) (120 mg, 0.46 mmol, 18%) as an off-white solid.

^{1}H NMR (500 MHz, DMSO-d_6, 300 K): δ (ppm) = 7.73 (br s, 1H), 7.12–7.08 (m, 2H), 7.02–6.99 (m, 2H), 6.69–6.67 (dd, ^3J = 8.0 Hz, ^4J = 1.8 Hz, 1H), 5.72 (br s, 1H), 3.58 (sm, 4H), 3.06 (t, ^3J = 6.6 Hz, 2H), 2.37–2.36 (m, 6H), 1.69 (quintet, ^3J = 6.9 Hz, 2H); ^{13}C NMR (125 MHz, DMSO-d_6, 300 K): δ (ppm) = 168.6, 148.9, 135.1, 128.5, 114.9, 114.5, 110.6, 66.1, 56.1, 53.4, 41.0, 25.4; HR-MS *m/z* $[M+H]^+$ calcd. for $C_{14}H_{21}N_3O_2H$: 264.1707, found: 264.1694; Purity was determined by qNMR using 1,2,4,5-tetrachloro-3-nitro-benzene as internal standard: 99.9%; mp: 149.7 °C.

tert-Butyl 4-[(4-bromophenyl)carbamoyl]piperidine-1-carboxylate (**36a**)

To an ice-cold solution of 1-(*tert*-butoxycarbonyl)piperidine-4-carboxylic acid (**34**) (1000 mg, 4.36 mmol, 1.00 equiv), 4-bromo-aniline (**35a**) (750 mg, 4.36 mmol, 1.00 equiv), and DMAP (692 mg, 5.66 mmol, 1.30 equiv) in DCM (20 mL), 1-ethyl-3-(3-dimethylaminopropyl)carbodiimide*HCl (1086 mg, 5.66 mmol, 1.30 equiv) was slowly added, the reaction mixture was allowed to warm to room temperature and then stirred for 24 h. The reaction was quenched by adding water (10 mL) and the phases were separated. The organic phase was washed with water (2 x 15 mL) and saturated $NaHCO_3$ solution (2 x 15 mL), dried over $MgSO_4$, filtered, concentrated, and adsorbed onto silica. Purification by column chromatography (cyclohexane/EtOAc 0 - 70% over 35 min) yielded **36a** (1376 mg, 3.98 mmol, 82%) as a white solid.

^{1}H NMR (400 MHz, DMSO-d_6, 300 K): δ (ppm) = 11.01 (br s, 1H), 7.57 (d, ^3J = 8.9 Hz, 2H), 7.46 (d, ^3J = 8.9 Hz, 2H), 3.99 (d, ^2J = 12.8 Hz, 2H), 2.90 - 2.63 (m, 2H), 2.55 - 2.45 (m, 1H; partially overlapping with the DMSO signal), 1.77 (dd, ^2J = 13.0 Hz, ^3J = 2.3 Hz, 2H), 1.57 - 1.43 (m, 2H), 1.40 (s, 9H); ^{13}C NMR (100 MHz, DMSO-d_6, 300 K): δ (ppm) = 173.1, 153.8, 138.6, 131.4, 121.0, 114.5, 78.6, 42.6, 28.0; HR-MS *m/z* $[M+Na]^+$ calcd. for $C_{17}H_{23}{}^{79}BrN_2O_3Na$: 405.0784, found: 405.0780.

tert-Butyl-4-[(4-bromo-3-chlorophenyl)carbamoyl]piperidine-1-carboxylate (**36b**)

To an ice-cold solution of 1-(*tert*-butoxycarbonyl)piperidine-4-carboxylic acid (**34**) (1000 mg, 4.36 mmol, 1.00 equiv), 4-bromo-3-chloroaniline (**35b**) (900 mg, 4.36 mmol, 1.00 equiv) and DMAP (692 mg, 5.66 mmol, 1.30 equiv) in DCM (20 mL), 1-ethyl-3-(3-dimethylaminopropyl)carbodiimide*HCl (1086 mg, 5.66 mmol, 1.30 equiv) was slowly added, the reaction mixture was allowed to warm to room temperature and then stirred overnight. The reaction was quenched by adding water (10 mL) and the phases were separated. The organic phase was washed with water (2 x 15 mL) and saturated $NaHCO_3$ solution (2 x 15 mL), dried over $MgSO_4$, filtered, concentrated, and adsorbed onto silica.

Purification by column chromatography (cyclohexane/EtOAc 0 - 70% over 35 min) yielding **36b** (1664 mg, 3.98 mmol, 91%) as a white solid.

^{1}H NMR (400 MHz, DMSO-d_6, 300 K): δ (ppm) = 10.17 (s, 1H), 8.00 (d, ^{4}J = 2.5 Hz, 1H), 7.66 (d, ^{3}J = 8.7 Hz, 1H), 7.42 (dd, ^{3}J = 8.7 Hz, ^{4}J = 2.5 Hz, 1H), 3.98 (d, ^{2}J = 12.8 Hz, 2H), 2.81 - 2.74 (m, 2H), 2.53 - 2.46 (m, 1H), 1.77 (dd, ^{2}J = 13.0 Hz, ^{3}J = 2.3 Hz, 2H), 1.56 - 1.43 (m, 2H), 1.40 (s, 9H); ^{13}C NMR (125 MHz, DMSO-d_6, 300 K): δ (ppm) = 173.4, 153.8, 139.9, 133.7, 132.9, 120.2, 119.3, 114.1, 78.6, 43.0, 42.6, 28.0, 28.0; HR-MS *m/z* [M+Na]$^+$ calcd. for $C_{17}H_{22}{}^{79}BrClN_2O_3Na$: 439.0395, found: 439.0400; Purity was determined by qNMR using 1,2,4,5-tetrachloro-3-nitro-benzene as internal standard: 99.9%; mp: 158.6 °C.

tert-Butyl 4-{[4-(4,4,5,5-tetramethyl-1,3,2-dioxaborolan-2-yl)phenyl]carbamoyl}-piperidine-1-carboxylate (**37a**)

KOAc (922 mg, 9.39 mmol, 3.00 equiv) was added to a thoroughly dried flask, the flask was evacuated, refilled with argon, then *tert*-butyl 4-[(4-bromophenyl)carbamoyl]piperidine-1-carboxylate (**36a**) (1200 mg, 3.13 mmol, 1.00 equiv) and 1,4-dioxane (31 mL) were added, and the suspension was degassed with argon and stirred for 10 minutes. After adding *bis*-(pinacolato)-diborane (1194 mg, 4.70 mmol, 1.50 equiv) and Pd(dppf)Cl_2 * DCM (256 mg, 0.31 mmol, 10 mol%), the reaction was stirred for 3 h at 100 °C, cooled to room temperature, then the solvent was removed and the residue was purified directly by column chromatography (cyclohexane/EtOAc 10 - 40% over 25 min). The product fractions were combined and the solvent was removed. The crude product was suspended in MTBE, triturated in an ultrasonic bath, and the precipitate was collected. This process was repeated twice. **37a** was obtained as a white solid (910 mg, 2.11 mmol, 68%), which was still contaminated with traces of *bis*-(pinacolato)-diborane.

^{1}H NMR (400 MHz, DMSO-d_6, 300 K): δ (ppm) = 10.00 (s, 1H), 7.64 – 7.57 (m, 4H), 3.99 (d, ^{2}J = 12.8 Hz, 2H), 2.85 - 2.69 (m, 2H), 2.57 - 2.51 (m, 1H; overlapping with the DMSO signal), 1.77 (dd, ^{2}J = 13.0 Hz, ^{3}J = 2.5 Hz, 2H), 1.57 - 1.42 (m, 2H), 1.41 (s, 9H), 1.29 (s, 12H); ^{13}C NMR (100 MHz, DMSO-d_6, 300 K): δ (ppm) = 173.2, 153.8, 142.0, 135.1, 118.1, 83.3, 78.6, 42.6, 28.0, 24.7; HR-MS *m/z* [M+Na]$^+$ calcd. for $C_{23}H_{35}BN_2O_5Na$: 453.2535, found: 453.2529.

tert-Butyl-4-{[3-chloro-4-(4,4,5,5-tetramethyl-1,3,2-dioxaborolan-2-yl)phenyl]-carbamoyl}-piperidine-1-carboxylate (**37b**)

KOAc (986 mg, 10.05 mmol, 3.00 equiv) was added to a thoroughly dried flask, the flask was evacuated, refilled with argon, then *tert*-butyl 4-[(4-bromo-3-chlorophenyl)carbamoyl]piperidine-1-carboxylate) (**36b**) (1400 mg, 3.35 mmol, 1.00 equiv) and 1,4-dioxane (33.50 mL) were added, and the suspension was degassed with argon and stirred for 10 minutes. After adding *bis*-(pinacolato)-diborane (1277 mg, 5.03 mmol, 1.50 equiv) and Pd(dppf)Cl_2 * DCM (278 mg, 0.34 mmol, 10 mol%), the reaction was stirred

for 3 h at 100 °C, cooled to room temperature, then the solvent was removed and the residue was purified directly by column chromatography (cyclohexane/EtOAC 10 - 40 % over 25 min). The product fractions were combined and the solvent was removed. The crude product was suspended in MTBE, triturated in an ultrasonic bath, and the precipitate was collected. This process was repeated twice. (**37b**) was obtained as a white solid (1493 mg, 3.21 mmol, 96%), which was still contaminated with traces of *bis*-(pinacolato)-diborane and was used directly without further purification.

^{1}H NMR (400 MHz, DMSO-d_6, 300 K): δ (ppm) = 10.16 (s, 1H), 7.79 (d, ^{4}J = 1.8 Hz, 1H), 7.58 (d, ^{3}J = 8.2 Hz, 1H), 7.48 (dd, ^{3}J = 8.2 Hz, ^{4}J = 1.8 Hz, 1H), 3.98 (d, ^{2}J = 12.8 Hz, 2H), 2.80 - 2.74 (m, 2H), 2.54 - 2.47 (m, 1H), 1.77 (dd, ^{2}J = 13.0 Hz, ^{3}J = 2.1 Hz, 2H), 1.56 - 1.43 (m, 2H), 1.40 (s, 9H), 1.29 (s, 12H); ^{13}C NMR (125 MHz, DMSO-d_6, 300 K): δ (ppm) = 173.5, 153.8, 142.7, 138.9, 137.2, 119.1, 116.5, 83.6, 78.6, 43.0, 42.6, 28.0, 24.5; HR-MS *m/z* [M+Na]$^+$ calcd. for $C_{23}H_{34}BClN_2O_5Na$: 487.2157, found: 487.2146.

N-(4-(1*H*-Pyrrolo[2,3-*b*]pyridin-4-yl)phenyl)piperidine-4-carboxamide dihydrochloride (**38a**)

tert-Butyl 4-{[4-(4,4,5,5-tetramethyl-1,3,2-dioxaborolan-2-yl)phenyl]carbamoyl}piperidine-1-carboxylate (crude **37a**) (1291 mg, 3.00 mmol, 1.00 equiv), 4-bromo-7-azaindole (650 mg, 3.30 mmol, 1.10 equiv), Na_2CO_3 (954 mg, 9.00 mmol, 3.00 equiv), and $Pd(PPh_3)_4$ (347 mg, 0.30 mmol, 10 mol%) were suspended in a microwave vessel in a DME/water (2:1, 12 mL). The vessel was then flushed with argon, sealed airtight, and stirred for 1 h at 130 °C under microwave conditions. After cooling to room temperature, the reaction mixture was filtered over Celite® and the residue thoroughly washed with MeOH. The filtrate was concentrated under reduced pressure, adsorbed onto silica, and purified by column chromatography (DCM/MeOH 0 - 10% over 30 min). The corresponding fractions were concentrated under reduced pressure, the remaining residue was resuspended in 1,4-dioxane (30 mL), and after adding 4 M HCl in 1,4-dioxane (3.85 mL, 15.40 mmol, 10.00 equiv), the reaction mixture was stirred at room temperature for 24 h. Following the addition of water (50 mL), the aqueous phase was washed with DCM (3 x 50 mL). After adjusting to a basic pH value by adding aqueous 2 M NaOH, the aqueous phase was extracted with EtOAc (3 x 70 mL). The combined organic phases were washed with a saturated NaCl solution (20 mL), dried over $MgSO_4$, filtered, and concentrated to a volume of approximately 25 mL under reduced pressure. The product was precipitated as hydrochloride by adding 2 M HCl in diethyl ether. The precipitate was filtered off, washed with EtOAc (2 x 5 mL), and dried under vacuum to give **38a** as its dihydrochloride (794 mg, 2.23 mmol, 74%) as a white solid.

^{1}H NMR (400 MHz, DMSO-d_6, 300 K): δ (ppm) = 12.55 (s, 1H), 10.52 (s, 1H), 9.15 (br s, 1H), 8.80 (br s, 1H), 8.41 (d, ^{3}J = 5.7 Hz, 1H), 7.88 (d, ^{3}J = 8.9 Hz, 2H), 7.83 (d, ^{3}J = 8.9 Hz, 2H), 7.70 (dd, ^{3}J = 3.4 Hz, ^{3}J = 2.5 Hz, 1H), 7.46 (d, ^{3}J = 5.7 Hz, 1H), 6.85 (dd, ^{3}J = 3.7 Hz, ^{3}J = 1.6 Hz, 1H), 3.33 (d, ^{3}J = 12.6 Hz, 2H), 2.95 (d, ^{3}J = 12.1 Hz, 1H), 2.90 (d, ^{3}J = 12.4 Hz, 1H), 2.75 (sm, 1H), 2.13-1.95 (m, 2H), 1.95-1.77 (m, 2H); ^{13}C

NMR (100 MHz, DMSO-d_6, 300 K): δ (ppm) = 172.7, 146.8, 141.2, 140.6, 135.2, 130.6, 129.5, 129.3, 120.9, 119.6, 114.0, 101.5, 42.3, 40.5, 25.0; HR-MS *m/z* $[M+2H]^{2+}$ calcd. for $C_{19}H_{20}N_4OH_2$: 161.0891, found: 161.0886; Anal. calcd. for $C_{19}H_{20}N_4O$*2HCl x 1.0 H_2O : C 55.48, H 5.88, N 13.62, found: C 55.06, H 5.94, N 13.40; mp: decomposition > 314.5 °C.

N-[3-Chloro-4-(1*H*-pyrrolo[2,3-*b*]pyridin-4-yl)phenyl]piperidine-4-carboxamide (**38b**)

tert-Butyl-4-{[3-chloro-4-(4,4,5,5-tetramethyl-1,3,2-dioxaborolan-2-yl)phenyl]-carbamoyl}-piperidine-1-carboxylate (crude **37b**) (1395 mg, 3.00 mmol, 1.00 equiv), 4-bromo-7-azaindole (650 mg, 3.30 mmol, 1.10 equiv), Na_2CO_3 (954 mg, 9.00 mmol, 3.00 equiv), and $Pd(PPh_3)_4$ (347 mg, 0.30 mmol, 10 mol%) were suspended in a microwave vessel in a DME/water (2:1, 12 mL). The vessel was then flushed with argon, sealed airtight, and stirred for 1 h at 130 °C under microwave conditions. After cooling to room temperature, the reaction mixture was filtered over Celite® and the residue thoroughly washed with MeOH. The filtrate was concentrated under reduced pressure, adsorbed onto silica, and purified by column chromatography (DCM/MeOH 0 - 10 % over 30 min). The corresponding fractions were concentrated under reduced pressure, the remaining residue was resuspended in 1,4-dioxane (30 mL), and after adding 4 M HCl in 1,4-dioxane (3.85 mL, 15.40 mmol, 10.00 equiv), the reaction mixture was stirred at room temperature for 24 h. Following the addition of water (50 mL), the aqueous phase was washed with DCM (3 x 50 mL) and then adjusted to a basic pH value with aqueous 2 M NaOH. The resulting precipitate was filtered off and washed with water (2 x 5 mL). After drying under vacuum, **38b** (843 mg, 2.38 mmol, 79% over two steps) was obtained as a slightly yellowish solid.

^{1}H NMR (400 MHz, DMSO-d_6, 300 K): δ (ppm) = 11.75 (s, 1H), 10.13 (s, 1H), 8.26 (d, ^3J = 4.8 Hz, 1H), 8.02 (d, ^3J = 2.1 Hz, 1H), 7.61 (dd, ^3J = 8.3, ^4J = 1.9 Hz, 1H), 7.48 (d, ^3J = 3.2 Hz, 1H), 7.43 (d, ^3J = 8.2 Hz, 1H), 7.02 (d, ^3J = 4.8 Hz, 1H), 6.20 (d, ^3J = 3.4 Hz, 1H), 3.08-2.89 (m, 2H), 2.59-2.30 (m, 4H, partially overlapping with the DMSO signal), 1.80-1.61 (m, 2H), 1.60-1.40 (m, 2H); ^{13}C NMR (100 MHz, DMSO-d_6, 300 K): δ (ppm) = 174.3, 148.7, 142.2, 140.4, 138.2, 131.5, 131.4, 131.3, 126.4, 119.7, 118.6, 117.7, 116.1, 99.2, 45.5, 43.8, 29.3; HR-MS *m/z* $[M+H]^+$ calcd. for $C_{19}H_{19}ClN_4OH$: 355.1320, found: 355.1306; Anal. calcd. for $C_{19}H_{19}ClN_4O$ x 1.25 H_2O : C: 60.47, H 5.74, N 14.85, found: C 60.25, H 5.69 N 14.48; mp: 233.8 °C.

N-[4-(7*H*-Pyrrolo[2,3-*d*]pyrimidin-4-yl)phenyl]piperidine-4-carboxamide Hydrochloride (**38c**)

Two microwave vessels were each charged with *tert*-butyl 4-{[4-(4,4,5,5-tetramethyl-1,3,2-dioxaborolan-2-yl)phenyl]carbamoyl}piperidine-1-carboxylate (**37a**) (550 mg, 1.28 mmol, 1.00 equiv), 4-bromo-7*H*-pyrrolo[2,3-*d*]pyrimidine (279 mg, 1.41 mmol, 1.10 equiv), Na_2CO_3 (407 mg, 3.84 mmol, 3.00 equiv) and $Pd(PPh_3)_4$ (148 mg, 0.13 mmol, 10 mol%). After addition of a DME/water (2:1, 6.5 mL),

the vessels were flushed with argon, sealed airtight, and stirred for 1 h at 130 °C under microwave conditions. After cooling to room temperature, the reaction mixtures were combined, filtered over Celite® and the residue thoroughly washed with MeOH. The filtrate was concentrated under reduced pressure, adsorbed onto silica, and purified by column chromatography (DCM/MeOH 0 - 10% over 30 min), yielding 814 mg of the intermediate. 800 mg (1.90 mmol) were resuspended in 76 mL of 1,4-dioxane, and after adding 4 M HCl in 1,4-dioxane (4.75 mL, 19.00 mmol, 10.00 equiv), the reaction mixture was stirred at room temperature for 24 h and the precipitate was filtered off and washed with 1,4-dioxane. The crude product was purified in portions (approx. 250 mg per separation) by preparative HPLC (water/MeOH 5 - 95% over 1 h). **38c** (372 mg, 1.03 mmol, 54%) was obtained as its hydrochloride in form of a white solid.

^{1}H NMR (400 MHz, DMSO-d_6, 300 K): δ (ppm) = 12.28 (s, 1H), 10.51 (s, 1H), 9.18 (br s, 1H), 8.85, (br s, 1H), 8.80 (s, 1H), 8.17 (d, ^{3}J = 8.7 Hz, 2H), 7.86 (d, ^{3}J = 8.7 Hz, 2H), 7.64 (dd, ^{3}J = 3.0 Hz, ^{3}J = 2.9 Hz, 1H), 6.93 (dd, ^{3}J = 3.4 Hz, ^{3}J = 1.6 Hz, 1H), 3.33 (d, ^{2}J = 12.3 Hz, 2H), 2.92 (sm, 2H), 2.76 (sm, 1H), 2.01 (d, ^{2}J = 11.4 Hz, 2H), 1.88 (sm, 2H); ^{13}C NMR (100 MHz, DMSO-d_6, 300 K): δ (ppm) = 172.5, 154.7, 152.5, 150.5, 141.0, 132.1, 129.2, 127.6, 119.1, 114.0, 100.2, 42.3, 39.9, 25.0; HR-MS *m/z* $[M+H]^+$ calcd. for $C_{18}H_{19}N_5OH$: 322.1662, found: 322.1648; Anal. calcd. for $C_{18}H_{19}N_5O$ * HCl x 1.75 H_2O : C 55.52, H 6.08, N 17.99, found: C 55.42, H 5.86, N 17.82; mp: decomposition > 345.4 °C.

N-[3-Chloro-4-(7*H*-pyrrolo[2,3-*d*]pyrimidin-4-yl)phenyl]piperidine-4-carboxamide Hydrochloride (**38d**)

Two microwave vessels were each charged with *tert*-butyl-4-{[3-chloro-4-(4,4,5,5-tetramethyl-1,3,2-dioxaborolan-2-yl)phenyl]-carbamoyl}piperidine-1-carboxylate (**37b**) (600 mg, 1.29 mmol, 1.00 equiv), 4-bromo-7*H*-pyrrolo[2,3-*d*]pyrimidine (281 mg, 1.42 mmol, 1.10 equiv), Na_2CO_3 (410 mg, 3.87 mmol, 3.00 equiv) and $Pd(PPh_3)_4$ (149 mg, 0.13 mmol, 10 mol%). After addition of a DME/water (2:1, 6.5 mL), the vessels were flushed with argon, sealed airtight, and stirred for 1 h at 130 °C under microwave conditions. After cooling to room temperature, the reaction mixtures were combined, filtered over Celite® and the residue thoroughly washed with MeOH. The filtrate was concentrated under reduced pressure, adsorbed onto silica, and purified by column chromatography (DCM/MeOH 0-10% over 30 min), yielding *tert*-Butyl-4-{[3-chloro-4-(7*H*-pyrrolo[2,3-*d*]pyrimidin-4-yl)phenyl]carbamoyl}-piperidine-1-carboxylate (396 mg, 0.87 mmol, 67%) as a slightly yellowish solid.

^{1}H NMR (400 MHz, DMSO-d_6, 300 K): δ (ppm) = 12.20 (s, 1H), 10.26 (s, 1H), 8.82 (s, 1H), 8.02 (d, ^{4}J = 2.1 Hz, 1H), 7.64 (dd, ^{3}J = 8.5 Hz, ^{4}J = 2.1 Hz, 1H), 7.58 (d, ^{3}J = 3.4 Hz, 1H), 7.54 (d, ^{3}J = 8.5 Hz, 1H), 6.36 (d, ^{3}J = 3.3 Hz, 1H), 4.01 (d, ^{2}J = 12.8 Hz, 2H), 2.84 - 2.77 (m, 2H), 2.56 (tt, ^{3}J = 11.4 Hz, ^{3}J = 3.7 Hz, 1H), 1.82 (dd, ^{2}J = 13.3 Hz, ^{3}J = 2.7 Hz, 2H), 1.57 - 1.46 (m, 2H), 1.41 (s, 9H); ^{13}C NMR (125 MHz, DMSO-d_6, 300 K): δ (ppm) = 173.6, 155.5, 153.8, 151.7, 150.6, 141.0, 131.8, 131.4, 131.1, 127.4, 119.7, 117.5,

116.6, 99.9, 78.6, 42.8, 42.7, 28.0; HR-MS *m/z* $[M+H]^+$ calcd. for $C_{23}H_{26}ClN_5O_3Na$: 478.1616, found: 478.1620.

tert-Butyl-4-{[3-chloro-4-(7*H*-pyrrolo[2,3-*d*]pyrimidin-4-yl)phenyl]carbamoyl}-piperidine-1-carboxylate (700 mg, 1.54 mmol, 1.00 equiv) was suspended in 1,4-dioxane (62 mL). After adding 4 M HCl in 1,4-dioxane (3.85 mL, 15.40 mmol, 10.00 equiv), the reaction mixture was stirred for 24 h at room temperature, and the precipitate was filtered off and washed with 1,4-dioxane. The crude product was purified in portions (approx. 250 mg per separation) by preparative HPLC (water/MeOH 5 - 95% over 1 h). **38d** (371 mg, 0.95 mmol, 61%) was obtained as its hydrochloride in the form of a white solid.

^{1}H NMR (400 MHz, DMSO-d_6, 300 K): δ (ppm) = 12.27 (s, 1H), 10.69 (s, 1H), 9.15 (br s, 1H), 8.83 (s, 1H), 8.80 (br s, 1H), 8.06 (d, ^4J = 2.1 Hz, 1H), 7.71 (dd, ^3J = 8.5, ^4J =2.1 Hz, 1H), 7.59 (dd, ^3J = 3.4, ^3J = 2.3 Hz, 1H), 7.55 (d, ^3J = 8.2 Hz, 1H), 6.36 (dd, ^3J = 3.4, ^3J = 1.8 Hz, 1H), 3.33 (d, ^2J = 12.6 Hz, 2H), 2.96 (d, ^2J = 11.2 Hz, 1H), 2.90 (d, ^2J = 11.9 Hz, 1H), 2.76 (sm, 1H), 2.02 (m, 2H), 1.92-1.89 (m, 1H), 1.86-1.82 (m, 1H); ^{13}C NMR (100 MHz, DMSO-d_6, 300 K): δ (ppm) = 172.8, 155.4, 151.7, 150.5, 140.9, 131.7, 131.4, 131.1, 127.5, 119.8, 117.6, 116.6, 100.0, 42.3, 39.8, 24.9; HR-MS *m/z* $[M+H]^+$ calcd. for $C_{18}H_{18}ClN_5OH$: 356.1273, found: 356.1259; Anal. calcd. for $C_{18}H_{18}ClN_5O$ * HCl x 1.5 H_2O : C 51.56, H 5.29, N 16.70, found: C 51.70, H 5.43, N 16.67; mp: 208.8 °C.

N-(Piperidin-4-ylmethyl)-4-(1*H*-pyrrolo[2,3-b]pyridin-4-yl)aniline formate (39a)

N-(4-(1*H*-Pyrrolo[2,3-*b*]pyridin-4-yl)phenyl)piperidine-4-carboxamide dihydrochloride (**38a**) (200 mg, 0.56 mmol, 1.00 equiv) was suspended in dry THF (4 mL) at 0 °C under an argon atmosphere. 2 M LAH in THF (0.84 mL, 1.68 mmol, 3.00 equiv) was then added dropwise over a period of 5 min. and, after completion of the addition, the reaction was heated under reflux for 16 h. After cooling to room temperature, EtOAc (3 mL) was carefully added. After the gas evolution had subsided, saturated aqueous Na-K-tartrate solution (6 mL) was added with vigorous stirring. After 3 h, the organic phase was separated and extracted with aqueous 1 M HCl solution (2 x 4 mL). The combined aqueous phases were adjusted to pH 1 with aqueous 4 M HCl solution and then washed with DCM (3 x 6 mL). The aqueous phase was adjusted to pH 14 with aqueous 50% NaOH solution and then extracted with DCM/*i*-PrOH (9:1, 6 x 10 mL). The combined organic phases were dried over $MgSO_4$, filtered, and then concentrated under reduced pressure. The residue was taken up in MeOH and applied to Celite® using the dry-load method. Purification was performed by flash chromatography on a C18 cartridge [water(+1% HCOOH)/CH_3CN(+1% HCOOH) 5 - 95% over 33.5 cv]. The solid obtained was purified again by flash chromatography under the same conditions. *N*-(Piperidin-4-ylmethyl)-4-(1*H*-pyrrolo[2,3-b]pyridin-4-yl)aniline formate (**39a**) (73 mg, 0.21 mmol, 37%) was obtained as a beige-colored solid.

^{1}H NMR (400 MHz, DMSO-d_6, 300 K): δ (ppm) = 11.61 (s, 1H), 8.31 (s, 2H), 8.17 (d, ^3J = 4.1 Hz, 1H), 7.55 (d, ^3J = 7.8 Hz, 2H), 7.45 (d, ^3J = 2.3 Hz, 1H), 7.06 (d, ^3J = 4.8 Hz, 1H), 6.74 (d, ^3J = 8.2 Hz, 2H), 6.61 (d, ^3J = 2.5 Hz, 1H), 6.10 (br s, 1H), 3.26 (br s, 2H), 3.01 (br s, 2H), 2.82 (br s, 2H), 1.89 (br s, 3H), 1.35 (s, 2H); ^{13}C NMR (125 MHz, DMSO-d_6, 300 K): δ (ppm) = 149.1, 142.7, 140.8, 128.9, 125.54, 125.47, 125.2, 116.6, 112.8, 112.0, 99.3, 47.8, 42.9, 32.9, 26.7; HR-MS *m/z* $[M+H]^+$ calcd. for $C_{19}H_{22}N_4H$: 307.1917, found: 307.1910; Purity was determined by qNMR using 1,2,4,5-tetrachloro-3-nitro-benzene as internal standard: 95.7%; mp: decomposition > 260 °C.

3-Chloro-*N*-(piperidin-4-ylmethyl)-4-(1*H*-pyrrolo[2,3-b]pyridin-4-yl)aniline formate (39b)

N-(3-Chloro-4-(1*H*-pyrrolo[2,3-b]pyridin-4-yl)phenyl)piperidine-4-carboxamide (**38b**) (150 mg, 0.42 mmol, 1.00 equiv) was suspended in dry THF (6 mL) at 0 °C under an argon atmosphere. 3.5 M Sodium bis(2-methoxy-ethoxy)aluminum dihydride (Red-Al®) in toluene (0.60 mL, 2.11 mmol, 5.00 equiv) was added dropwise over a period of 5 min. After completion of the addition, the reaction was heated under reflux for 16 h. After the reaction had cooled to room temperature, EtOAc (2 mL) was carefully added. After the gas evolution had subsided, saturated aqueous Na-K-tartrate solution (6 mL) was added with vigorous stirring. After 3 h, the organic phase was separated and extracted with aqueous 1 M HCl solution (2 x 4 mL). The combined aqueous phases were adjusted to pH 1 with aqueous 4 M HCl solution and then washed with DCM (3 x 6 mL). The aqueous phase was adjusted to pH 14 with aqueous 50% NaOH solution and then extracted with DCM/*i*-PrOH (9:1, 6 x 10 mL). The combined organic phases were dried over $MgSO_4$, filtered, and then concentrated under reduced pressure. The residue was taken up in methanol and adsorbed onto Celite®. Purification was carried out by flash chromatography on a C18 cartridge [water(+1% HCOOH)/MeCN(+1% HCOOH) 5 - 95% over 33.5 cv(column volume)]. The solid obtained was purified again by flash chromatography under the same conditions. **39b** (70 mg, 0.18 mmol, 43%) was obtained as formate in form of a beige-colored solid.

^{1}H NMR (300 MHz, DMSO-d_6, 300 K): δ (ppm) = 11.68 (s, 1H), 8.36 (s, 1H), 8.20 (d, ^3J = 5.0 Hz, 1H), 7.44 (s, 1H), 7.21 (d, ^3J = 8.3 Hz, 1H), 6.98 (d, ^3J = 4.8 Hz, 1H), 6.76 (s, 1H), 6.68 (d, ^3J = 7.7 Hz, 1H), 6.32 (s, 1H), 6.22 (d, ^3J = 2.8 Hz, 1H), 3.24 (br s, 2H), 2.99 (br s, 2H), 2.80 (br s, 2H), 1.86 (br s, 3H), 1.35 (br s, 2H); ^{13}C NMR (100 MHz, DMSO-d_6, 300 K): δ (ppm) = 149.9, 148.7, 142.0, 139.1, 132.0, 131.9, 125.8, 123.2, 118.8, 116.3, 112.0, 110.9, 99.5, 47.9, 43.2, 33.4, 27.3; HR-MS *m/z* $[M+H]^+$ calcd. for $C_{19}H_{21}ClN_4H$: 341.1528, found: 341.1517; Purity was determined by qNMR using 1,2,4,5-tetrachloro-3-nitro-benzene as internal standard: 95.1%; mp: 195.9 °C.

References

[1] M. D. Winn *et al.*, “Overview of the CCP4 suite and current developments,” *Acta Cryst D*, vol. 67, no. 4, pp. 235–242, Apr. 2011, doi: 10.1107/S0907444910045749.

[2] M. G. Rossmann and E. Arnold, *International tables for crystallography volume F: crystallography of biological macromolecules*. Wiley Online Library, 2001.

[3] R. A. Laskowski, M. W. MacArthur, D. S. Moss, and J. M. Thornton, “PROCHECK: a program to check the stereochemical quality of protein structures,” *Applied Crystallography*, vol. 26, no. 2, pp. 283–291, 1993.

[4] P. L. Kramer, K. Buchthal, D. Sydow, K. Sonja Leo, and A. Volkamer, “CustomKinFragLib: Filtering the Kinase-Focused Fragmentation Library,” *ACS Omega*, 2026.

[5] O. P. Van Linden, A. J. Kooistra, R. Leurs, I. J. De Esch, and C. De Graaf, “KLIFS: a knowledge-based structural database to navigate kinase–ligand interaction space,” *Journal of medicinal chemistry*, vol. 57, no. 2, pp. 249–277, 2014.

[6] BioSolveIT GmbH, Sankt Augustin, Germany, “FlexX.” 2023. [Online]. Available: https://www.biosolveit.de/flexx

[7] BioSolveIT GmbH, Sankt Augustin, Germany, “SeeSAR.” 2023. [Online]. Available: https://www.biosolveit.de/SeeSAR

[8] G. BioSolveIT GmbH Sankt Augustin, “HYDE.” 2023. [Online]. Available: https://www.biosolveit.de/HYDE

[9] F. Sievers *et al.*, “Fast, scalable generation of high-quality protein multiple sequence alignments using Clustal Omega,” *Molecular systems biology*, vol. 7, no. 1, p. 539, 2011.

[10] L. J. Mullan and A. J. Bleasby, “Short EMBOSS user guide,” *Briefings in bioinformatics*, vol. 3, no. 1, pp. 92–94, 2002.

[11] S. F. Altschul, W. Gish, W. Miller, E. W. Myers, and D. J. Lipman, “Basic local alignment search tool,” *Journal of molecular biology*, vol. 215, no. 3, pp. 403–410, 1990.

[12] D. Sydow, P. Schmiel, J. Mortier, and A. Volkamer, “KinFragLib: Exploring the Kinase Inhibitor Space Using Subpocket-Focused Fragmentation and Recombination,” *J. Chem. Inf. Model.*, vol. 60, no. 12, pp. 6081–6094, Dec. 2020, doi: 10.1021/acs.jcim.0c00839.

[13] R. Roskoski Jr, “Classification of small molecule protein kinase inhibitors based upon the structures of their drug-enzyme complexes,” *Pharmacological research*, vol. 103, pp. 26–48, 2016.

[14] C. E. Shannon, “A mathematical theory of communication,” *The Bell system technical journal*, vol. 27, no. 3, pp. 379–423, 1948.

[15] R. Cedillo-González and J. L. Medina-Franco, “Diversity and chemical space characterization of inhibitors of the epigenetic target G9a: A chemoinformatics approach,” *ACS omega*, vol. 8, no. 33, pp. 30694–30704, 2023.

[16] G. W. Bemis and M. A. Murcko, “The properties of known drugs. 1. Molecular frameworks,” *Journal of medicinal chemistry*, vol. 39, no. 15, pp. 2887–2893, 1996.

[17] G. Maggiora, M. Vogt, D. Stumpfe, and J. Bajorath, “Molecular similarity in medicinal chemistry: miniperspective,” *Journal of medicinal chemistry*, vol. 57, no. 8, pp. 3186–3204, 2014.

[18] B. Zdrazil *et al.*, “The ChEMBL Database in 2023: a drug discovery platform spanning multiple bioactivity data types and time periods,” *Nucleic acids research*, vol. 52, no. D1, pp. D1180–D1192, 2024.

[19] ChEMBL, “ChEMBL33 download.” 2023. [Online]. Available: https://doi.org/10.6019/CHEMBL.database.33

[20] RDKit, “RDKit Open-source cheminformatics.” 2023. [Online]. Available: https://www.rdkit.org

[21] RDKit, “RDKit rdFingerprintGenerator module.” 2023. [Online]. Available: https://www.rdkit.org/docs/source/rdkit.Chem.rdFingerprintGenerator.html

[22] P. M. Matos, W. Lewis, J. C. Moore, and R. A. Stockman, “Sulfonimidates: Useful synthetic intermediates for sulfoximine synthesis via C–S bond formation,” *Organic Letters*, vol. 20, no. 12, pp. 3674–3677, 2018.

[23] J. L. G. Ruano, A. Parra, F. Yuste, and V. M. Mastranzo, “Mild and General Method for the Synthesis of Sulfonamides,” *Synthesis*, vol. 2008, no. 2, pp. 311–319, Jan. 2008, doi: 10.1055/s-2007-1000850.
[24] H.-J. Boehm *et al.*, “Novel inhibitors of DNA gyrase: 3D structure based biased needle screening, hit validation by biophysical methods, and 3D guided optimization. A promising alternative to random screening,” *Journal of medicinal chemistry*, vol. 43, no. 14, pp. 2664–2674, 2000.
[25] G. Rueedi *et al.*, “Discovery and structure–activity relationship of cadazolid: a first-in-class quinoxolidinone antibiotic for the treatment of clostridioides difficile infection,” *Journal of Medicinal Chemistry*, vol. 67, no. 11, pp. 9465–9484, 2024.
[26] T. Jackson, L. L. Woo, M. N. Trusselle, A. Purohit, M. J. Reed, and B. V. Potter, “Non-steroidal aromatase inhibitors based on a biphenyl scaffold: synthesis, in vitro sar, and molecular modelling,” *ChemMedChem: Chemistry Enabling Drug Discovery*, vol. 3, no. 4, pp. 603–618, 2008.
[27] J. Kwiatkowski *et al.*, “Fragment-Based Drug Discovery of Potent Protein Kinase C Iota Inhibitors,” *J. Med. Chem.*, vol. 61, no. 10, pp. 4386–4396, May 2018, doi: 10.1021/acs.jmedchem.8b00060.
